# TBAB-Catalyzed 1,6-Conjugate Sulfonylation of *para*-Quinone Methides: A Highly Efficient Approach to Unsymmetrical *gem*-Diarylmethyl Sulfones in Water †

**DOI:** 10.3390/molecules25030539

**Published:** 2020-01-26

**Authors:** Zhang-Qin Liu, Peng-Sheng You, Liang-Dong Zhang, Da-Qing Liu, Sheng-Shu Liu, Xiao-Yu Guan

**Affiliations:** Key Laboratory of Applied Chemistry of Chongqing Municipality, College of Chemistry and Chemical Engineering, Southwest University, Chongqing 400715, China

**Keywords:** sulfa-1,6-conjugated addition, unsymmetrical diarylmethyl sulfones, green and sustainable chemistry, synthesis in water

## Abstract

A highly efficient sulfonylation of *para*-quinone methides with sulfonyl hydrazines in water has been developed on the basis of the mode involving a tetrabutyl ammonium bromide (TBAB)-promoted sulfa-1,6-conjugated addition pathway. This reaction provides a green and sustainable method to synthesize various unsymmetrical diarylmethyl sulfones, showing good functional group tolerance, scalability, and regioselectivity. Further transformation of the resulting diarylmethyl sulfones provides an efficient route to some functionalized molecules.

## 1. Introduction

**Figure 1:**
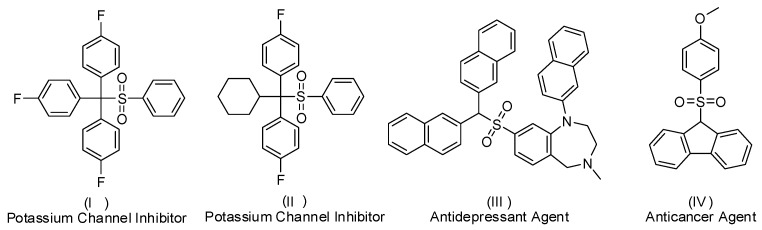
Representative biologically active diarylmethyl sulfone derivatives.

As a class of sulfur-containing compounds, sulfones are widely used in organic synthesis, pharmaceuticals, agrochemicals, and materials science [1,2,3,4,5,6,7]. Among the sulfone family, diarylmethyl sulfones hold an essential position in biologically important compounds that show various biological activities, such as potassium channel inhibitory activity [8], as well as antidepressant [9] and anticancer properties (Figure 1) [10]. Additionally, diarylmethyl sulfones can be applied as useful intermediates for synthetic applications owing to the versatile reactivities of the sulfonyl group activated carbanions [1,11,12,13,14].

Although a number of methodologies have been developed for the synthesis of diarylmethyl sulfones, most of the reported methods suffer from the harsh reaction conditions, multi-step procedures, and requirements of expensive metal catalysts or potentially toxic organic solvents [15,16,17,18], which make them unsustainable and environmentally unfavorable.

Concerns about the environmental issues caused by the influence of human society have currently become prevalent and ubiquitous. Engagement on green and sustainable methods of chemical synthesis has emerged as a pioneering realm [19,20,21,22,23] garnering of enormous attention of chemists and biologists. Among them, chemical reactions “in water” [24] offered a novel way to green and sustainable synthesis. Water, compared to the traditional organic solvents, possesses distinctive properties such as safety, innocuousness, high heat capacity, extensive hydrogen bonding, and redox stability [24,25,26]. Consequently, water has been gradually accepted [27,28,29,30,31,32,33] as a desirable reaction medium since the first water-promoted Diels–Alder reaction reported by Breslow [34] in 1980.

**Scheme 1:**
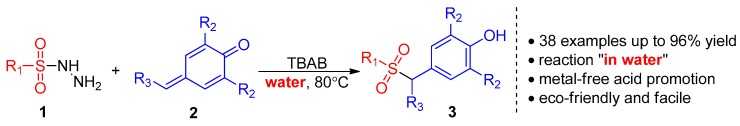
Synthesis of unsymmetrical *gem*-diarylmethyl sulfones via sulfa-1,6-conjugated addition in water.

The C-S bond formation reaction is still an intriguing field attributed to the versatile block-building usages [35,36] and the bioactive agents [37,38] of sulfur-containing compounds. Thus, substantial endeavors have been focused on the achievement of the efficient, expedient, and low-cost approach. With our keen interest in the sulfa-1,6-conjugated addition reaction [15,39,40], we try to combine the green and sustainable chemical synthetic concept and the C-S bonding formation reactions. Herein, we disclose a highly efficient tetrabutyl ammonium bromide (TBAB)-promoted sulfonylation of *para*-quinone methide (*p*-QM) with sulfonyl hydrazines to afford unsymmetrical *gem*-diarylmethyl sulfones via a sulfa-1,6-conjugated addition pathway. All these reactions were performed smoothly in water under mild conditions (Scheme 1).

## 2. Results and Discussion

### 2.1. Optimization of Reaction Conditions

In our preliminary study, the investigation of the reaction conditions was carried out with 4-methylbenzenesulfonohydrazide **1a** and *para*-quinone methides *(p-QM)* **2a** as model substrates (Table 1). Initially, the reaction was explored with different solvents in absence of a catalyst. When the reaction was carried out at 80 °C in tetrahydrofuran (THF), the desired 1,6-sulfa-conjugated adduct was obtained in mediate yield (50%). Other organic solvents including Et_2_O, toluene, dichloroethane (DCE), dioxane, and dimethylsulfoxide (DMSO) were proved to be unsuitable for the reaction (Table 1, entry 1–6). It was found that protic polar solvent had an obvious promotion effect on the reaction. When H_2_O and EtOH were employed, the yield was increased slightly to 61% and 73%, respectively (Table 1, entry 7–8). Although EtOH had a better effect than water, taking into consideration the “green and sustainable chemistry” perspective, we chose H_2_O as a solvent. Subsequently, we focused on catalyst screening to improve the yield of the water mediated C-S bond formation reaction. Unfortunately, heterogeneous transition metal nano-catalysts such as Pd/C, Pd/TiO_2_, Au/TiO_2_, and Pt/C had little promotion effect on the reaction (Table 1, entry 9–12). Inspired by the better result of EtOH, phase transfer catalysts were selected to improve the solubility of organic substrate **1a** and **2a** in water, which may have a positive influence on the reaction (Table 1, entry 13–17). To our delight, in presence of 10% mol tetrabutylammonium bromide (TBAB), the yield was improved sharply to 82% (Table 1, entry 16). Based on the result, we continuously investigated the effect of other reaction parameters including temperature, reaction time, and reactant ratio. We were pleased to find that the yield was markedly increased, as the reactant ratio of **2a**:**1a** was increased from 1 to 1.5. Thus, the desired *gem*-diarylmethyl sulfone **3aa** was obtained in 96% yield by performing the TBAB-catalyzed sulfonylation of 4-methylbenzenesulfonohydrazide **1a** to *p*-QM **2a** at 80 °C in 12 h (Table 1, entry 22). Given either a lower or higher temperature, the yields decreased (Table 1, entry 18–20).

### 2.2. Reaction Scope

**Figure 2:**
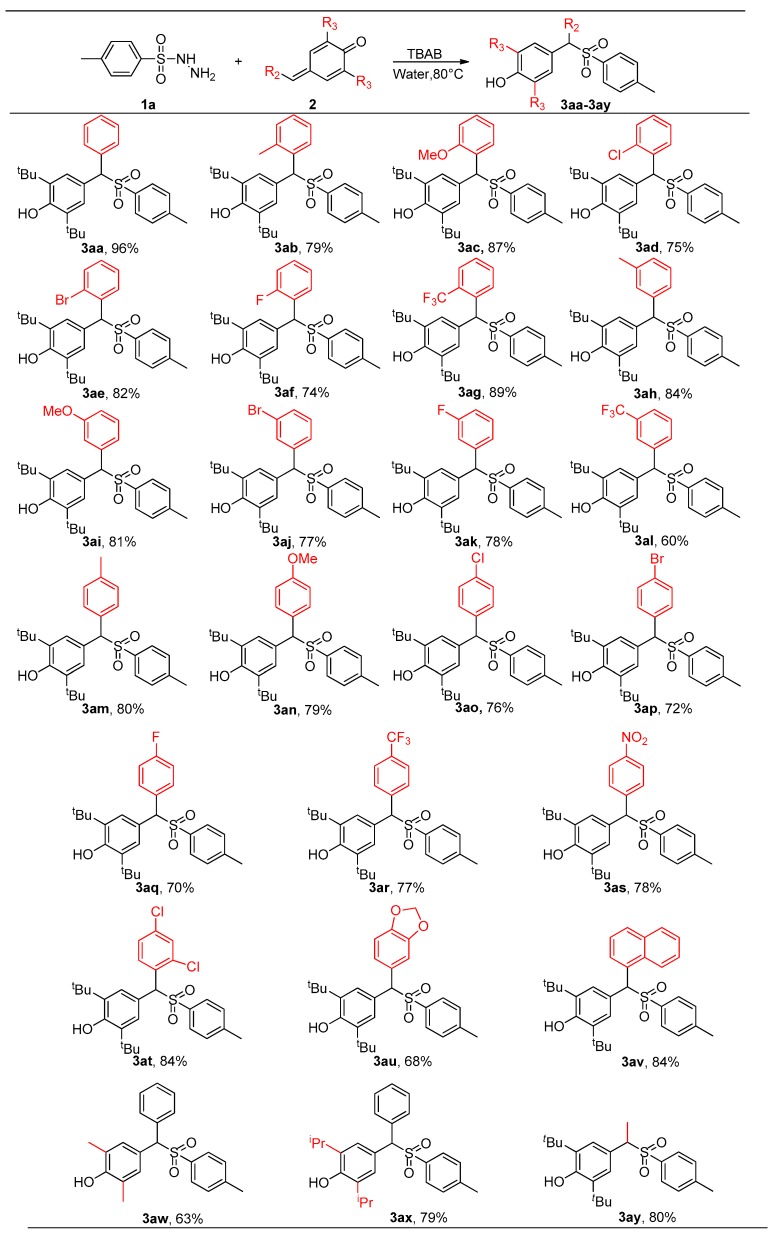
Tetrabutylammonium bromide (TBAB)-catalyzed 1,6-conjugate sulfonylation for synthesis of *gem*-diarylmethyl sulfones **3aa**–**3ay.** ^a^ The reaction conditions: sulfonyl hydrazides **1a** (0.20 mmol), *p*-QM **2** (0.30 mmol), TBAB (0.02 mmol) in 1 mL of H_2_O, 80 °C, 12 h, ^b^ Isolated yield base on **1a**.

**Figure 3:**
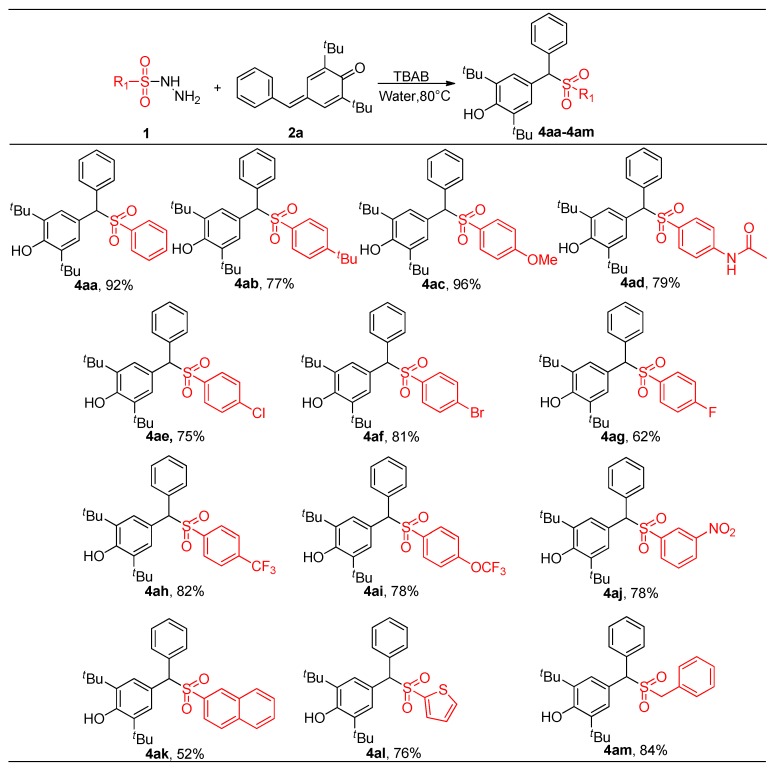
TBAB catalyzed 1,6-conjugate sulfonylation for synthesis of *gem*-diarylmethyl sulfones **4aa**–**4am.** ^a^ The reaction conditions: sulfonyl hydrazides **1** (0.20 mmol), *para*-quinone methide (*p*-QM) **2a** (0.30 mmol), TBAB (0.02 mmol) in 1 mL of H_2_O, 80°C, 12h, ^b^ isolated yield base on **1**.

Based on the optimized reaction conditions, the generality of this 1,6-conjugate sulfonylation reaction of sulfonyl hydrazines to *para*-quinone methides was investigated. Some of the results are summarized in Figure 2 and Figure 3.

A number of *para*-quinone methide derivatives **2** bearing different substituents were explored with 4-methylbenzenesulfonohydrazide **1a** under standard reaction condition (Figure 2). Various substituent groups, such as electron-donating groups (-Me, -OMe), electron-withdrawing groups (-CF_3_, -NO_2_), and halogen atoms (F, Cl, Br), on the *p*-QM derivatives’ aryl ring at the *ortho* (**2ab**–**2ag**), *meta* (**2ah**–**2al**), and *para* (**2am**–**2as**) positions were well tolerated by the reaction to provide the corresponding sulfonylation adducts in good to excelled yields. Furthermore, non-substituted phenyl substrate **2aa**, disubstituted 2,4-dichlorophenyl substrate **2at**, polycyclic aromatic substrate **2av**, heteroaromatic substrates **2au**, and aliphatic substrate **2ay** led to the desired product in 63–95% yields, implying the well-tolerated property of the substrates in this reaction. Moreover, *p*-QMs derived from 2,6-dimethylphenol **2aw** and 2,6-diisopropylphenol **2ax** were also compatible to afford the corresponding adducts, while the former was in lower yield (**3aw**, 63%) probably due to its small steric hindrance around the phenolic hydroxyl group.

Consequentially, we investigated the substitute effects of sulfonyl hydrazines by using 4-benzylidene-2,6-di-*tert*-butylcyclohexa-2,5-dienone **2a** as model substrate under optimized reaction condition (Figure 3). In general, a wide range of sulfonyl hydrazines **1** bearing *meta* and *para* substituent group including electron-donating groups (-Me, -OMe, -NHAc, -OCF_3_), electron-withdrawing groups (-CF_3_, -NO_2_), and halogen atom (F, Cl, Br) reacted smoothly with *p*-QM **2a**, affording the adducts in 62–96% yields. In addition, phenyl **4aa**, *p*-*tert*-butyl phenyl **4ab**, 2-naphthyl **4ak**, thienyl **4al** and aliphatic benzyl substituted sulfonyl hydrazines were also tolerated by the reaction to produce the final products in 52–92% yields.

### 2.3. Proposed Mechanism

**Figure 4:**
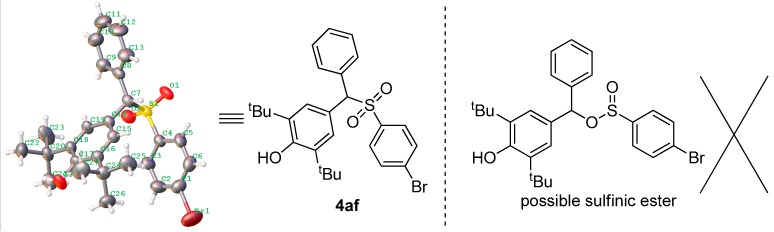
Crystal structure of diarylmethyl sulfone **4af** and possible sulfinic ester.

The chemical structure of diarylmethyl sulfones **3** and **4** were characterized by nuclear magnetic resonance spectroscopies (Supplementary Materials). Because there is another possible sulfinic ester adduct, the two potential products cannot be distinguished by NMR. To further identify the structure of the products, **4af** was selected as a representative compound, and the sulfone structure was unequivocally confirmed by single crystal X-ray diffraction analysis, as shown in Figure 4 (CCDC No. 1531154).

**Scheme 2:**
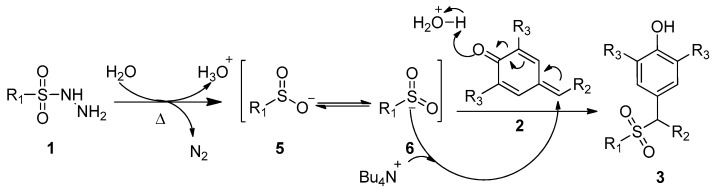
Probable mechanism of the sulfa-1,6-conjugate reactions in water.

On the basis of the above observations, we tentatively propose a plausible reaction mechanism (Scheme 2). First, the sulfonyl hydrazines **1** decomposed into sulfinyl anion **5** with the N_2_ released and hydronium generation under the heating condition. Intermediate **5** has a resonate equilibrium with sulfur-centered anion **6** in water. As a more reactive species, the sulfur-centered anion **6** attacks the electrophilic atom of the resonated structure of *p*-QM **2** with the help of tetrabutylammonium bromide (TBAB). Finally, driven by the aromatization force, the target product is obtained.

### 2.4. Derivatives of Products

**Scheme 3:**
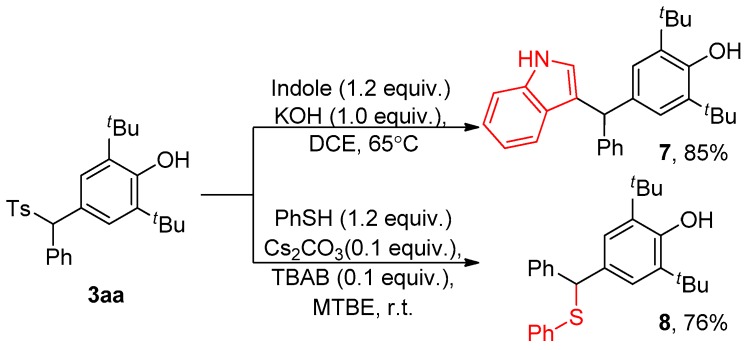
Synthetic utility of diarylmethyl sulfone **3aa**.

To further demonstrate the synthetic utility of this protocol, the transformations of products were then explored (Scheme 3). A carbon-carbon bond formation between **3aa** and indole led to the generation of unsymmetrical triarylmethane **7**. Furthermore, base-promoted carbon-sulfur bond formation between **3aa** and thiophenol proceeded to the facial delivery of unsymmetrical *gem*-diarylmethyl thioether **8**. Moreover, the yield of products of both synthesis routines reached around 80%, implying the potential and feasibility of further application of **3aa**.

## 3. Materials and Methods

### 3.1. General Information

^1^H-NMR, ^13^C-NMR spectra were obtained utilizing a Bruker 600 and 400 MHz instrument and reported in CDCl_3_ or DMSO(d^6^). ^1^H and ^13^CNMR chemical shifts are reported in ppm relative to either TMS (^1^H) (δ = 0 ppm) as an internal standard or the residual solvent peak as following: CDCl_3_ = 7.26 (^1^H-NMR), (CD_3_)_2_SO = 2.50 (^1^H-NMR), CDCl_3_ = 77.16 (^13^C-NMR), (CD_3_)_2_SO = 40.00 (^13^C-NMR). HRMS were performed on a Bruker Impact II 10200 instrument. Commercially available chemicals and solvents were purchased from Adamas-beta, Energy Chemical, Chongqing Chuandong Chemical, and Chengdu Kelong Chemical. The corresponding compounds were synthesized according to the methods reported in the literature. Analytical thin-layer chromatography (TLC) was performed on silicycle silica gel plates with F-254 indicator, and compounds were visualized by irradiation with UV light. Chromatography was carried out using silica gel 300–400 mesh.

### 3.2. Experiment

#### 3.2.1. Representative Procedure for Synthesis of Gem-Diarylmethyl Sulfones

To a solution of corresponding 4-(arylmethylidene)-2,6-di-*tert*-butylcyclohexa-2,5-dienone (0.3 mmol) in 1 mL water, the corresponding arylsulfonohydrazide (0.2 mmol) and TBAB (0.02 mmol) were added. The mixture was stirred at 80°C. The reaction was monitored by TLC. After complete reaction, the mixture was extracted by ethyl acetate, dried over by anhydrous magnesium sulfate, and concentrated in vacuo. The crude product was then purified by flash column chromatography on silica gel (gradient eluent of PE/EA = 30:1–10:1) to gain the corresponding product.

*2,6-Di-tert-butyl-4-(phenyl(tosyl)methyl)phenol* (**3aa**): [15] Pale yellow solid; 96% yield. ^1^H-NMR (600 MHz, CDCl_3_) δ 7.61 (d, *J* = 7.1 Hz, 2H), 7.42 (d, *J* = 8.0 Hz, 2H), 7.32 (dq, *J* = 14.2, 6.9 Hz, 3H), 7.16 (s, 2H), 7.13 (d, *J* = 7.9 Hz, 2H), 5.20 (s, 1H), 5.16 (s, 1H), 2.36 (s, 3H), 1.35 (s, 18H); ^13^C-NMR (151 MHz, CDCl_3_) δ 154.08 (s), 144.00 (s), 135.94 (s), 135.77 (s), 133.66 (s), 130.00 (s), 129.10 (s), 129.05 (s), 128.62 (s), 128.36 (s), 127.10 (s), 123.57 (s), 76.90 (s), 34.31 (s), 30.16 (s), 21.48 (s).

*2,6-Di-tert-butyl-4-(o-tolyl(tosyl)methyl)phenol* (**3ab**): [15] Pale yellow solid; 76% yield. ^1^H-NMR (600 MHz, CDCl_3_) δ 8.23 (d, *J* = 7.8 Hz, 1H), 7.44 (d, *J* = 8.0 Hz, 2H), 7.30 (t, *J* = 7.5 Hz, 1H), 7.19 (t, *J* = 7.4 Hz, 1H), 7.14 (d, *J* = 8.0 Hz, 2H), 7.13 (s, 2H), 7.08 (d, *J* = 7.5 Hz, 1H), 5.43 (s, 1H), 5.21 (s, 1H), 2.37 (s, 3H), 2.17 (s, 3H), 1.34 (s, 18H). ^13^C-NMR (151 MHz, CDCl_3_) δ 153.99 (s), 143.87 (s), 138.20 (s), 135.84 (s), 130.49 (s), 129.84 (s), 129.33 (s), 129.09 (s), 128.99 (s), 127.03 (s), 123.75 (s), 76.67 (s), 34.28 (s), 30.13 (s), 21.47 (s), 21.09 (s).

*2,6-Di-tert-butyl-4-((2-methoxyphenyl)(tosyl)methyl)phenol* (**3ac**): [15] Pale yellow solid; 87% yield. ^1^H-NMR (600 MHz, CDCl_3_) δ 8.14 (d, *J* = 7.6 Hz, 1H), 7.44 (d, *J* = 7.9 Hz, 2H), 7.22–7.25 (m, 3H), 7.13 (d, *J* = 7.8 Hz, 2H), 7.04 (t, *J* = 7.5 Hz, 1H), 6.73 (d, *J* = 8.2 Hz, 1H), 5.93 (s, 1H), 5.19 (s, 1H), 3.60 (s, 3H), 2.36 (s, 3H), 1.36 (s, 18H). ^13^C-NMR (151 MHz) δ 156.98 (s), 153.95 (s), 143.67 (s), 136.30 (s), 135.69 (s), 129.86 (s), 129.39 (s), 129.08 (s), 128.82 (s), 127.36 (s), 123.53 (s), 122.54 (s), 120.69 (s), 110.79 (s), 66.98 (s), 55.56 (s), 34.30 (s), 30.17 (s), 21.46 (s).

*2,6-Di-tert-butyl-4-((2-chlorophenyl)(tosyl)methyl)phenol* (**3ad**): [15] Pale yellow solid; 75% yield. ^1^H-NMR (600 MHz, CDCl_3_) δ 8.35 (d, *J* = 7.9 Hz, 1H), 7.46 (d, *J* = 8.1 Hz, 2H), 7.37 (t, *J* = 7.6 Hz, 1H), 7.29 (d, *J* = 8.0 Hz, 1H), 7.23 (t, *J* = 7.7 Hz, 1H), 7.16 (s, 3H), 7.15 (s, 1H), 5.88 (s, 1H), 5.23 (s, 1H), 2.37 (s, 3H), 1.35 (s, 18H). ^13^C-NMR (151 MHz, CDCl_3_) δ 153.20 (s), 143.25 (s), 134.90 (s), 134.72 (s), 133.87 (s), 131.18 (s), 129.10 (s), 128.80 (s), 128.38 (s), 128.17 (s), 128.01 (s), 126.25 (s), 126.05 (s), 121.54 (s), 70.08 (s), 33.28 (s), 29.11 (s), 20.52 (s).

*4-((2-Bromophenyl)(tosyl)methyl)-2,6-di-tert-butylphenol* (**3ae**): [15] Pale yellow solid; 82% yield. ^1^H-NMR (600 MHz, CDCl_3_) δ 8.35 (d, *J* = 7.8 Hz, 1H), 7.47 (d, *J* = 7.9 Hz, 3H), 7.41 (t, *J* = 7.6 Hz, 1H), 7.18 (s, 2H), 7.15 (m, 3H), 5.90 (s, 1H), 5.23 (s, 1H), 2.37 (s, 3H), 1.36 (s, 18H). ^13^C-NMR (151 MHz, CDCl_3_) δ 154.19 (s), 144.25 (s), 135.92 (s), 135.77 (s), 133.88 (s), 133.17 (s), 130.19 (s), 129.64 (s), 129.18 (s), 129.01 (s), 127.69 (s), 127.24 (s), 126.02 (s), 122.56 (s), 73.83 (s), 34.29 (s), 30.13 (s), 21.51 (s).

*2,6-Di-tert-butyl-4-((2-fluorophenyl)(tosyl)methyl)phenol* (**3af**): [15] Pale yellow solid; 74% yield. ^1^H-NMR (600 MHz, CDCl_3_) δ 8.21 (t, *J* = 7.2 Hz, 1H), 7.46 (d, *J* = 8.0 Hz, 2H), 7.29 (dd, *J* = 13.2, 6.3 Hz, 1H), 7.27–7.22 (m, 1H), 7.16 (s, 3H), 7.15 (s, 1H), 6.96 (t, *J* = 9.1 Hz, 1H), 5.63 (s, 1H), 5.24 (s, 1H), 2.37 (s, 3H), 1.35 (s, 18H). ^13^C-NMR (151 MHz, CDCl_3_) δ 160.61 (d, *J_C-F_* = 247.4 Hz), 154.22 (s), 144.23 (s), 135.96 (s), 135.57 (s), 130.15 (d, *J_C-F_* = 1.6 Hz), 129.97 (d, *J_C-F_* = 8.6 Hz), 129.14 (s), 129.05 (s), 127.19 (s), 124.30 (d, *J_C-F_* = 3.6 Hz), 122.73 (s), 121.57 (d, *J_C-F_* =13.5 Hz), 115.50 (d, *J_C-F_* = 23.0 Hz), 67.18 (d, *J_C-F_* = 4.2 Hz), 34.29 (s), 30.10 (s), 21.50 (s).

*2,6-Di-tert-butyl-4-(tosyl(2-(trifluoromethyl)phenyl)methyl)phenol* (**3ag**): [15] Pale yellow solid; 89% yield. ^1^H-NMR (600 MHz, CDCl_3_) δ 8.61 (d, *J* = 7.9 Hz, 1H), 7.68 (t, *J* = 7.7 Hz, 1H), 7.62 (d, *J* = 7.8 Hz, 1H), 7.44 (m, 3H), 7.15 (m, 4H), 5.59 (s, 1H), 5.23 (s, 1H), 2.36 (s, 3H), 1.34 (s, 18H). ^13^C-NMR (151 MHz, CDCl_3_) δ 154.20 (s), 144.34 (s), 135.87 (s), 135.59 (s), 133.00 (s), 131.99 (s), 130.47 (s), 129.18 (s), 129.10 (s), δ 129.64 – 128.96 (m), 128.25 (s), 127.10 (s), 126.45 (q, *J_C-F_* = 5.9 Hz), 124.12 (q, *J_C-F_* = 274.4 Hz).122.73 (s), 71.00 (s), 34.29 (s), 30.10 (s), 21.48 (s).

*2,6-Di-tert-butyl-4-(m-tolyl(tosyl)methyl)phenol* (**3ah**): [15] Pale yellow solid; 84% yield. ^1^H-NMR (600 MHz, CDCl_3_) δ 7.46 (d, *J* = 7.5 Hz, 1H), 7.42 (d, *J* = 7.7 Hz, 2H), 7.39 (s, 1H), 7.28–7.19 (m, 1H), 7.13 (m, 5H), 5.20 (s, 1H), 5.12 (s, 1H), 2.35 (d, *J* = 9.0 Hz, 3H), 2.33 (s, 3H), 1.35 (s, 18H); ^13^C-NMR (151 MHz, CDCl_3_) δ 154.03 (s), 143.92 (s), 138.21 (s), 135.84 (s), 135.81 (s), 133.43 (s), 130.86 (s), 129.12 (s), 128.99 (s), 128.49 (s), 127.10 (s), 126.88 (s), 123.65 (s), 76.92 (s), 34.28 (s), 30.12 (s), 21.46 (s).

*2,6-Di-tert-butyl-4-((3-methoxyphenyl)(tosyl)methyl)phenol* (**3ai**): [15] Pale yellow solid; 81% yield. ^1^H-NMR (600 MHz, CDCl_3_) δ 7.43 (d, *J* = 7.8 Hz, 2H), 7.27–7.23 (m, 1H), 7.19 (d, *J* = 5.4 Hz, 2H), 7.17–7.11 (m, 4H), 6.86 (d, *J* = 8.0 Hz, 1H), 5.21 (s, 1H), 5.13 (s, 1H), 3.79 (s, 3H), 2.36 (s, 3H), 1.35 (s, 18H). ^13^C-NMR (151 MHz, CDCl_3_) δ 159.65 (s), 154.08 (s), 143.99 (s), 135.87 (s), 135.74 (s), 134.93 (s), 129.53 (s), 129.11 (s), 129.03 (s), 127.07 (s), 123.45 (s), 122.39 (s), 115.33 (s), 114.42 (s), 76.78 (s), 55.22 (s), 34.29 (s), 30.13 (s), 21.47 (s).

*4-((3-Bromophenyl)(tosyl)methyl)-2,6-di-tert-butylphenol* (**3aj**): [15] Pale yellow solid; 77% yield. ^1^H-NMR (600 MHz, CDCl_3_) δ 7.69 (s, 1H), 7.66 (d, *J* = 7.8 Hz, 1H), 7.45 (d, *J* = 8.0 Hz, 1H), 7.42 (d, *J* = 8.1 Hz, 2H), 7.23 (t, *J* = 7.9 Hz, 1H), 7.15 (d, *J* = 8.0 Hz, 2H), 7.10 (s, 2H), 5.24 (s, 1H), 5.11 (s, 1H), 2.37 (s, 3H), 1.35 (s, 18H). ^13^C-NMR (151 MHz, CDCl_3_) δ 154.24 (s), 144.32 (s), 136.08 (s), 135.82 (s), 135.35 (s), 133.15 (s), 131.46 (s), 130.07 (s), 129.16 (s), 129.10 (s), 128.34 (s), 127.02 (s), 122.90 (s), 122.55 (s), 76.15 (s), 34.31 (s), 30.10 (s), 21.49 (s).

*2,6-Di-tert-butyl-4-((3-fluorophenyl)(tosyl)methyl)phenol (***3ak**): [15] Pale yellow solid; 78% yield. ^1^H-NMR (400 MHz, CDCl_3_) δ 7.43 (d, *J* = 8.2 Hz, 2H), 7.41–7.36 (m, 2H), 7.31 (td, *J* = 8.0, 6.0 Hz, 1H), 7.15 (d, *J* = 8.0 Hz, 2H), 7.11 (s, 2H), 7.02 (tdd, *J* = 8.4, 2.5, 0.8 Hz, 1H), 5.25 (s, 1H), 5.15 (s, 1H), 2.37 (s, 3H), 1.35 (s, 18H). ^13^C-NMR (151 MHz, CDCl_3_) δ 162.67 (d, *J_C-F_* = 246.5 Hz), 154.23 (s), 144.24 (s), 136.06 (s), 135.91 (d, *J_C-F_* = 7.3 Hz), 135.46 (s), 130.02 (d, *J_C-F_* = 8.3 Hz), 129.13 (s), 129.08 (s), 127.01 (s), 125.71 (d, *J_C-F_* = 2.8 Hz), 123.04 (s), 117.05 (d, *J_C-F_* = 23.0 Hz), 115.35 (d, *J_C-F_* = 21.1 Hz), 76.22 (s), 34.29 (s), 30.10 (s), 21.43 (s).

*2,6-Di-tert-butyl-4-(tosyl(3-(trifluoromethyl)phenyl)methyl)phenol* (**3al**): [15] Pale yellow solid; 60% yield. ^1^H-NMR (600 MHz, CDCl_3_) δ 7.91 (d, *J* = 7.7 Hz, 1H), 7.81 (s, 1H), 7.58 (d, *J* = 7.7 Hz, 1H), 7.49 (t, *J* = 7.8 Hz, 1H), 7.42 (d, *J* = 8.0 Hz, 2H), 7.15 (s, 1H), 7.13 (s, 3H), 5.27 (s, 1H), 5.23 (s, 1H), 2.36 (s, 3H), 1.35 (s, 18H). ^13^C-NMR (151 MHz, CDCl_3_) δ 154.33 (s), 144.47 (s), 136.20 (s), 135.18 (s), 134.71 (s), 133.22 (s), 130.94 (q, *J_C-F_* = 32.5 Hz), 129.20 (s), 129.11 (d, *J_C-F_* =3.5 Hz), 127.20–126.83 (m), 125.29–125.02 (m), 123.90 (q, *J_C-F_* = 272.4 Hz).122.70 (s), 76.29 (s), 34.32 (s), 30.08 (s), 21.45 (s).

*2,6-Di-tert-butyl-4-(p-tolyl(tosyl)methyl)phenol* (**3am**): [15] Pale yellow solid; 80% yield. ^1^H-NMR (600 MHz, CDCl_3_) δ 7.50 (d, *J* = 8.0 Hz, 2H), 7.42 (d, *J* = 8.1 Hz, 2H), 7.15 (d, *J* = 8.2 Hz, 4H), 7.12 (d, *J* = 8.1 Hz, 2H), 5.19 (s, 1H), 5.13 (s, 1H), 2.36 (s, 3H), 2.33 (s, 3H), 1.35 (s, 18H). ^13^C-NMR (151 MHz, CDCl_3_) δ 153.99 (s), 143.87 (s), 138.20 (s), 135.84 (s), 130.49 (s), 129.84 (s), 129.33 (s), 129.09 (s), 128.99 (s), 127.03 (s), 123.75 (s), 76.67 (s), 34.28 (s), 30.13 (s), 21.47 (s), 21.09 (s).

*2,6-Di-tert-butyl-4-((4-methoxyphenyl)(tosyl)methyl)phenol* (**3an**): [15] Pale yellow solid; 79% yield. ^1^H-NMR (600 MHz, CDCl_3_) δ 7.53 (d, *J* = 8.6 Hz, 2H), 7.42 (d, *J* = 8.0 Hz, 2H), 7.15 (s, 2H), 7.12 (d, *J* = 8.0 Hz, 2H), 6.87 (d, *J* = 8.6 Hz, 2H), 5.20 (s, 1H), 5.13 (s, 1H), 3.79 (s, 3H), 2.35 (s, 3H), 1.35 (s, 18H). ^13^C-NMR (151 MHz, CDCl_3_) δ 155.77 (s), 150.04 (s), 139.94 (s), 131.93 (s), 131.87 (s), 127.28 (s), 125.13 (s), 125.08 (s), 123.06 (s), 121.53 (s), 119.87 (s), 110.15 (s), 72.34 (s), 51.33 (s), 30.35 (s), 27.78–23.72 (m), 17.53 (s).

*2,6-Di-tert-butyl-4-((4-chlorophenyl)(tosyl)methyl)phenol* (**3ao**): [15] Pale yellow solid; 81% yield. ^1^H-NMR (600 MHz, CDCl_3_) δ 7.57 (d, *J* = 8.4 Hz, 2H), 7.42 (d, *J* = 8.1 Hz, 2H), 7.32 (d, *J* = 8.4 Hz, 2H), 7.14 (d, *J* = 8.1 Hz, 2H), 7.09 (s, 2H), 5.23 (s, 1H), 5.14 (s, 1H), 2.37 (s, 3H), 1.35 (s, 18H). ^13^C-NMR (151 MHz, CDCl_3_) δ 154.18 (s), 144.23 (s), 136.05 (s), 135.45 (s), 134.54 (s), 132.16 (s), 131.25 (s), 129.15 (s), 129.07 (s), 128.82 (s), 126.96 (s), 123.10 (s), 76.05 (s), 34.30 (s), 30.10 (s), 21.50 (s).

*4-((4-Bromophenyl)(tosyl)methyl)-2,6-di-tert-butylphenol* (**3ap**): [15] Pale yellow solid; 79% yield. ^1^H-NMR (400 MHz, CDCl_3_) δ 7.54–7.45 (m, 4H), 7.42 (d, *J* = 8.2 Hz, 2H), 7.15 (d, *J* = 8.1 Hz, 2H), 7.09 (s, 2H), 5.24 (s, 1H), 5.13 (s, 1H), 2.37 (s, 3H), 1.34 (s, 18H). ^13^C-NMR (101 MHz, CDCl_3_) δ 154.20 (s), 144.28 (s), 136.02 (s), 135.35 (s), 132.67 (s), 131.79 (s), 131.54 (s), 129.17 (s), 129.06 (s), 126.95 (s), 122.98 (s), 122.76 (s), 76.08 (s), 34.30 (s), 30.10 (s), 21.53 (s).

*2,6-Di-tert-butyl-4-((4-fluorophenyl)(tosyl)methyl)phenol* (**3aq**): [15] Pale yellow solid; 70% yield. ^1^H-NMR (600 MHz, CDCl_3_) δ 7.60 (dd, *J* = 8.2, 5.4 Hz, 2H), 7.42 (d, *J* = 8.0 Hz, 2H), 7.14 (d, *J* = 8.0 Hz, 2H), 7.12 (s, 2H), 7.04 (t, *J* = 8.5 Hz, 2H), 5.23 (s, 1H), 5.16 (s, 1H), 2.36 (s, 3H), 1.35 (s, 18H).^13^C-NMR (151 MHz, CDCl_3_) δ 162.79 (d, *J_C-F_* = 248.1 Hz), 154.13 (s), 144.15 (s), 136.03 (s), 135.54 (s), 131.73 (d, *J_C-F_* = 8.2 Hz), 129.44 (d, *J_C-F_* = 3.2 Hz), 129.11 (s), 129.06 (s), 126.97 (s), 123.33 (s), 115.57 (d, *J_C-F_* = 21.5 Hz), 75.98 (s), 34.30 (s), 30.11 (s), 21.47 (s).

*2,6-Di-tert-butyl-4-(tosyl(4-(trifluoromethyl)phenyl)methyl)phenol* (**3ar**): [15] Pale yellow solid; 77% yield. ^1^H-NMR (600 MHz, CDCl_3_) δ 7.78 (d, *J* = 8.1 Hz, 2H), 7.62 (d, *J* = 8.2 Hz, 2H), 7.42 (d, *J* = 8.1 Hz, 2H), 7.15 (d, *J* = 8.0 Hz, 2H), 7.09 (s, 2H), 5.25 (s, 1H), 5.22 (s, 1H), 2.37 (s, 3H), 1.35 (s, 18H). ^13^C-NMR (151 MHz, CDCl_3_) δ 154.29 (s), 144.41 (s), 137.69 (s), 136.17 (s), 135.29 (s), 130.55 (q, *J_C-F_* = 32.6 Hz), 130.28 (s), 129.18 (s), 129.07 (s), 126.97 (s), 125.53 (q, *J_C-F_* = 3.6 Hz), 123.96 (q, *J_C-F_* = 272.1 Hz). 122.83 (s), 76.32 (s), 34.30 (s), 30.08 (s), 21.49 (s).

*2,6-Di-tert-butyl-4-((4-nitrophenyl)(tosyl)methyl)phenol* (**3as**): [15] Pale yellow solid; 72% yield. ^1^H-NMR (400 MHz, CDCl_3_) δ 8.22 (d, *J* = 8.9 Hz, 2H), 7.85 (d, *J* = 8.8 Hz, 2H), 7.43 (d, *J* = 8.3 Hz, 2H), 7.17 (d, *J* = 8.0 Hz, 2H), 7.07 (s, 2H), 5.29 (s, 1H), 5.27 (s, 1H), 2.38 (s, 3H), 1.34 (s, 18H). ^13^C-NMR (151 MHz, CDCl_3_) δ 153.76 (s), 148.51 (d, *J* = 2.9 Hz), 147.13 (s), 143.63 (s), 137.22 (s), 136.63 (s), 129.98 (s), 129.55 (s), 128.21 (s), 127.26 (s), 124.18 (s), 123.50 (s), 61.32 (s), 34.31 (s), 30.04 (s), 21.45 (s).

*2,6-Di-tert-butyl-4-((2,4-dichlorophenyl)(tosyl)methyl)phenol* (**3at**): [15] Pale yellow solid; 68% yield. ^1^H-NMR (600 MHz, CDCl_3_) δ 8.29 (d, *J* = 8.5 Hz, 1H), 7.46 (d, *J* = 8.0 Hz, 2H), 7.37 (d, *J* = 8.5 Hz, 1H), 7.33 (s, 1H), 7.18 (d, *J* = 7.9 Hz, 2H), 7.10 (s, 2H), 5.79 (s, 1H), 5.25 (s, 1H), 2.39 (s, 1H), 1.35 (s, 18H). ^13^C-NMR (151 MHz, CDCl_3_) δ 154.31 (s), 144.50 (s), 136.06 (s), 135.56 (s), 135.47 (s), 134.79 (s), 130.90 (s), 130.83 (s), 129.62 (s), 129.29 (s), 128.99 (s), 127.44 (s), 127.08 (s), 122.18 (s), 70.63 (s), 34.29 (s), 30.09 (s), 21.53 (s).

*2,6-Di-tert-butyl-4-((1,3-dihydroisobenzofuran-4-yl)(tosyl)methyl)phenol* (**3au**): [15] Pale yellow solid; 68% yield. ^1^H-NMR (600 MHz, CDCl_3_) δ 7.43 (d, *J* = 8.1 Hz, 1H), 7.20 (s, 1H), 7.13 (d, *J* = 9.6 Hz, 1H), 7.01 (d, *J* = 8.1 Hz, 1H), 6.76 (d, *J* = 8.1 Hz, 1H), 5.95 (d, *J* = 3.4 Hz, 1H), 5.21 (s, 1H), 5.08 (s, 1H), 2.36 (s, 1H), 1.35 (s, 1H). ^13^C-NMR (151 MHz, CDCl_3_) δ 154.19 (s), 148.02 (s), 147.93 (s), 144.14 (s), 136.09 (s), 135.86 (s), 129.20 (s), 127.14 (s), 127.03 (s), 124.14 (s), 123.83 (s), 110.36 (s), 108.45 (s), 101.37 (s), 76.60 (s), 34.43 (s), 32.20–27.96 (m), 21.62 (s); HRMS calculated for [M + Na]^+^ C_29_H_34_O_5_SNa^+^, m/z 517.2019, found 517.2020.

*2,6-Di-tert-butyl-4-(naphthalen-1-yl(tosyl)methyl)phenol* (**3av**): [15] Pale yellow solid; 78% yield. ^1^H-NMR (600 MHz, CDCl_3_) δ 8.55 (d, *J* = 7.2 Hz, 1H), 7.86 (d, *J* = 7.8 Hz, 1H), 7.82 (t, *J* = 7.8 Hz, 2H), 7.59 (t, *J* = 7.7 Hz, 1H), 7.46 (d, *J* = 7.9 Hz, 2H), 7.45–7.39 (m, 2H), 7.25 (s, 1H), 7.14 (s, 1H), 7.12 (d, *J* = 7.9 Hz, 2H), 6.05 (s, 1H), 5.19 (s, 1H), 2.33 (s, 3H), 1.31 (s, 18H). ^13^C-NMR (151 MHz, CDCl_3_) δ 154.12 (s), 144.09 (s), 136.00 (s), 135.77 (s), 134.12 (s), 131.79 (s), 129.79 (s), 129.16 (s), 129.13 (s), 128.92 (s), 127.32 (s), 126.85 (s), 126.50 (s), 125.49 (s), 125.32 (s), 123.36 (s), 122.37 (s), 71.18 (s), 34.24 (s), 30.08 (s), 21.46 (s).

*2,6-Dimethyl-4-(phenyl(tosyl)methyl)phenol* (**3aw**): [15] Pale yellow solid; 63% yield. ^1^H-NMR (600 MHz, CDCl_3_) δ 7.54–7.44 (m, 4H), 7.28 (m, 3H), 7.15 (m, 4H), 5.13 (s, 1H), 4.70 (s, 1H), 2.37 (s, 3H), 2.20 (s, 6H). ^13^C-NMR (151 MHz, CDCl_3_) δ 152.62 (s), 144.22 (s), 135.65 (s), 133.77 (s), 130.25 (s), 129.86 (s), 129.15 (s), 129.08 (s), 128.56 (s), 128.35 (s), 124.34 (s), 123.28 (s), 76.09 (s), 21.53 (s), 15.90 (s).

*2,6-Diisopropyl-4-(phenyl(tosyl)methyl)phenol* (**3ax**): [15] Pale yellow solid; 79% yield. ^1^H-NMR (600 MHz, CDCl_3_) δ 7.59 (d, *J* = 7.8 Hz, 1H), 7.45 (d, *J* = 7.4 Hz, 1H), 7.37–7.28 (m, 1H), 7.13 (d, *J* = 8.0 Hz, 1H), 7.09 (s, 1H), 5.20 (s, 1H), 4.81 (s, 1H), 3.19–2.90 (m, 1H), 2.35 (s, 1H), 1.22 (d, *J* = 6.9 Hz, 1H), 1.14 (d, *J* = 6.8 Hz, 1H). ^13^C-NMR (151 MHz, CDCl_3_) δ 150.23 (s), 144.02 (s), 133.77 (s), 133.48 (s), 129.99 (s), 129.08 (s), 129.05 (s), 128.58 (s), 128.36 (s), 125.59 (s), 124.87 (s), 99.99 (s), 76.62 (s), 27.21 (s), 22.61 (s), 22.53 (s), 21.48 (s).

*2,6-Di-tert-butyl-4-(1-tosylethyl)phenol* (**3ay**): [15] Pale yellow solid; 80% yield. ^1^H-NMR (600 MHz,) δ 7.33 (d, *J* = 8.2 Hz, 2H), 7.15 (d, *J* = 8.2 Hz, 2H), 6.78 (s, 2H), 5.21 (s, 1H), 4.13 (q, *J* = 7.2 Hz, 1H), 2.38 (s, 3H), 1.77 (d, *J* = 7.2 Hz, 3H), 1.32 (s, 18H). ^13^C-NMR (151 MHz,) δ 154.13 (s), 143.98 (s), 135.75 (s), 134.32 (s), 129.31 (s), 129.04 (s), 126.21 (s), 124.39 (s), 66.53 (s), 34.23 (s), 30.13 (s), 21.50 (s), 13.45 (s).

*2,6-Di-tert-butyl-4-(phenyl(phenylsulfonyl)methyl)phenol* (**4aa**): [16] Pale yellow solid; 92% yield. ^1^H-NMR (600 MHz, CDCl_3_) δ 7.62 (d, *J* = 7.2 Hz, 2H), 7.57 (d, *J* = 7.8 Hz, 2H), 7.50 (t, *J* = 7.3 Hz, 1H), 7.34 (dd, *J* = 15.7, 8.5 Hz, 5H), 7.19 (s, 2H), 5.24 (s, 1H), 5.21 (s, 1H), 1.36 (s, 18H).^13^C-NMR (151 MHz, CDCl_3_) δ 154.15 (s), 138.65 (s), 135.97 (s), 133.45 (s), 133.13 (s), 130.00 (s), 129.08 (s), 128.67 (s), 128.47 (s), 128.44 (s), 127.10 (s), 123.29 (s), 34.34 (s), 30.18 (s).

*2,6-Di-tert-butyl-4-(((4-(tert-butyl)phenyl)sulfonyl)(phenyl)methyl)phenol* (**4ab**): [16] Pale yellow solid; 77% yield. ^1^H-NMR (600 MHz, CDCl_3_) δ 7.63 (d, *J* = 7.1 Hz, 2H), 7.45 (d, *J* = 8.4 Hz, 2H), 7.38–7.30 (m, 5H), 7.12 (s, 2H), 5.21 (s, 1H), 5.18 (s, 1H), 1.34 (s, 18H), 1.29 (s, 9H). ^13^C-NMR (151 MHz, CDCl_3_) δ 157.07 (s), 154.04 (s), 135.85 (s), 135.59 (s), 133.66 (s), 129.96 (s), 129.01 (s), 128.60 (s), 128.33 (s), 127.13 (s), 125.42 (s), 123.57 (s), 76.88 (s), 35.12 (s), 34.29 (s), 31.04 (s), 30.16 (s).

*2,6-Di-tert-butyl-4-(((4-methoxyphenyl)sulfonyl)(phenyl)methyl)phenol* (**4ac**): [16] Pale yellow solid; 96% yield. ^1^H-NMR (400 MHz, CDCl_3_) δ 7.61 (d, *J* = 7.3 Hz, 2H), 7.46 (d, *J* = 8.4 Hz, 2H), 7.39–7.28 (m, 3H), 7.18 (s, 2H), 6.80 (d, *J* = 8.4 Hz, 2H), 5.23 (s, 1H), 5.16 (s, 1H), 3.81 (s, 3H), 1.36 (s, 18H). ^13^C-NMR (101 MHz, CDCl_3_) δ 154.29 (s), 137.65 (s), 136.11 (s), 133.01 (s), 131.68 (s), 130.54 (s), 129.95 (s), 128.78 (s), 128.65 (s), 128.53 (s), 127.08 (s), 122.89 (s), 76.97 (s), 34.32 (s), 30.13 (s).

*N-(4-(((3,5-di-tert-butyl-4-hydroxyphenyl)(phenyl)methyl)sulfonyl)phenyl) acetamide* (**4ad**): Pale yellow solid; 79% yield. ^1^H-NMR (600 MHz, DMSO(d^6^)) δ 10.28 (s, 1H), 7.61 (dd, *J* = 11.6, 8.3 Hz, 4H), 7.46 (d, *J* = 8.7 Hz, 2H), 7.35 (t, *J* = 7.4 Hz, 2H), 7.30 (t, *J* = 7.2 Hz, 1H), 7.23 (s, 2H), 7.03 (s, 1H), 5.77 (s, 1H), 2.06 (s, 3H), 1.31 (s, 18H).^13^C-NMR (151 MHz, DMSO(d^6^)) δ 169.47 (s), 154.32 (s), 144.13 (s), 139.25 (s), 134.72 (s), 130.26 (s), 130.14 (s), 128.86 (s), 128.55 (s), 126.96 (s), 124.72 (s), 118.24 (s), 74.46 (s), 34.96 (s), 30.68 (s), 24.62 (s); HRMS (ESI): *m/z* calcd for C_29_H_35_NO_4_SNa [M + Na]^+^, 516.2178; found, 516.2179.

*2,6-Di-tert-butyl-4-(((4-chlorophenyl)sulfonyl)(phenyl)methyl)phenol* (**4ae**): [16] Pale yellow solid; 75% yield. ^1^H-NMR (600 MHz, DMSO(d^6^)) δ 7.64 (d, *J* = 7.3 Hz, 2H), 7.55 (dd, *J* = 22.6, 8.7 Hz, 4H), 7.39 (t, *J* = 7.4 Hz, 2H), 7.34 (t, *J* = 6.8 Hz, 1H), 7.21 (s, 2H), 7.08 (s, 1H), 5.95 (s, 1H), 1.31 (s, 18H).^13^C-NMR (151 MHz, DMSO(d^6^)) δ 154.47 (s), 139.40 (s), 138.99 (s), 137.71 (s), 134.04 (s), 130.83 (s), 130.31 (s), 129.18 (s), 129.00 (s), 128.78 (s), 127.05 (s), 124.18 (s), 74.08 (s), 34.95 (s), 30.63 (s).

*4-(((4-Bromophenyl)sulfonyl)(phenyl)methyl)-2,6-di-tert-butylphenol* (**4af**): [16] Pale yellow solid; 81% yield. ^1^H-NMR (400 MHz, CDCl_3_) δ 7.61 (dd, *J* = 7.7, 1.8 Hz, 2H), 7.50–7.45 (m, 2H), 7.42–7.40 (m, 1H), 7.40-7.32 (m, 4H), 7.17 (s, 2H), 5.26 (s, 1H), 5.17 (s, 1H), 1.37 (s, 18H). ^13^C-NMR (101 MHz, CDCl_3_) δ 154.29 (s), 137.65 (s), 136.11 (s), 133.01 (s), 131.68 (s), 130.54 (s), 129.95 (s), 128.78 (s), 128.65 (s), 128.53 (s), 127.08 (s), 122.89 (s), 76.97 (s), 34.32 (s), 30.13 (s).

*2,6-Di-tert-butyl-4-(((4-fluorophenyl)sulfonyl)(phenyl)methyl)phenol* (**4ag**): [16] Pale yellow solid; 62% yield. ^1^H-NMR (600 MHz, CDCl_3_) δ 7.60 (dd, *J* = 7.9, 1.2 Hz, 2H), 7.58–7.53 (m, 2H), 7.38–7.28 (m, 3H), 7.21 (s, 2H), 7.04–6.95 (m, 2H), 5.26 (s, 1H), 5.18 (s, 1H), 1.37 (s, 18H). ^13^C-NMR (101 MHz, CDCl_3_) δ 164.46 (d, *J* = 255.9 Hz), 153.21 (s), 135.05 (s), 133.62 (s), 132.21 (s), 130.79 (d, *J* = 9.5 Hz), 128.91 (s), 127.70 (s), 127.55 (s), 126.00 (s), 122.05 (s), 114.63 (d, *J* = 22.5 Hz), 75.99 (s), 33.30 (s), 29.12 (s).

*2,6-Di-tert-butyl-4-(phenyl((4-(trifluoromethyl)phenyl)sulfonyl)methyl)phenol* (**4ah**): [16] Pale yellow solid; 82% yield. ^1^H-NMR (600 MHz, CDCl_3_) δ 7.69 (d, *J* = 8.1 Hz, 2H), 7.65–7.57 (m, 4H), 7.40–7.33 (m, 3H), 7.17 (s, 2H), 5.27 (s, 1H), 5.21 (s, 1H), 1.35 (s, 18H). ^13^C-NMR (151 MHz, DMSO(d^6^)) δ 154.68 (s), 142.95 (s), 139.58 (s), 134.06–133.29 (m), 133.82 (s), 130.51 (s), 130.13 (s), 129.22 (s), 129.06 (s), 127.28 (s), 126.44–126.15 (m), 123.96 (q, *J* = 273.0 Hz), 123.98 (s), 74.06 (s), 35.07 (s), 30.72 (s).

*2,6-Di-tert-butyl-4-(phenyl((4-(trifluoromethoxy)phenyl)sulfonyl)methyl)phenol* (**4ai**): Pale yellow solid; 84% yield. ^1^H-NMR (600 MHz, CDCl_3_) δ 7.61 (d, *J* = 6.8 Hz, 1H), 7.48 (d, *J* = 8.5 Hz, 1H), 7.37–7.32 (m, 1H), 7.31 (d, *J* = 8.5 Hz, 1H), 7.18 (s, 1H), 5.25 (s, 1H), 5.17 (s, 1H), 1.37 (s, 1H). ^13^C-NMR (151 MHz, DMSO(d^6^)) δ 154.48 (s), 152.04 (d, *J* = 2.0 Hz), 139.39 (s), 137.73 (s), 134.00 (s), 131.65 (s), 130.32 (s), 129.00 (s), 128.80 (s), 127.04 (s), 124.13 (s), 121.18 (s), 120.26 (q, *J* = 258.2 Hz).74.07 (s), 34.93 (s), 30.59 (s); HRMS (ESI): *m/z* calcd for C_28_H_31_F_3_O_4_SNa [M + Na]^+^, 543.1785; found, 543.1787.

*2,6-Di-tert-butyl-4-(((3-nitrophenyl)sulfonyl)(phenyl)methyl)phenol* (**4aj**): Pale yellow solid; 86% yield. ^1^H-NMR (600 MHz, CDCl_3_) δ 8.38–8.32 (m, 1H), 8.30 (t, *J* = 1.7 Hz, 1H), 7.94 (d, *J* = 7.7 Hz, 1H), 7.63 (d, *J* = 6.6 Hz, 2H), 7.58 (t, *J* = 8.0 Hz, 1H), 7.42–7.33 (m, 3H), 7.23 (s, 2H), 5.30 (s, 1H), 5.24 (s, 1H), 1.36 (s, 18H). ^13^C-NMR (151 MHz, CDCl_3_) δ 154.56 (s), 147.81 (s), 140.94 (s), 136.49 (s), 134.26 (s), 132.33 (s), 129.99 (s), 129.66 (s), 128.97 (s), 128.93 (s), 127.49 (s), 127.07 (s), 124.48 (s), 122.32 (s), 77.12 (s), 34.35 (s), 30.14 (s); HRMS (ESI): *m/z* calcd for C_27_H_31_NO_5_SNa [M + Na]^+^, 504.1817; found, 504.1815.

*2,6-Di-tert-butyl-4-((naphthalen-1-ylsulfonyl)(phenyl)methyl)phenol* (**4ak**): [16] Pale yellow solid; 52% yield. ^1^H-NMR (400 MHz, CDCl_3_) δ 8.07 (d, *J* = 1.2 Hz, 1H), 7.85 (d, *J* = 8.2 Hz, 1H), 7.79 (dd, *J* = 8.1, 5.2 Hz, 2H), 7.67 (dd, *J* = 7.8, 1.6 Hz, 2H), 7.64–7.51 (m, 3H), 7.39–7.30 (m, 3H), 7.16 (s, 2H), 5.28 (s, 1H), 5.18 (s, 1H), 1.26 (s, 18H). ^13^C-NMR (101 MHz, CDCl_3_) δ 153.06 (s), 134.88 (s), 134.37 (s), 133.97 (s), 132.31 (s), 130.81 (s), 130.02 (s), 129.01 (s), 128.23 (s), 128.02 (s), 127.67 (s), 127.47 (s), 127.42 (s), 126.74 (s), 126.34 (s), 126.04 (s), 122.65 (s), 122.38 (s), 75.89 (s), 33.18 (s), 29.01 (s).

*2,6-Di-tert-butyl-4-(phenyl(thiophen-2-ylsulfonyl)methyl)phenol* (**4al**): [16] Pale yellow solid; 76% yield. ^1^H-NMR (600 MHz, CDCl_3_) δ 7.63 (d, *J* = 7.0 Hz, 2H), 7.54 (d, *J* = 3.7 Hz, 1H), 7.40–7.30 (m, 3H), 7.28 (s, 3H), 7.01–6.89 (m, 1H), 5.30 (s, 1H), 5.26 (s, 1H), 1.38 (s, 18H). ^13^C-NMR (151 MHz, DMSO(d^6^)) δ 154.54 (s), 139.54 (s), 139.40 (s), 136.07 (s), 135.32 (s), 134.54 (s), 130.23 (s), 128.97 (s), 128.78 (s), 128.05 (s), 126.89 (s), 124.58 (s), 75.79 (s), 35.02 (s), 30.71 (s).

*4-((Benzylsulfonyl)(phenyl)methyl)-2,6-di-tert-butylphenol* (**4am**): [16] Pale yellow solid; 84% yield. ^1^H-NMR (600 MHz, CDCl_3_) δ 7.57 (dd, *J* = 5.2, 3.3 Hz, 2H), 7.42–7.29 (m, 8H), 7.17 (dd, *J* = 7.9, 1.3 Hz, 2H), 5.31 (s, 1H), 5.03 (s, 1H), 4.33–3.57 (m, 2H), 1.44 (s, 18H). ^13^C-NMR (101 MHz, CDCl_3_) δ 154.38 (s), 136.32 (s), 132.83 (s), 130.97 (s), 130.11 (s), 128.82 (s), 128.78 (s), 128.68 (d, *J* = 2.0 Hz), 128.07 (s), 126.88 (s), 122.93 (s), 71.81 (s), 58.13 (s), 34.48 (s), 30.30 (s).

#### 3.2.2. Representative Procedure for General Reaction Procedure for Synthesis of Unsymmetrical Triarylmethane **7**

To a solution of 2,6-di-*tert*-butyl-4-(phenyl(tosyl)methyl) phenol (45 mg, 0.10 mmol) in 1 mL DCE, KOH (5.6 mg, 0.10 mmol) and indole (14.06 mg, 0.12 mmol) were added. The mixture was stirred at 65 °C. The reaction was monitored by TLC. After complete reaction, the mixture was washed with water, dried over by anhydrous magnesium sulfate, and concentrated in vacuo. The crude product was then purified by flash column chromatography on silica gel to afford the pure product **7**.

*2,6-Di-tert-butyl-4-((3a,7a-dihydro-1H-indol-3-yl)(phenyl)methyl)phenol* (**7**): [41] Brown solid; 85% yield. ^1^H-NMR (600 MHz, CDCl_3_) δ 7.90 (s, 1H), 7.33 (d, *J* = 8.1 Hz, 1H), 7.25 (d, *J* = 5.3 Hz, 4H), 7.22 (d, *J* = 8.1 Hz, 1H), 7.18 (qd, *J* = 5.3, 2.8 Hz, 1H), 7.14 (t, *J* = 7.5 Hz, 1H), 7.04 (s, 2H), 6.97 (t, *J* = 7.5 Hz, 1H), 6.58 (d, *J* = 23.9 Hz, 1H), 5.56 (s, 1H), 5.04 (s, 1H), 1.36 (s, 18H).^13^C-NMR (151 MHz, CDCl_3_) δ 152.91 (d, *J* = 2.9 Hz), 141.67 (s), 136.43 (d, *J* = 2.7 Hz), 135.80 (d, *J* = 2.2 Hz), 131.54 (s), 131.31 (s), 128.61 (d, *J* = 2.9 Hz), 128.43 (s), 127.04 (d, *J* = 2.6 Hz), 126.61 (d, *J* = 3.0 Hz), 125.21 (d, *J* = 2.8 Hz), 58.10 (s), 34.43 (s), 30.33 (s).

#### 3.2.3. Representative Procedure for General Reaction Procedure for Synthesis of Unsymmetrical Gem-Diarylmethyl Thioether **8**

To a solution of 2,6-di-*tert*-butyl-4-(phenyl(tosyl)methyl) phenol (45 mg, 0.1 mmol) in 1 mL methyl *tert*-butyl ether, Cs_2_CO_3_ (36 mg, 0.01 mmol) and TBAB (3.6 mg, 0.01 mmol) were added. Benzenethiol (13.68 μL, 0.12 mmol) was added to the solution dropwise, and the mixture was stirred at 80 °C. The reaction was monitored by TLC. After complete reaction, the mixture was concentrated in vacuo. The crude product was then purified by flash column chromatography on silica gel to afford the pure product **8**.

*2,6-Di-tert-butyl-4-(phenyl(phenylthio)methyl)phenol* (**8**): [42] Colorless gummy liquid; 76% yield. ^1^H-NMR (600 MHz, CDCl3) δ 7.38 (d, *J* = 7.4 Hz, 2H), 7.20 (t, *J* = 7.3 Hz, 2H), 7.12 (t, *J* = 7.0 Hz, 3H), 7.10–7.00 (m, 5H), 5.35 (d, *J* = 23.3 Hz, 1H), 5.01 (d, *J* = 23.1 Hz, 1H), 1.29 (s, 18H). ^13^C-NMR (151 MHz, CDCl3) δ 152.00 (s), 144.65 (s), 136.75 (s), 135.42 (s), 135.29 (s), 134.46 (s), 128.95 (s), 128.09 (s), 127.19 (s), 125.92 (s), 125.54 (s), 123.80 (s), 121.92 (s), 120.11 (s), 119.23 (s), 110.89 (s), 48.84 (s), 34.35 (s), 30.40 (s).

## 4. Conclusions

In summary, we developed the TBAB-promoted sulfa-1,6-conjugated addition of *para*-quinone methides with sulfonyl hydrazines in water. This reaction provides a green and sustainable method for direct synthesis of various unsymmetrical diarylmethyl sulfones with good functional group tolerance, scalability, and regioselectivity. Further transformation of the resulting diarylmethyl sulfones provides an efficient route to some functionalized molecules.

## Figures and Tables

**Table 1:**
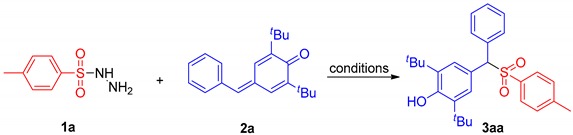
Optimization of the reaction conditions for the 1,6-conjugate sulfonylation of 4-methylbenzenesulfonohydrazide **1a** to *p*-QM **2a**^a^.

Entry	Catalyst	Solvent	*T* (°C)	Time (h)	Yield ^b^ (%)
1	-	Et_2_O	40	10	0
2	-	Toluene	80	10	0
3	-	THF	70	10	50
4	-	DCE	80	10	trace
5	-	Dioxane	80	10	trace
6	-	DMSO	80	10	trace
7	-	H_2_O	80	10	61
8	-	EtOH	80	10	73
9	Pd/C	H_2_O	80	10	75
10	Pd/TiO_2_	H_2_O	80	10	53
11	Au/TiO_2_	H_2_O	80	10	43
12	Pt/C	H_2_O	80	10	70
13	CTAB	H_2_O	80	10	51
14	TBAI	H_2_O	80	10	78
15	TEBAC	H_2_O	80	10	80
16	TBAB	H_2_O	80	10	82
17	SDS	H_2_O	80	10	18
18	TBAB	H_2_O	90	10	81
19	TBAB	H_2_O	60	10	63
20	TBAB	H_2_O	30	10	15
21	TBAB	H_2_O	80	12	90
22 **^c^**	TBAB	H_2_O	80	12	96

^a^ The reaction conditions: **1a** (0.20 mmol), **2a** (0.20 mmol), catalyst (0.02 mmol), solvent (1 mL), ^b^ isolated yield based on **1a**, ^c^ **2a** (0.3 mmol).

## Supplementary Materials

The following are available online, the ^1^H-NMR, ^13^C-NMR spectra of compounds **3aa**–**3ay** and **4aa**–**4am**, **7**, **8**; HRMS data of **4ad**, **4ai** and **4aj**; crystal data of **4af** are available as supporting data.

## Abbreviations

THF	Tetrahydrofuran
DCE	Dichloroethane
DMSO	Dimethylsulfoxide
CTAB	Cetyltrimethyl Ammonium Bromide
TBAI	Tetrabutyl Ammonium Iodide
TEBAC	Triethylbenzyl Ammonium Chloride
TBAB	Tetrabutyl Ammonium Bromide
SDS	Sodium Dodecyl Sulfate
HRMS	High Resolution Mass Spectroscopy
