# Supplementary material for: TBAB-Catalyzed 1,6-Conjugate Sulfonylation of para-Quinone Methides: A Highly Efficient Approach to Unsymmetrical gem-Diarylmethyl Sulfones in Water
Source: Molecules. 2020 Jan 26;25(3):539. doi: 10.3390/molecules25030539 (PMC7038064; doi:10.3390/molecules25030539)
Supplement: Supplementary file 1 [file molecules-25-00539-s001.pdf]

1. General methods

^{1}H NMR, ^{13}C NMR spectra were obtained utilizing a Bruker 600 and 400 MHz instrument and reported in $CDCl_3$ or DMSO(d^6). ^{1}H and ^{13}CNMR chemical shifts are reported in ppm relative to either TMS (^{1}H) ($\delta = 0$ ppm) as an internal standard or the residual solvent peak as following: $CDCl_3$ =7.26 (^{1}H NMR), $(CD_3)_2SO$ = 2.50 (^{1}H NMR), $CDCl_3$ = 77.16 (^{13}C NMR), $(CD_3)_2SO$ = 40.00 (^{13}C NMR). Commercially available chemicals and solvents were purchased from Adamas-beta, Energy Chemical, Chongqing Chuan dong Chemical and Chengdu Kelong Chemical. The corresponding compounds were synthesized according to the reported literature methods. Analytical thin-layer chromatography (TLC) was performed on silica gel plates with F-254 indicator and compounds were visualized by irradiation with UV light. Chromatography was carried out using silica gel 300-400 mesh. HRMs were performed on Bruker Impact II 10200 instrument.

2. Experimental Procedures

2.1 General procedure for synthesis of *p*-QMs 2[1]

2.1.1 General procedure for synthesis of 2,6-di-*tert*-butyl-4-substituted benzylidene cyclohexa-2,5-dien-1-one (2a-2v)

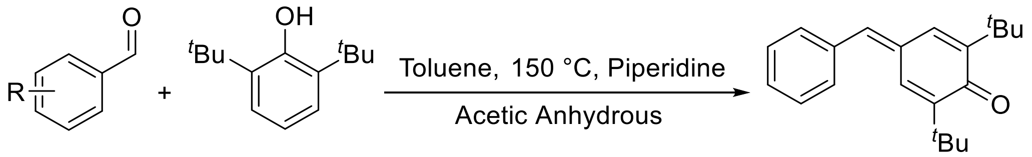

In a Dean-Stark apparatus, a solution of 2,6-di-*tert*-butylphenol (1.0 mmol) in 5 mL toluene, the corresponding aromatic aldehyde (1.0 mmol) and piperidine (2.0 mmol) were added. The reaction mixture was stirred at 150 ℃. The reaction was monitored by TLC. After complete reaction, the mixture was cooled just below the boiling point of the reaction mixture, and acetic anhydride (2.0 mmol) was added drop wisely in the continuously stirring. The mixture was washed with water, extracted with ethyl acetic, dried over anhydrous magnesium sulfate and concentrated in vacuo. The crude

produce was then purified by flash column chromatography on silica gel (gradient eluent of EA/PE=1/200-1/100) to gain corresponding product.

2.1.2 General procedure for synthesis of 4-benzylidene-2,6-dimethylcyclohexa-2,5-dien-1-one (2w)[2]

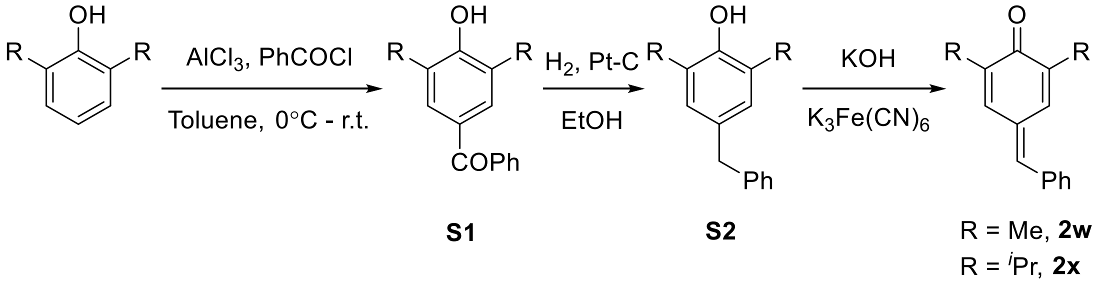

2aw and **2ax** was synthesized according to the following procedure: benzoyl chloride (1.80 equiv.) was added dropwise to the corresponding benzaldehyd e (1.0 equiv.) in toluene. Then aluminium chloride (0.40 equiv.) was slowly added and the mixture was stirred at room temperature for 7 h. After completion, solvents were evaporated and directly subjected to flash chromatography to obtain the pure product **S1**. Palladium (10% by weight on carbon powder) (5% equiv.) was added in one portion to a solution of **S1** (1.0 equiv.) in ethanol under an atmosphere of H_2 (balloon). The reaction mixture was stirred vigorously for 3 h at room temperature and then filtered through a pad of celite. The solvent was removed under reduced pressure and the residue was purified by silica gel flash column chromatography to afford **S2**. Potassium ferricyanide (4.0 equiv.) and potassium hydroxide (4.2 equiv.) in water were added in one portion to a solution of **S2** (1.0 equiv.) in hexane under argon. The reaction mixture was stirred vigorously for 1 h at 23 ℃. The aqueous layer was separated and extracted with hexanes. The combined organic layers were washed with brine and dried over anhydrous sodium sulfate. The solids were removed by filtration and the solution was concentrated by rotary evaporation. The residue was purified by silica gel flash column chromatography to afford corresponding product **2w** and **2x**.

2.1.3 General procedure for synthesis of 2,6-di-tert-butyl-4-ethylidenecyclohexa-2,5-dien-1-one[2]

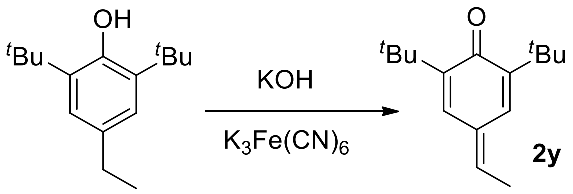

Potassium ferricyanide (4.0 equiv.) and potassium hydroxide (4.2 equiv.) in water were added in one portion to a solution of 2,6-di-*tert*-butyl-4-ethylphenol (1.0 equiv.) in hexane under argon. The reaction mixture was stirred vigorously for 1h at 23 ℃. The aqueous layer was separated and extracted with hexanes. The combined organic layers were washed with brine and dried over anhydrous sodium sulfate. The solids were removed by filtration and the solution was concentrated by rotary evaporation. The residue was purified by silica gel flash column chromatography to afford corresponding product **2y**

2.2 General procedure for synthesis of sulfonyl hydrazides[3]

Hydrazine hydrate (12.5 mmol) was added dropwise to a solution of sulfonyl chloride (5.0 mmol) in dry THF (25 mL) at 0 ℃ under N_2. After vigorous stirring for 30 min at 0 ℃, ethyl acetate (60 mL) was added, and the mixture was washed repeatedly with ice-cold 10% aqueous sodium chloride solution (3 × 20 mL). The organic layer was dried over sodium sulfate, filtered, and added slowly to stirred hexane (40 mL) over 5 min. Sulfonyl hydrazide precipitated within 10 min as an off-white solid and was collected by vacuum filtration. The filter cake was washed with hexanes (2 × 50 mL) and then was dried in vacuum to give corresponding sulfonyl hydrazides in 60-80% yields.

3. References

[1] Zhang, X. Z.; Du, J. Y.; Deng, Y. H.; Chu, W. D.; Yan, X.; Yu, K. Y.; Fan, C. A. *J. Org. Chem.* **2016**, *81*, 2598

[2] Gao, S.; Xu, X.; Yuan, Z.; Zhou, H.; Yao, H.; Lin, A. *Eur. J. Org. Chem.* **2016,** 3006.

[3] L. Liu, K. Sun, L. Su, J. Dong, L. Cheng, X. Zhu, C. T. Au, Y. Zhou, S. F. Yin, *Org. Lett* **2018**, *20*, 4023-4027.

4. NMR spectra

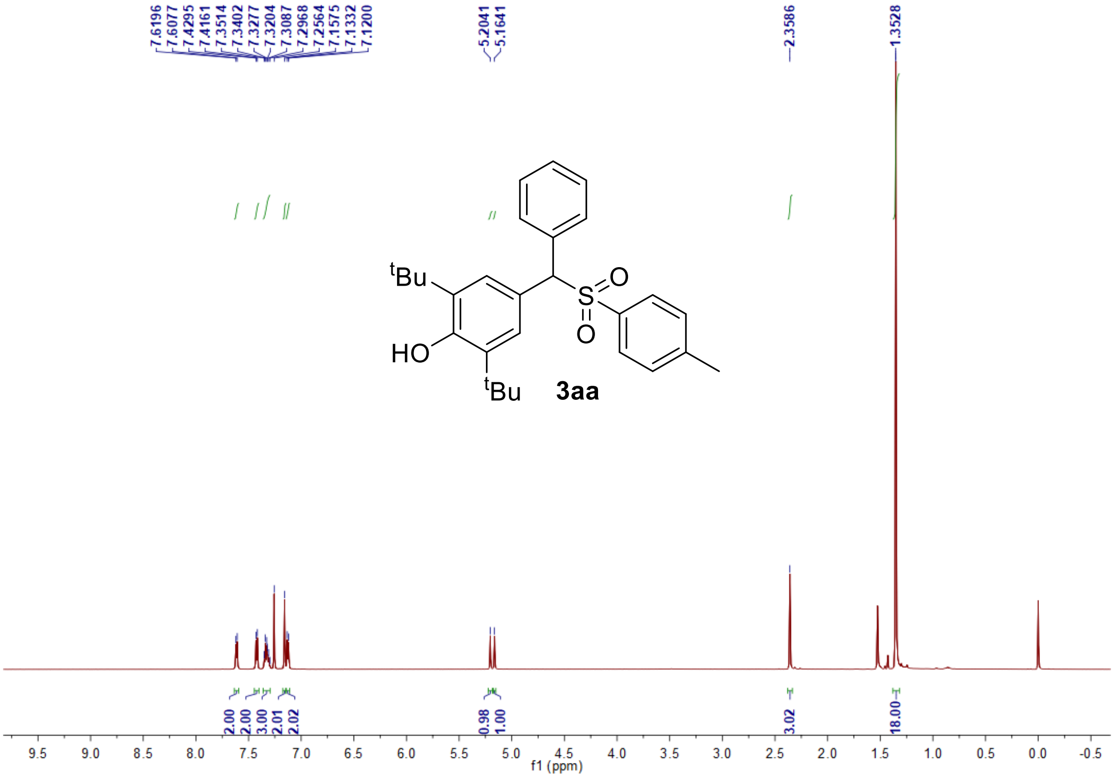

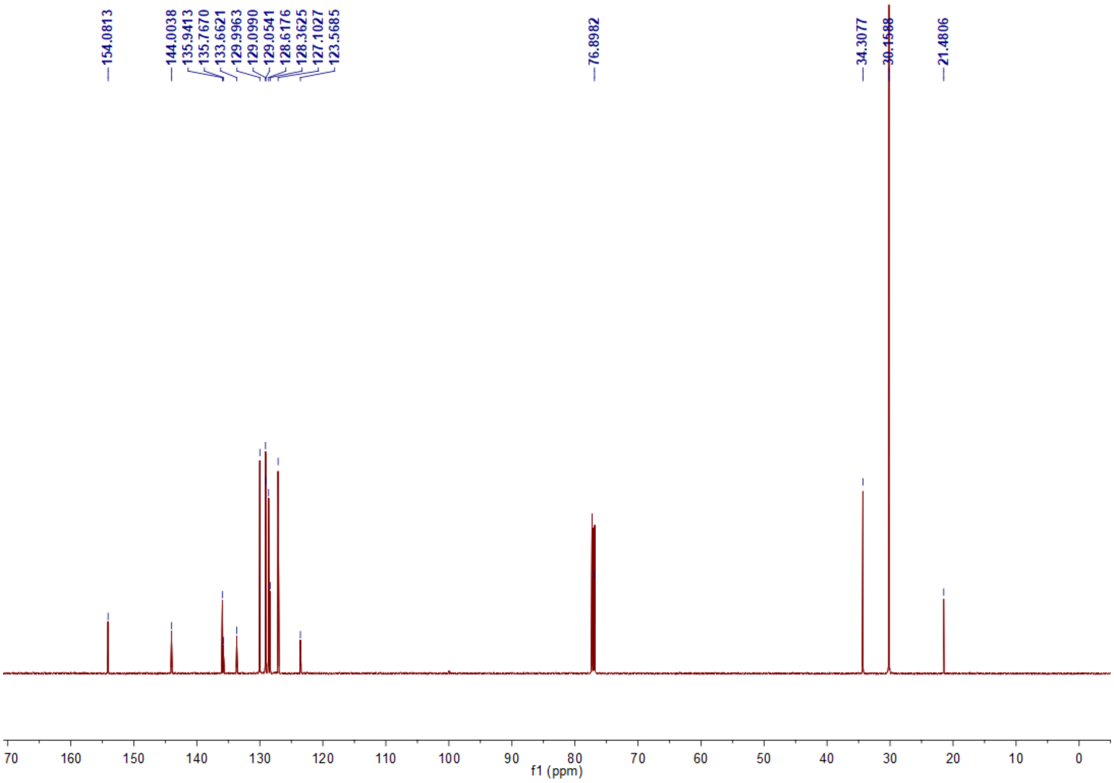

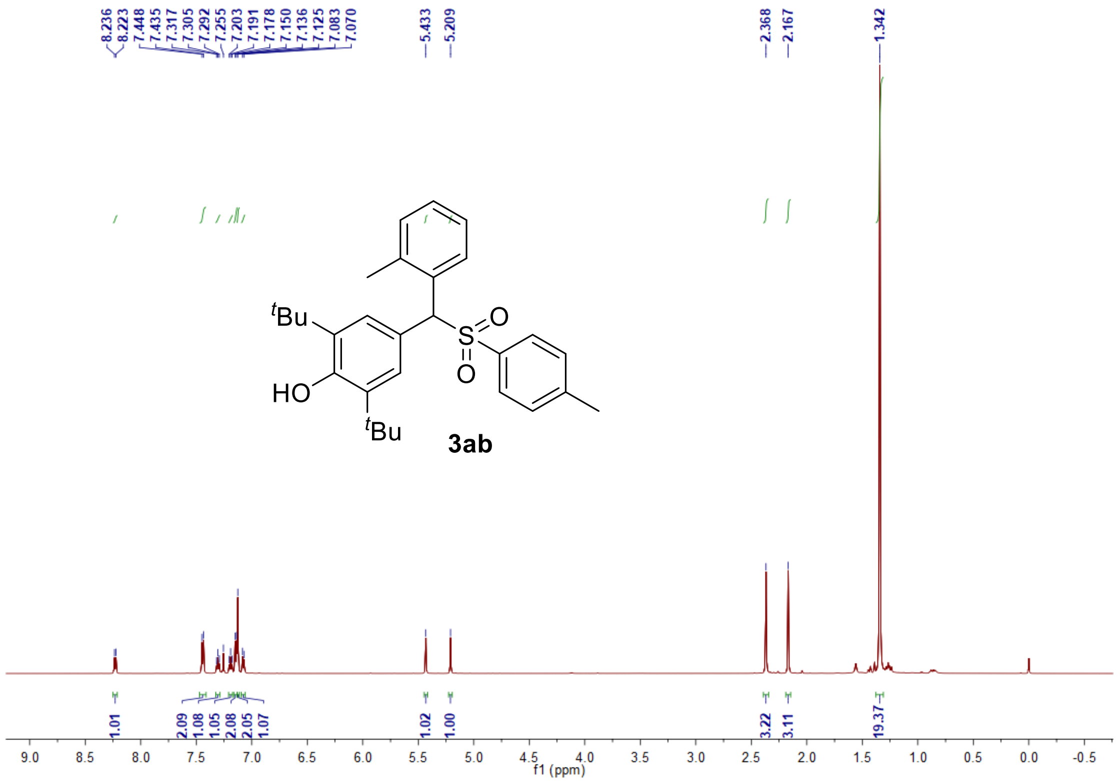
8.236
8.223
7.448
7.435
7.317
7.305
7.292
7.255
7.203
7.191
7.178
7.150
7.136
7.125
7.083
7.070
5.433
5.209
2.368
2.167
1.342
tBu
HO
tBu
S
O
O
3ab
1.01
2.09
1.08
1.05
2.08
2.05
1.07
1.02
1.00
3.22
3.11
19.37
f1 (ppm)

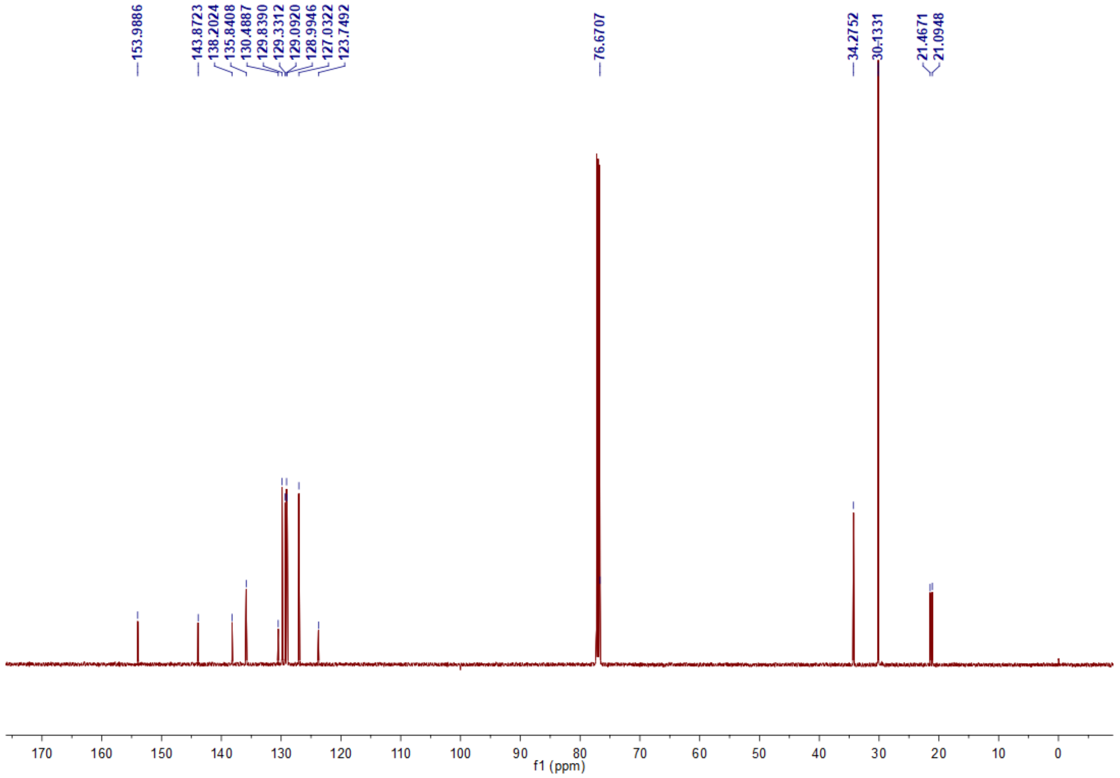
153.9886
143.8723
138.2024
135.8408
130.4887
129.8390
129.3312
129.0920
128.9946
127.0322
123.7492
76.6707
34.2752
30.1331
21.4671
21.0948
f1 (ppm)

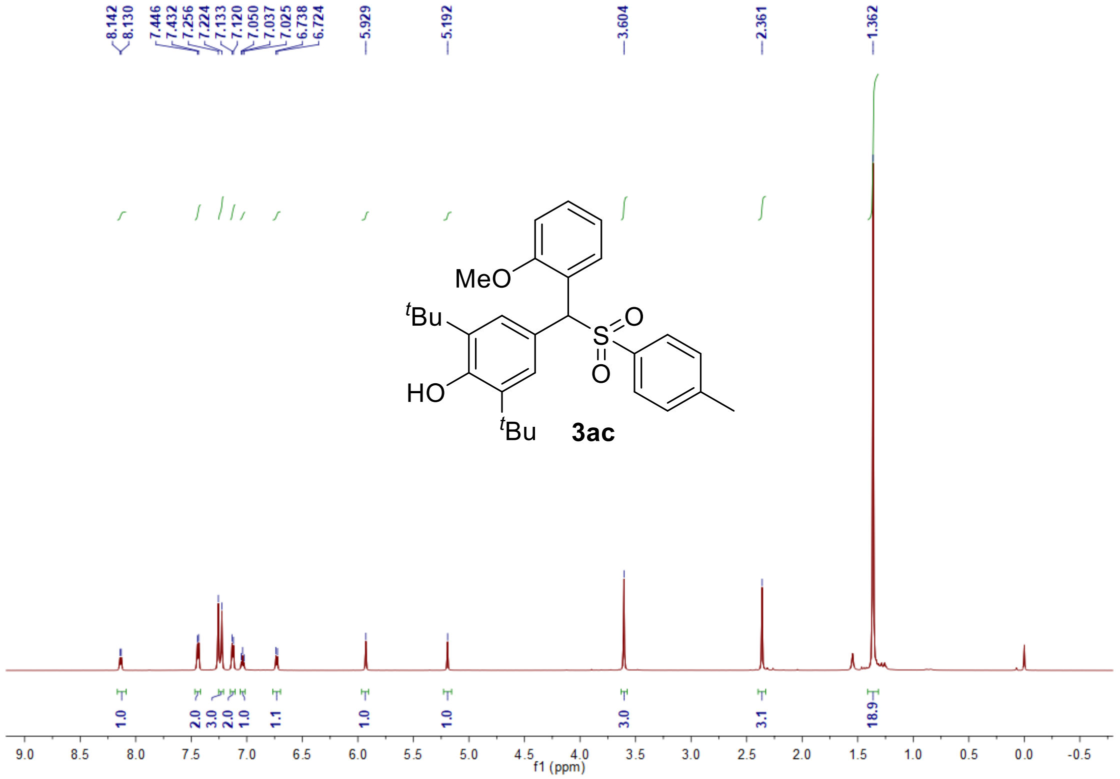
8.142
8.130
7.446
7.432
7.256
7.224
7.133
7.120
7.050
7.037
7.025
6.738
6.724
5.929
5.192
3.604
2.361
1.362
MeO
^{t}Bu
S
O
O
HO
^{t}Bu
3ac
1.0
2.0
3.0
2.0
1.0
1.1
1.0
1.0
3.0
3.1
18.9
9.0 8.5 8.0 7.5 7.0 6.5 6.0 5.5 5.0 4.5 4.0 3.5 3.0 2.5 2.0 1.5 1.0 0.5 0.0 -0.5
f1 (ppm)

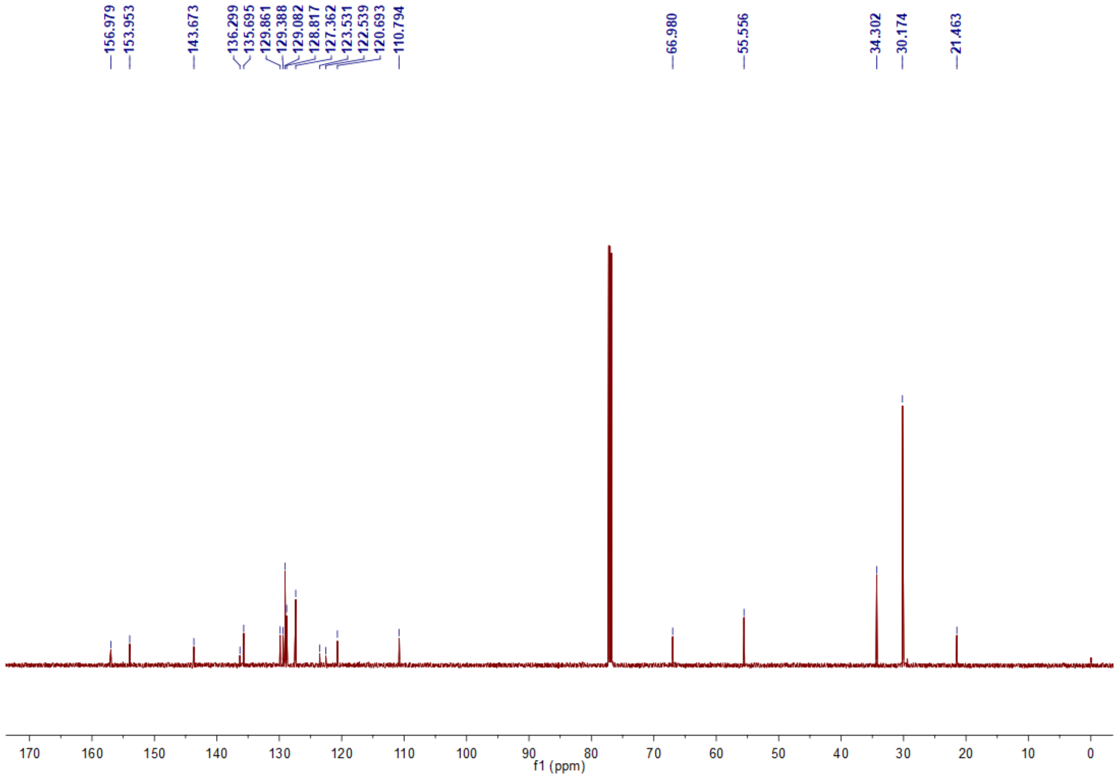
156.979
153.953
143.673
136.299
135.695
129.861
129.388
129.082
128.817
127.362
123.531
122.539
120.693
110.794
66.980
55.556
34.302
30.174
21.463
170 160 150 140 130 120 110 100 90 80 70 60 50 40 30 20 10 0
f1 (ppm)

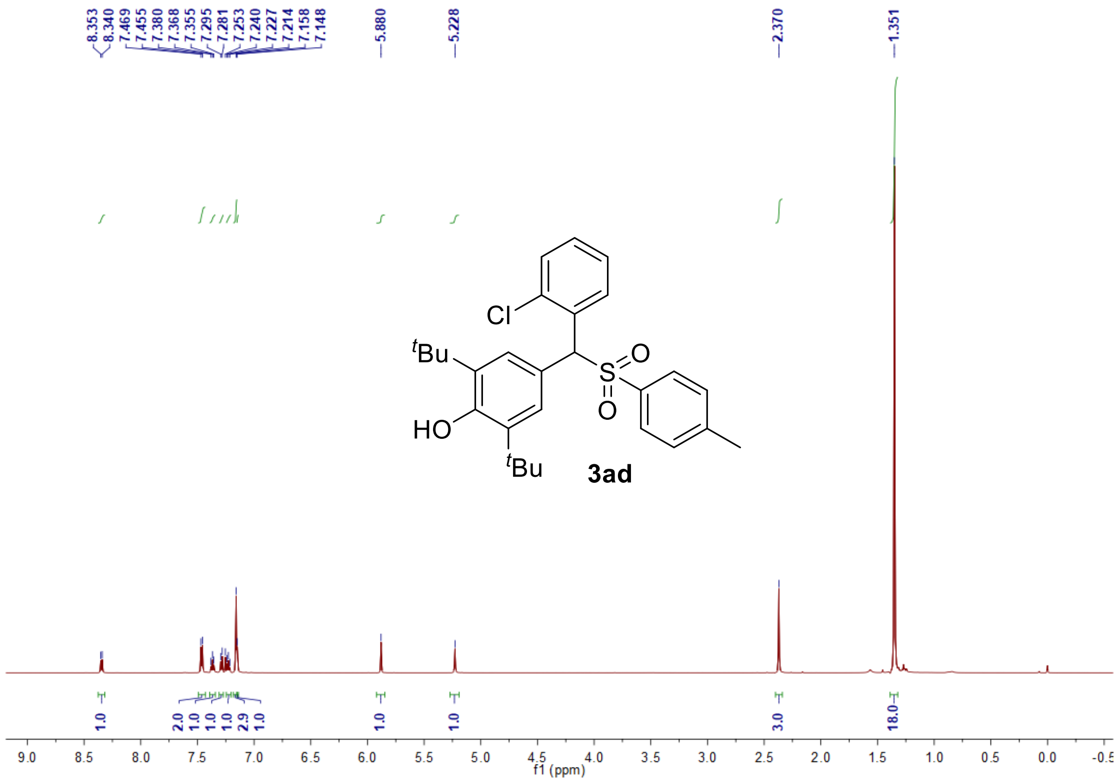
8.353
8.340
7.469
7.455
7.380
7.368
7.355
7.295
7.281
7.253
7.240
7.227
7.214
7.158
7.148
5.880
5.228
2.370
1.351
Cl
tBu
HO
tBu
S
O
O
3ad
1.0
2.0
1.0
1.0
1.0
2.9
1.0
1.0
1.0
3.0
18.0
f1 (ppm)

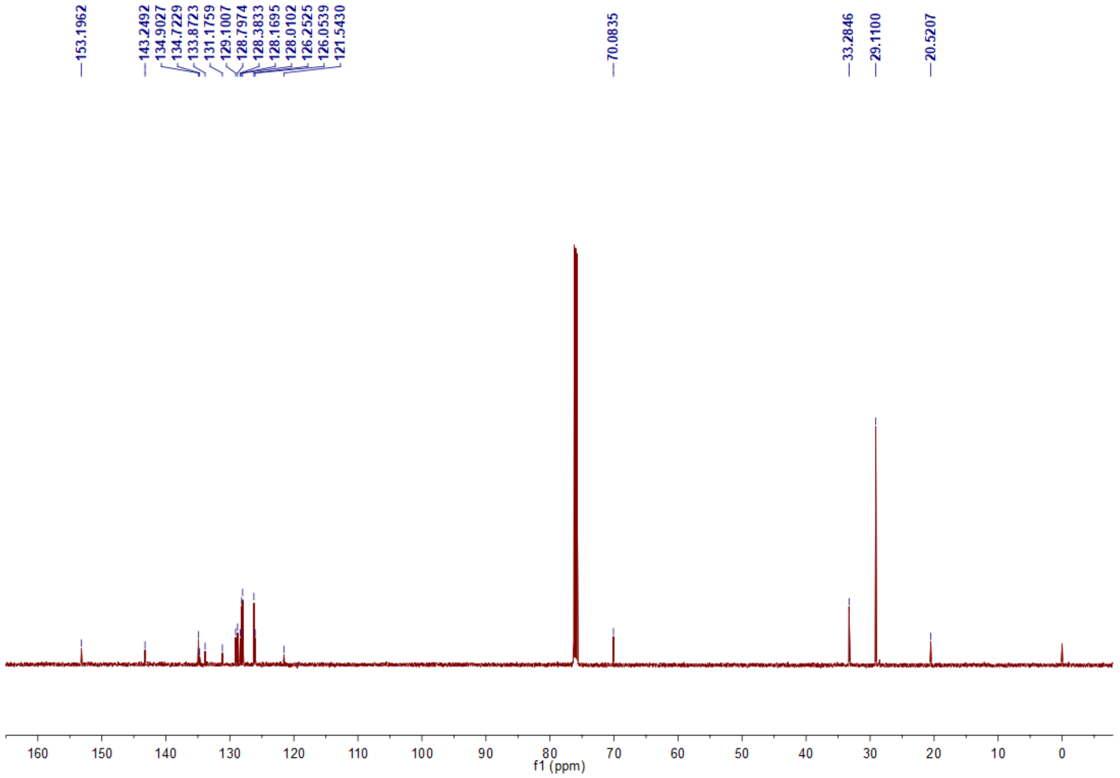
153.1962
143.2492
134.9027
134.7229
133.8723
131.1759
129.1007
128.7974
128.3833
128.1695
128.0102
126.2525
126.0539
121.5430
70.0835
33.2846
29.1100
20.5207
f1 (ppm)

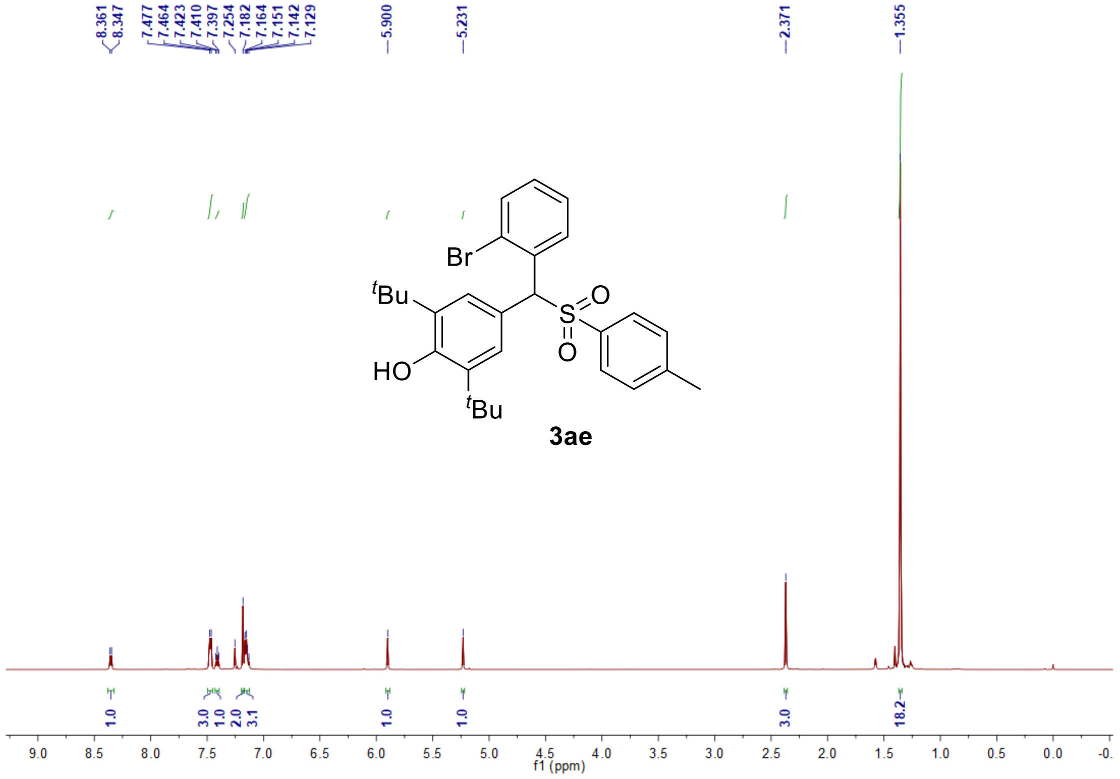
8.361
8.347
7.477
7.464
7.423
7.410
7.397
7.254
7.182
7.164
7.151
7.142
7.129
5.900
5.231
2.371
1.355
Br
tBu
HO
tBu
S
O
O
3ae
1.0
3.0
1.0
2.0
3.1
1.0
1.0
3.0
18.2
f1 (ppm)

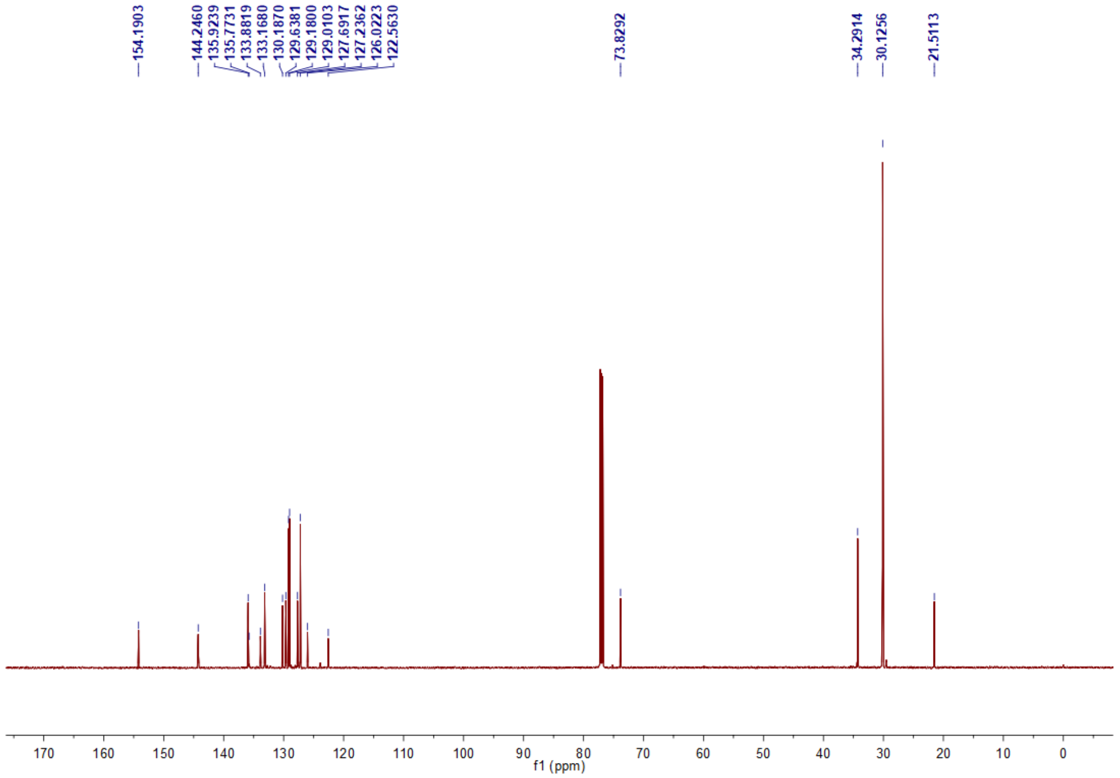
154.1903
144.2460
135.9239
135.7731
133.8819
133.1680
130.1870
129.6381
129.1800
129.0103
127.6917
127.2362
126.0223
122.5630
73.8292
34.2914
30.1256
21.5113
f1 (ppm)

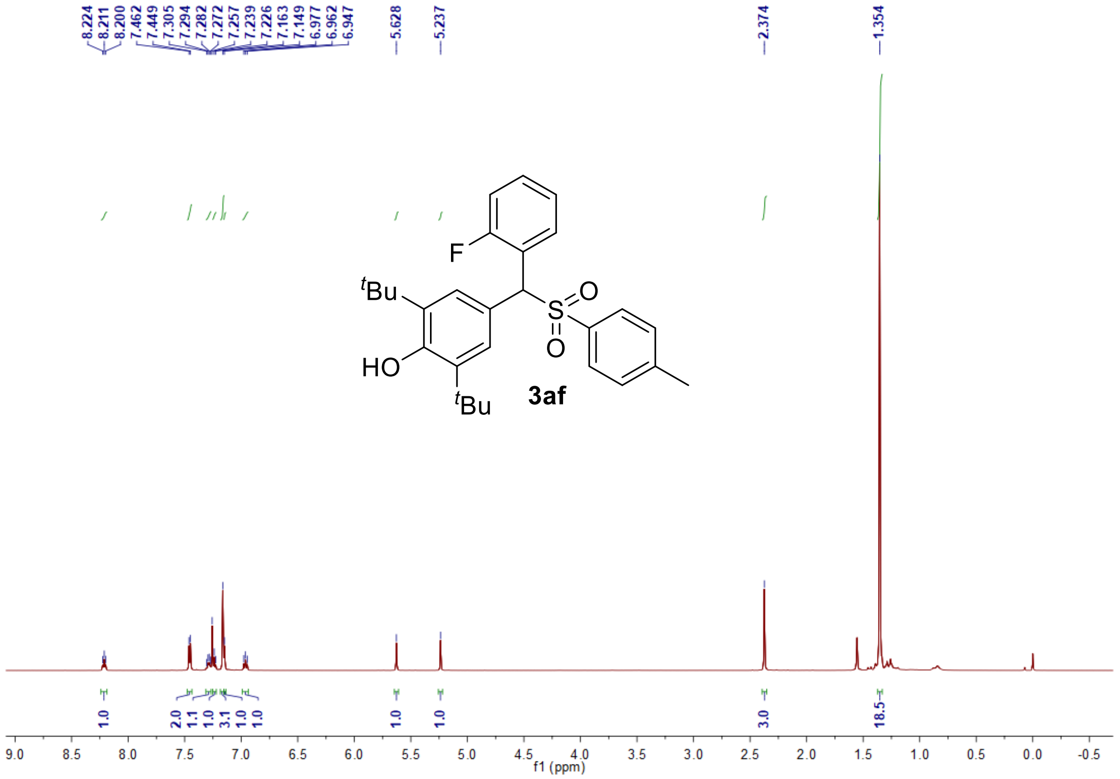
8.224
8.211
8.200
7.462
7.449
7.305
7.294
7.282
7.272
7.257
7.239
7.226
7.163
7.149
6.977
6.962
6.947
5.628
5.237
2.374
1.354
F
tBu
HO
tBu
S
O
O
3af
1.0
2.0
1.1
1.0
3.1
1.0
1.0
1.0
1.0
3.0
18.5
f1 (ppm)

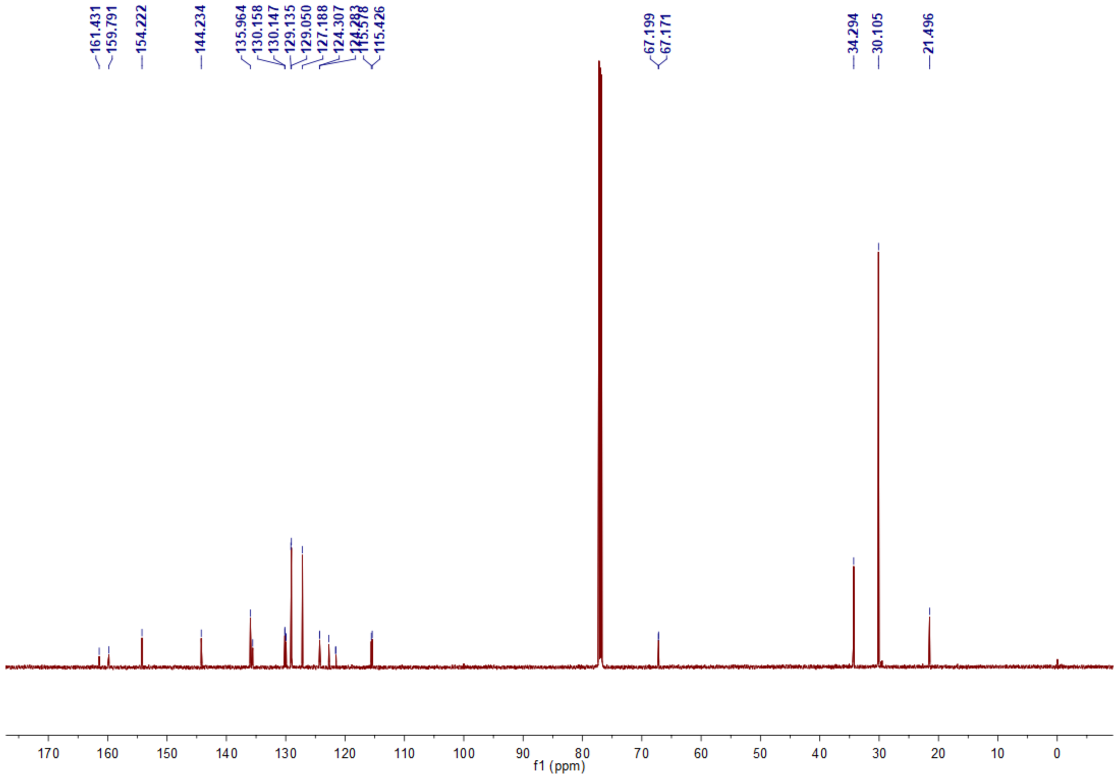
161.431
159.791
154.222
144.234
135.964
130.158
130.147
129.135
129.050
127.188
124.307
67.199
67.171
34.294
30.105
21.496
f1 (ppm)

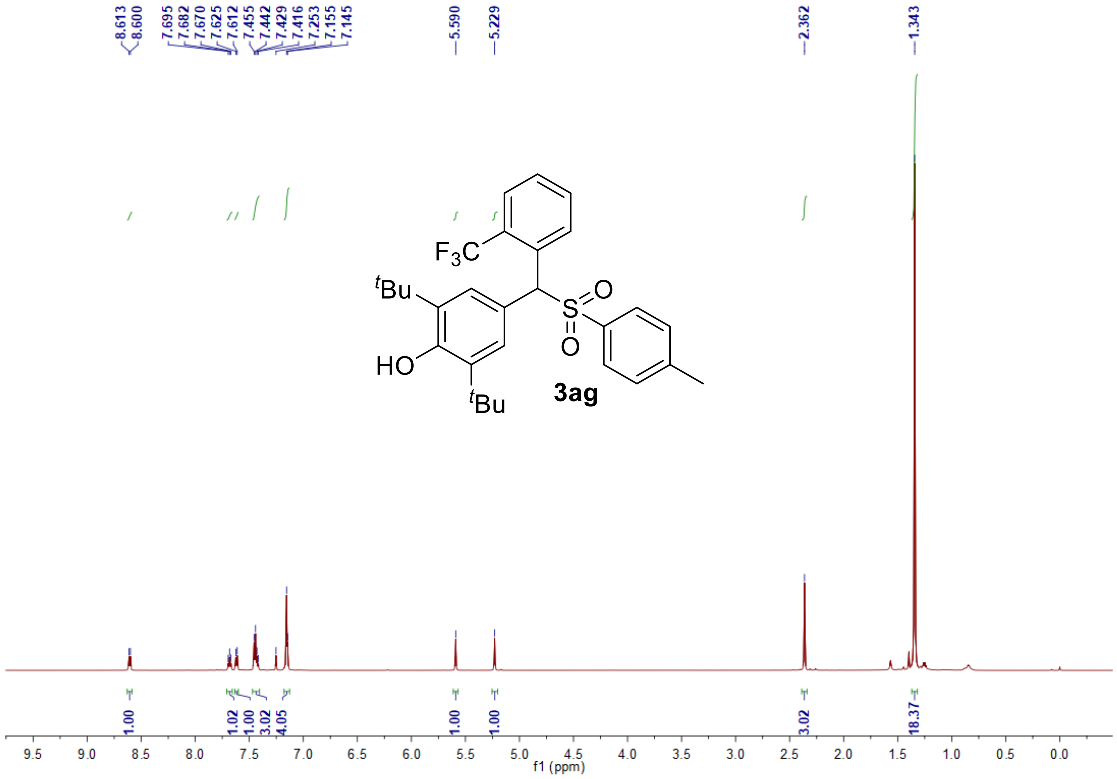
8.613
8.600
7.695
7.682
7.670
7.625
7.612
7.455
7.442
7.429
7.416
7.253
7.155
7.145
5.590
5.229
2.362
1.343
F3C
tBu
HO
tBu
3ag
1.00
1.02
1.00
3.02
4.05
1.00
1.00
3.02
18.37
f1 (ppm)

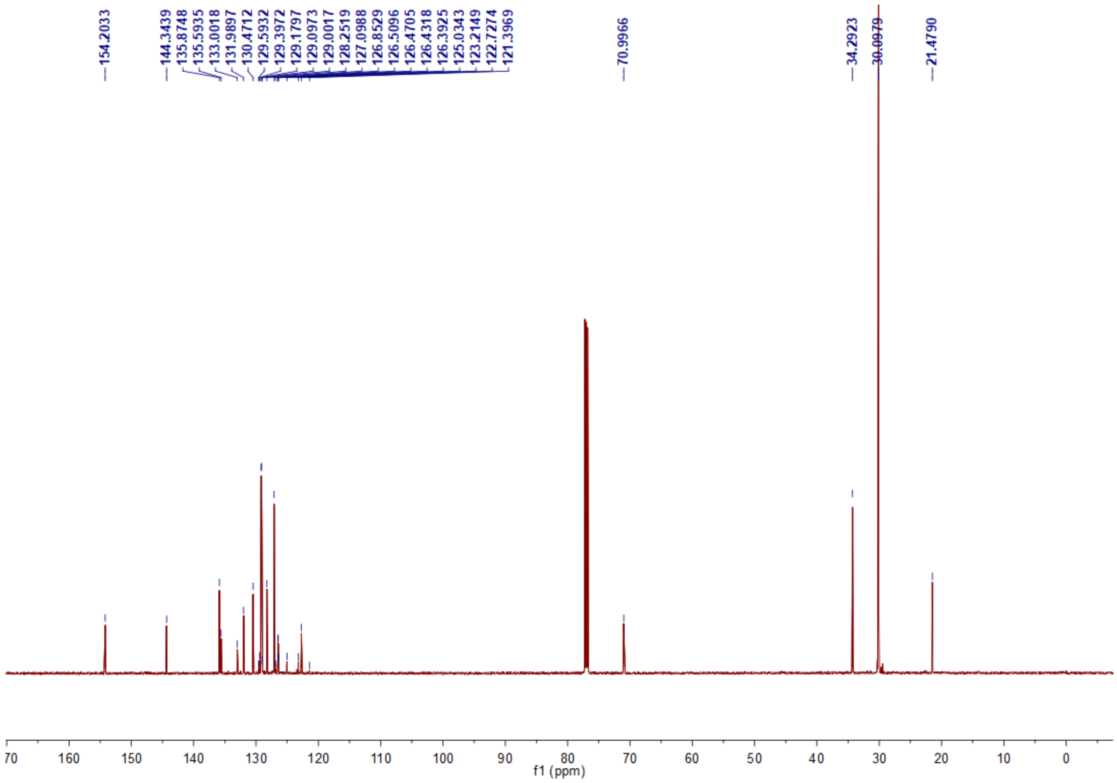
154.2033
144.3439
135.8748
135.5935
133.0018
131.9897
130.4712
129.5932
129.3972
129.1797
129.0973
129.0017
128.2519
127.0988
126.8529
126.5096
126.4705
126.4318
126.3925
125.0343
123.2149
122.7274
121.3969
70.9966
34.2923
30.0979
21.4790
f1 (ppm)

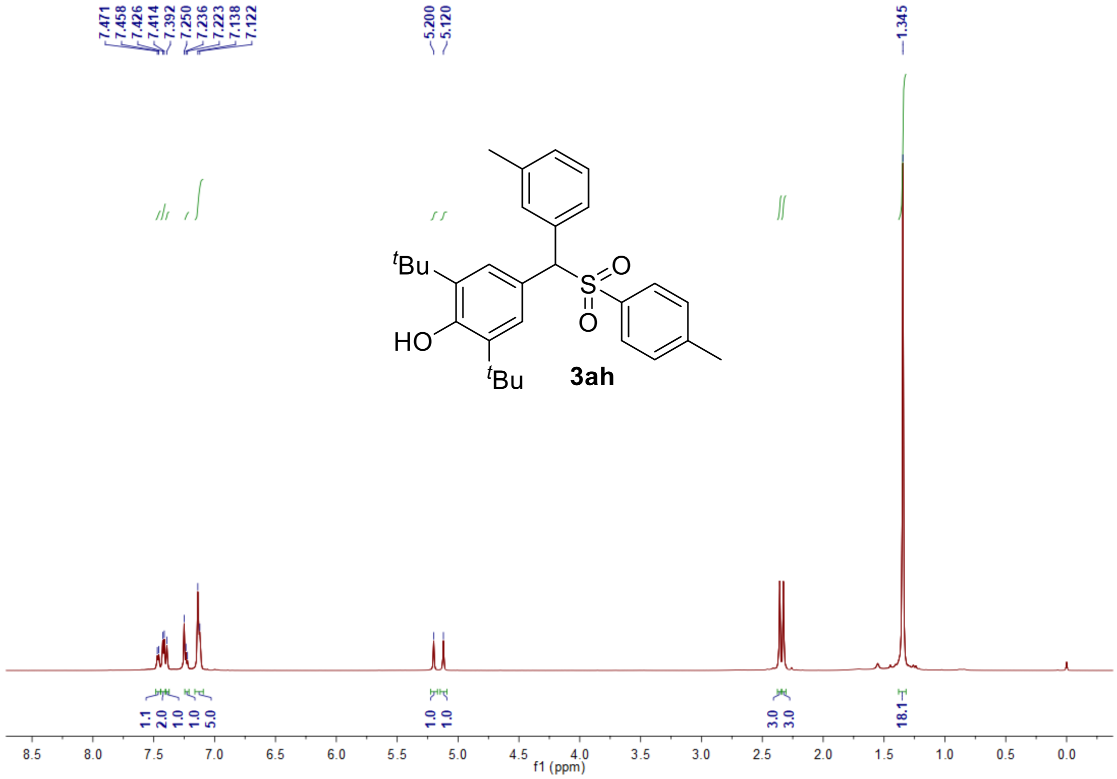

7.471
7.458
7.426
7.414
7.392
7.250
7.236
7.223
7.138
7.122
5.200
5.120
1.345
ᵗBu
HO
ᵗBu
3ah
1.1
2.0
1.0
5.0
1.0
1.0
3.0
3.0
18.1
8.5
8.0
7.5
7.0
6.5
6.0
5.5
5.0
4.5
4.0
3.5
3.0
2.5
2.0
1.5
1.0
0.5
0.0
f1 (ppm)

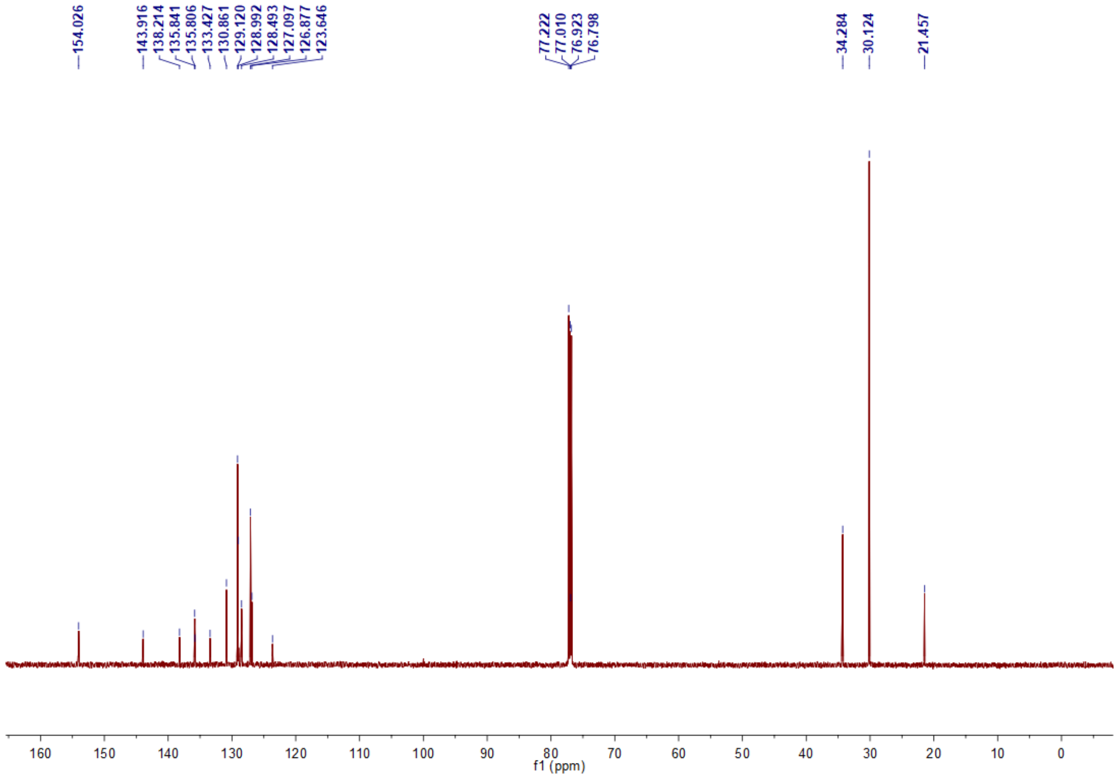

154.026
143.916
138.214
135.841
135.806
133.427
130.861
129.120
128.992
128.493
127.097
126.877
123.646
77.222
77.010
76.923
76.798
34.284
30.124
21.457
160
150
140
130
120
110
100
90
80
70
60
50
40
30
20
10
0
f1 (ppm)

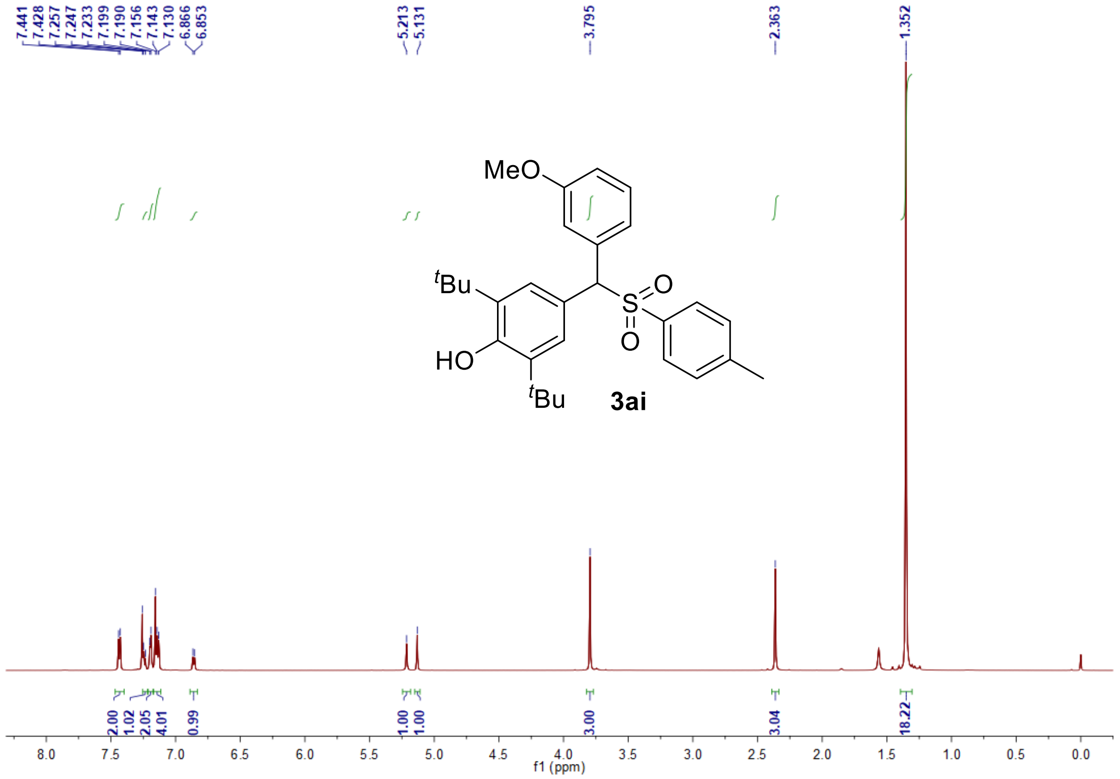
7.441
7.428
7.257
7.247
7.233
7.199
7.190
7.156
7.143
7.130
6.866
6.853
5.213
5.131
3.795
2.363
1.352
MeO
^{t}Bu
HO
^{t}Bu
S
O
O
3ai
2.00
1.02
2.05
4.01
0.99
1.00
1.00
3.00
3.04
18.22
f1 (ppm)

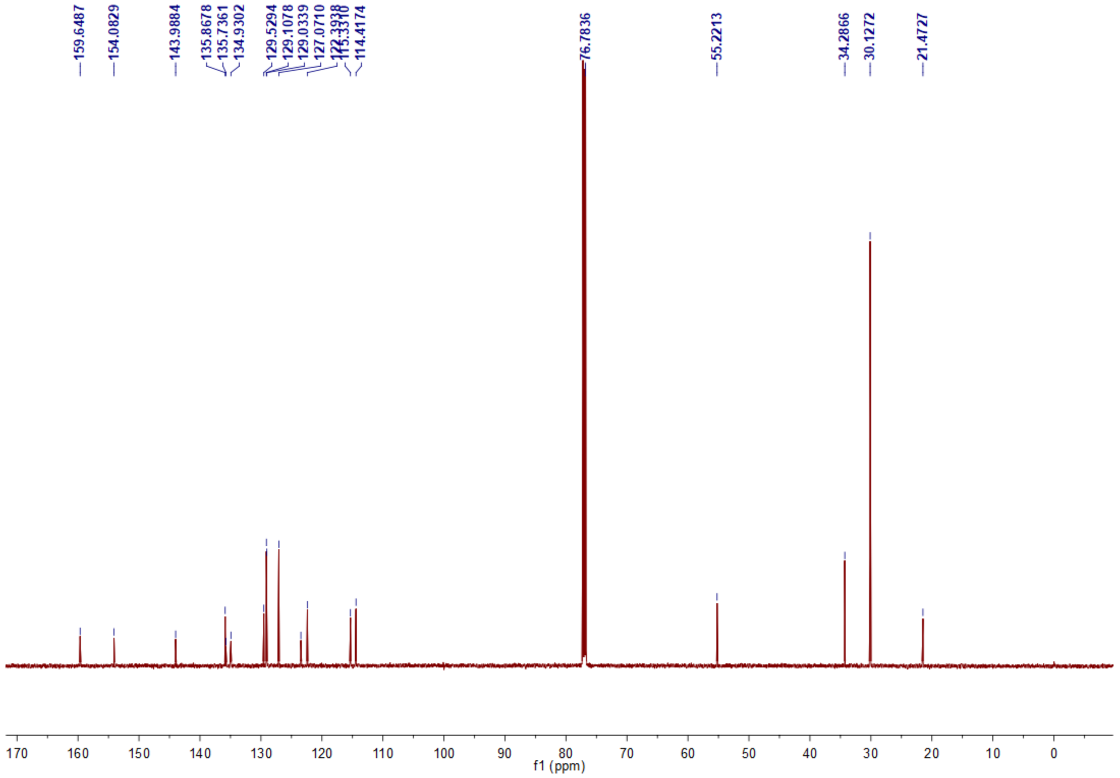
159.6487
154.0829
143.9884
135.8678
135.7361
134.9302
129.5294
129.1078
129.0339
127.0710
122.3938
115.3310
114.4174
76.7836
55.2213
34.2866
30.1272
21.4727
f1 (ppm)

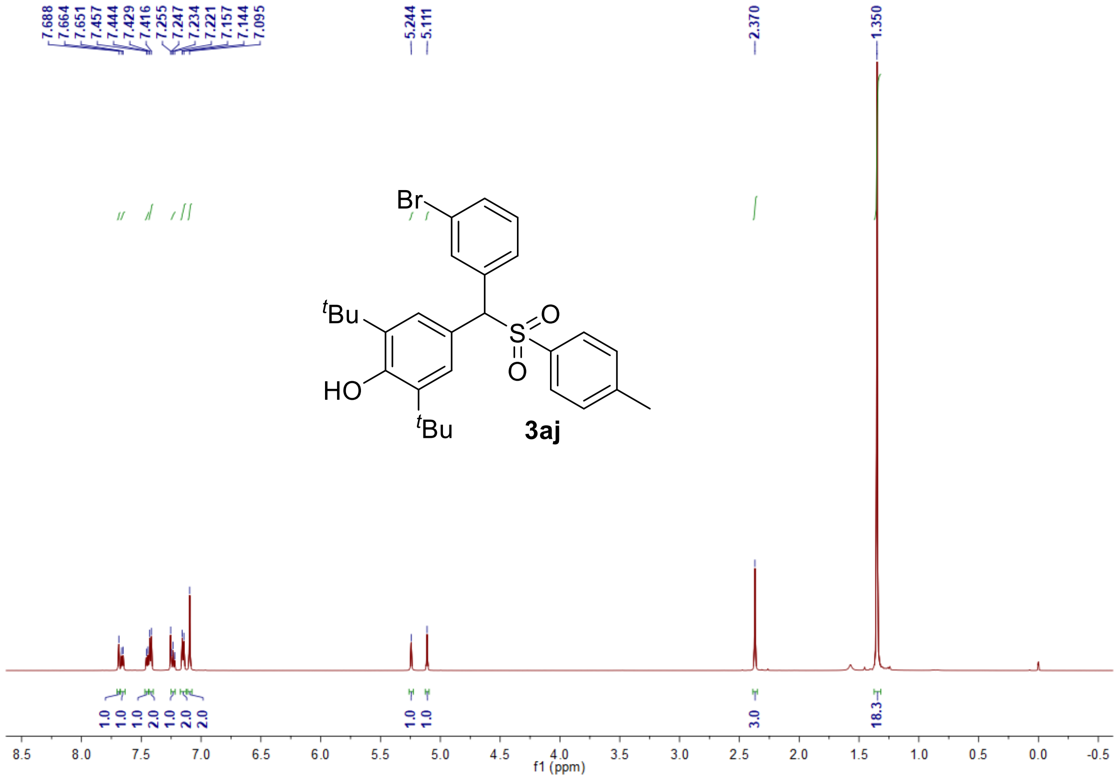
7.688
7.664
7.651
7.457
7.444
7.429
7.416
7.255
7.247
7.234
7.221
7.157
7.144
7.095
5.244
5.111
2.370
1.350
Br
tBu
HO
tBu
3aj
1.0
1.0
1.0
2.0
1.0
2.0
2.0
1.0
1.0
3.0
18.3
8.5 8.0 7.5 7.0 6.5 6.0 5.5 5.0 4.5 4.0 3.5 3.0 2.5 2.0 1.5 1.0 0.5 0.0 -0.5
f1 (ppm)

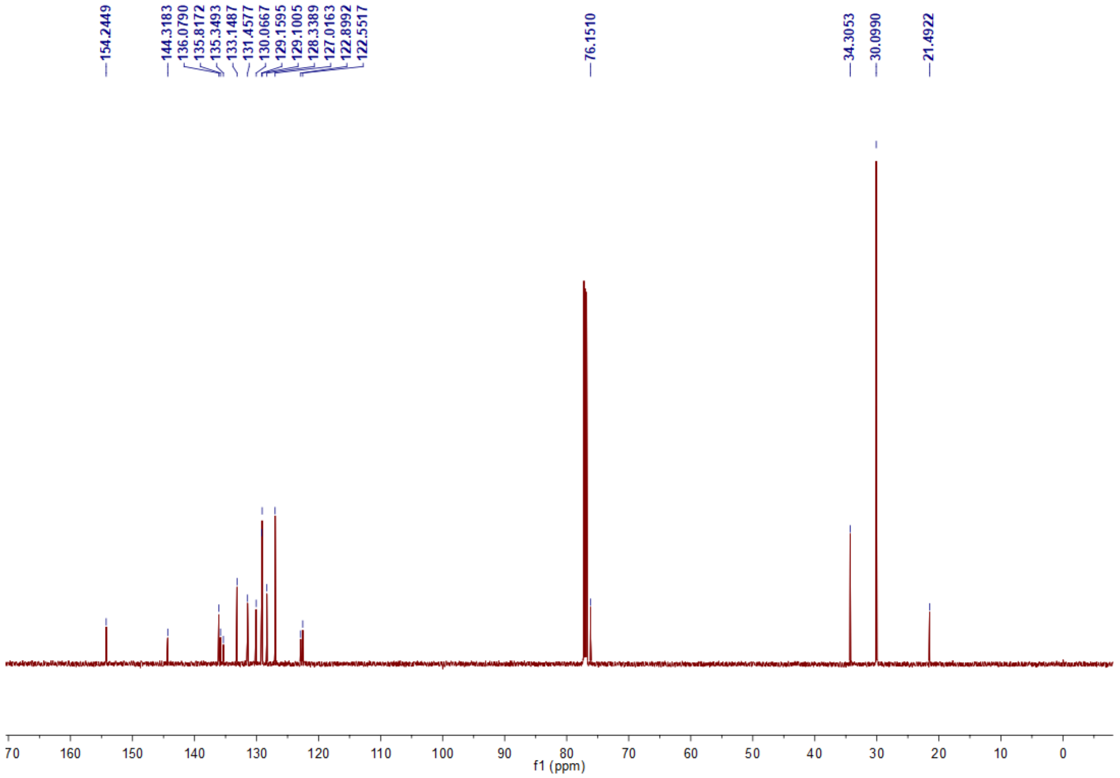
154.2449
144.3183
136.0790
135.8172
135.3493
133.1487
131.4577
130.0667
129.1595
129.1005
128.3389
127.0163
122.8992
122.5517
76.1510
34.3053
30.0990
21.4922
70 160 150 140 130 120 110 100 90 80 70 60 50 40 30 20 10 0
f1 (ppm)

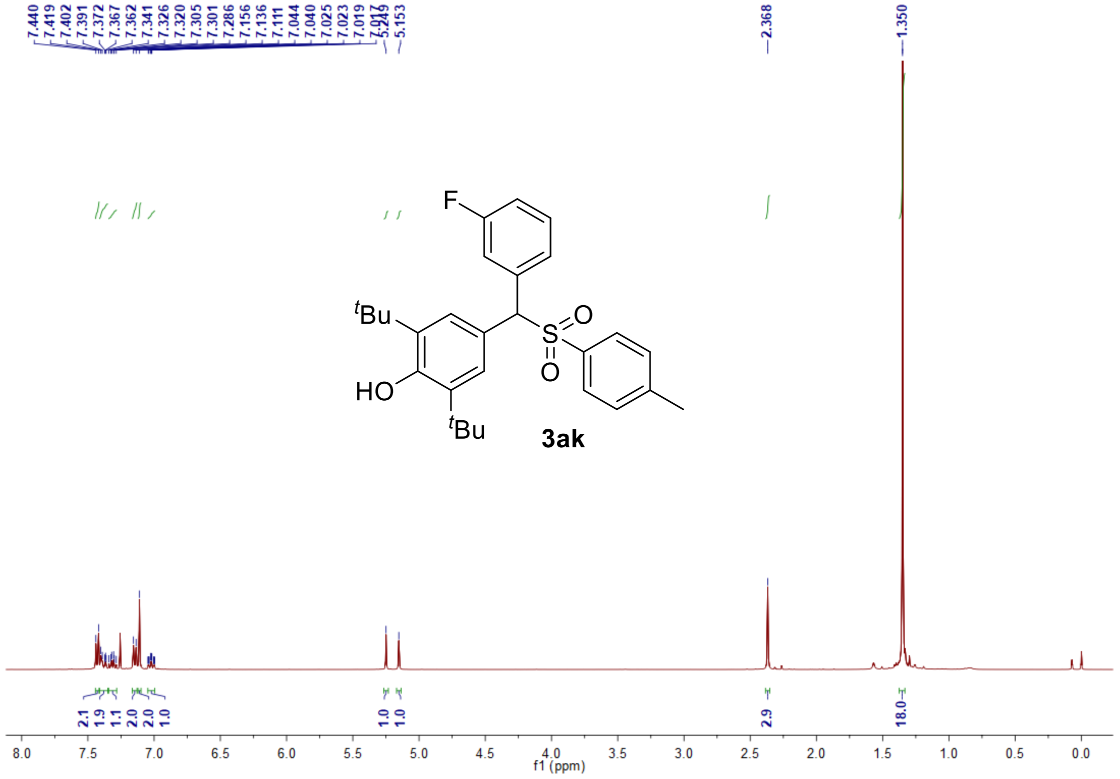
7.440
7.419
7.402
7.391
7.372
7.367
7.362
7.341
7.326
7.320
7.305
7.301
7.286
7.156
7.136
7.111
7.044
7.040
7.025
7.023
7.019
5.153
2.368
1.350
F
tBu
HO
tBu
S
O
O
3ak
2.1
1.9
1.1
2.0
2.0
1.0
1.0
1.0
2.9
18.0
f1 (ppm)

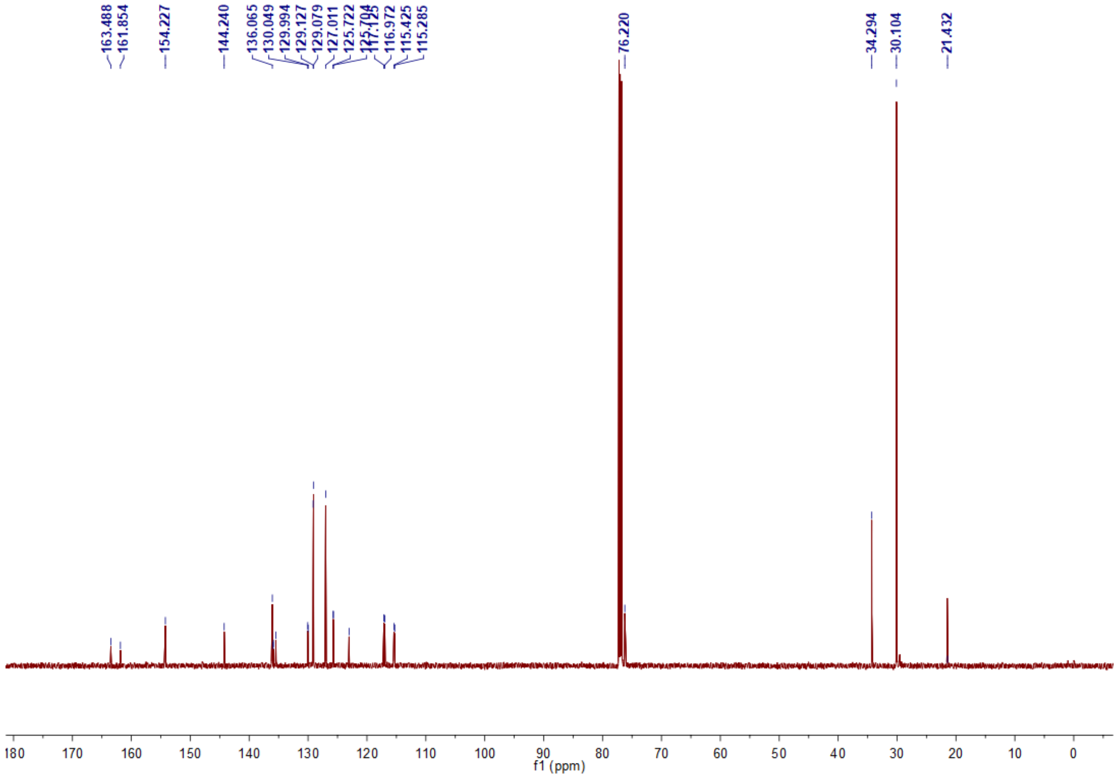
163.488
161.854
154.227
144.240
136.065
130.049
129.994
129.127
129.079
127.011
125.722
116.972
115.425
115.285
76.220
34.294
30.104
21.432
f1 (ppm)

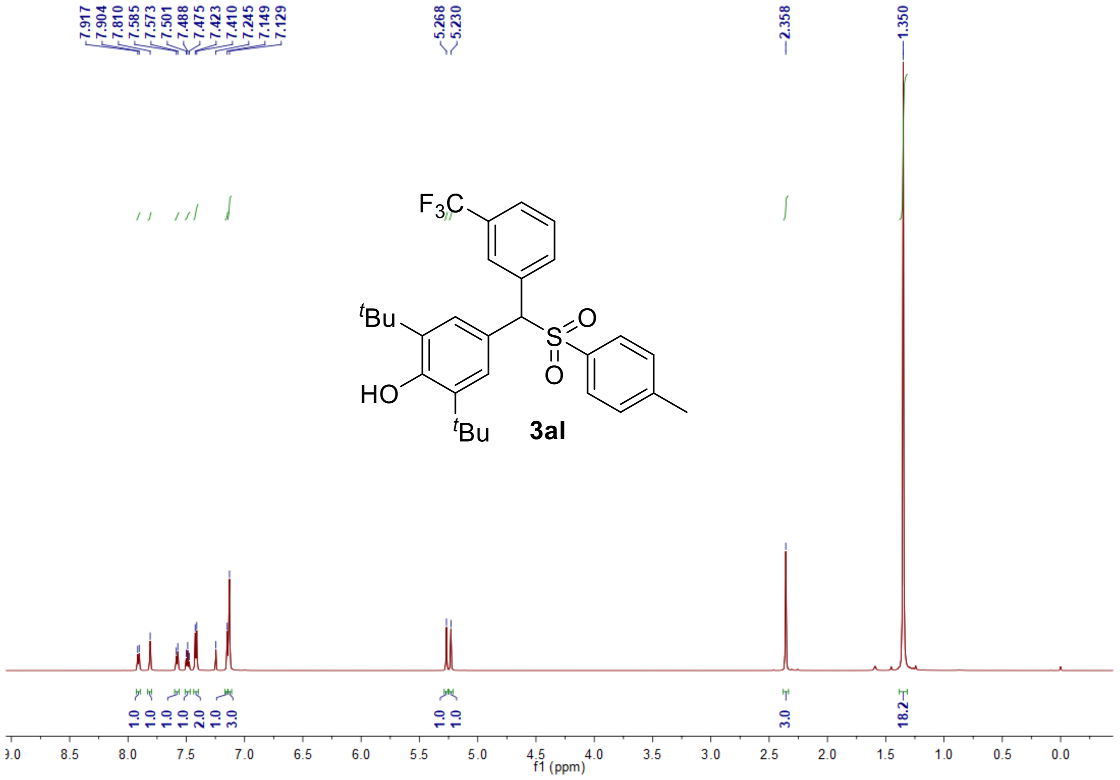
7.917
7.904
7.810
7.585
7.573
7.501
7.488
7.475
7.423
7.410
7.245
7.149
7.129
5.268
5.230
2.358
1.350
F3C
tBu
HO
tBu
S
O
O
3al
1.0
1.0
1.0
1.0
2.0
1.0
3.0
1.0
1.0
3.0
18.2
f1 (ppm)

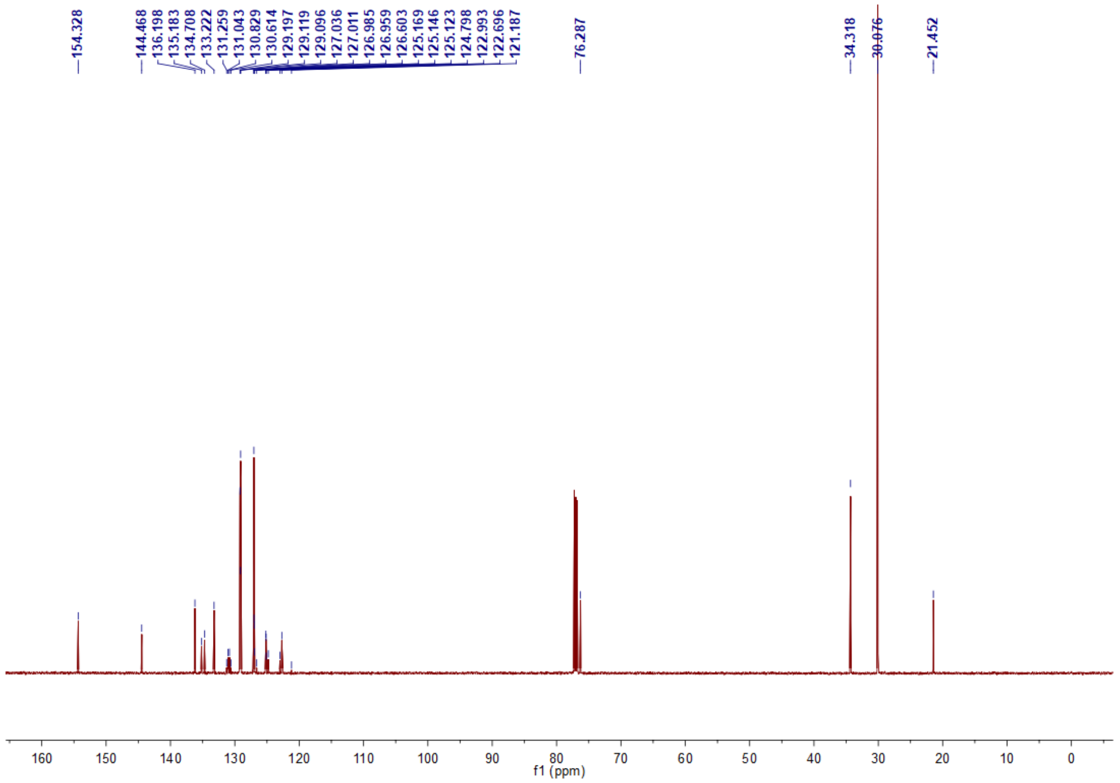
154.328
144.468
136.198
135.183
134.708
133.222
131.259
131.043
130.829
130.614
129.197
129.119
129.096
127.036
127.011
126.985
126.959
126.603
125.169
125.146
125.123
124.798
122.993
122.696
121.187
76.287
34.318
30.076
21.452
f1 (ppm)

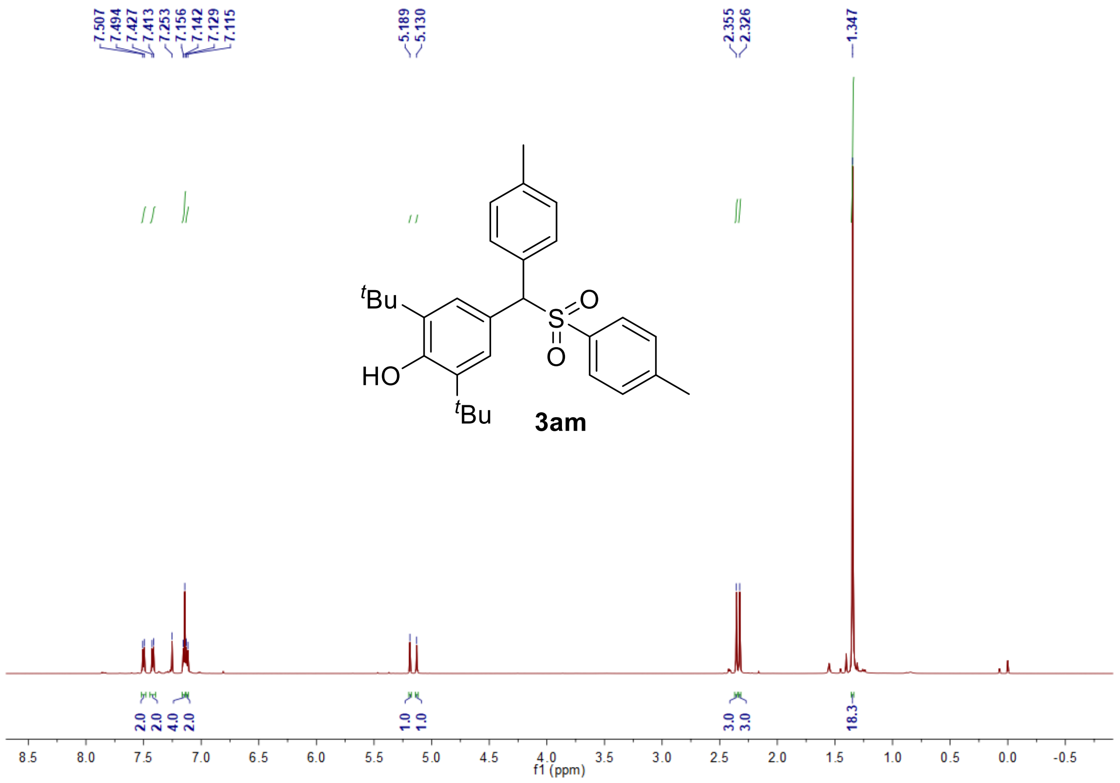
7.507
7.494
7.427
7.413
7.253
7.156
7.142
7.129
7.115
5.189
5.130
2.355
2.326
1.347
tBu
HO
tBu
3am
2.0
2.0
4.0
2.0
1.0
1.0
3.0
3.0
18.3
8.5 8.0 7.5 7.0 6.5 6.0 5.5 5.0 4.5 4.0 3.5 3.0 2.5 2.0 1.5 1.0 0.5 0.0 -0.5
f1 (ppm)

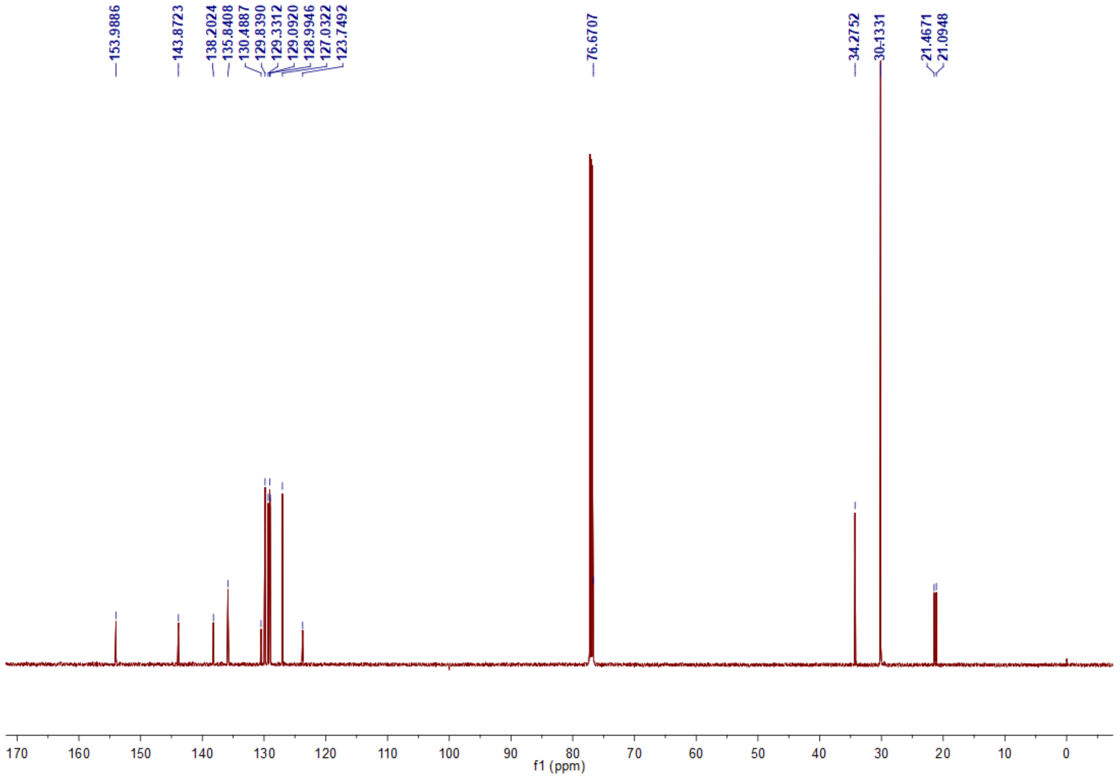
153.9886
143.8723
138.2024
135.8408
130.4887
129.8390
129.3312
129.0920
128.9946
127.0322
123.7492
76.6707
34.2752
30.1331
21.4671
21.0948
170 160 150 140 130 120 110 100 90 80 70 60 50 40 30 20 10 0
f1 (ppm)

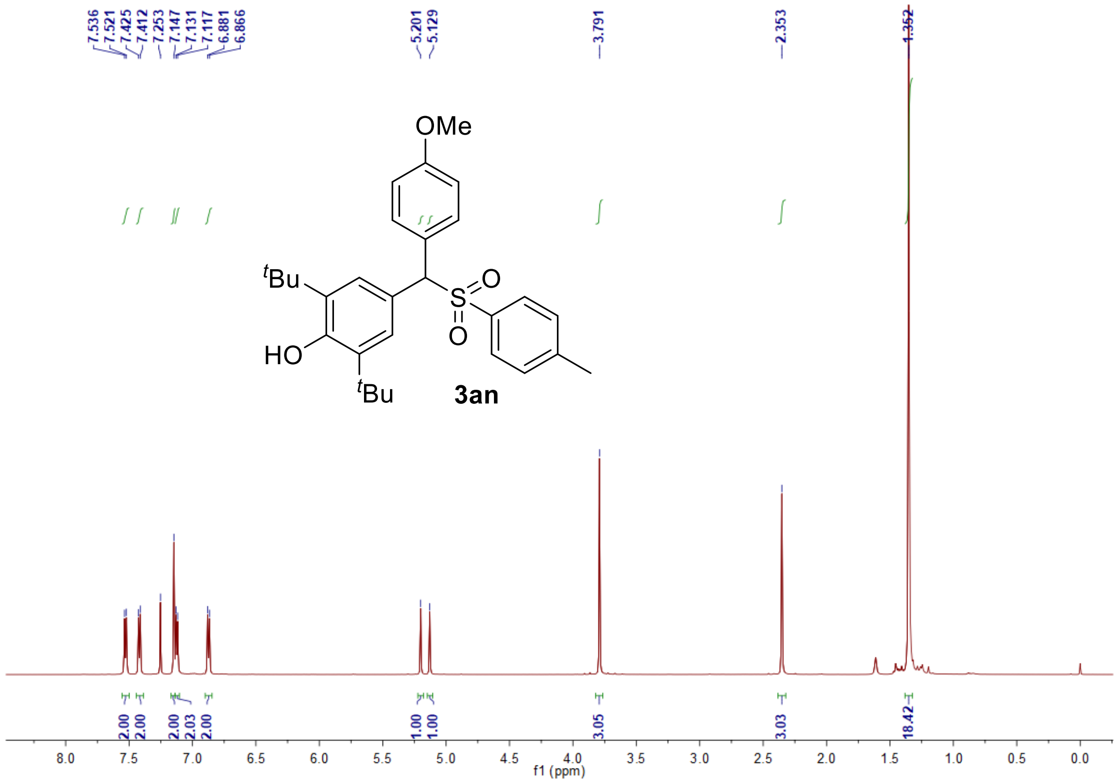
7.536
7.521
7.425
7.412
7.253
7.147
7.131
7.117
6.881
6.866
5.201
5.129
3.791
2.353
1.352
OMe
^{t}Bu
HO
^{t}Bu
S
O
O
3an
2.00
2.00
2.00
2.03
2.00
1.00
1.00
3.05
3.03
18.42
f1 (ppm)

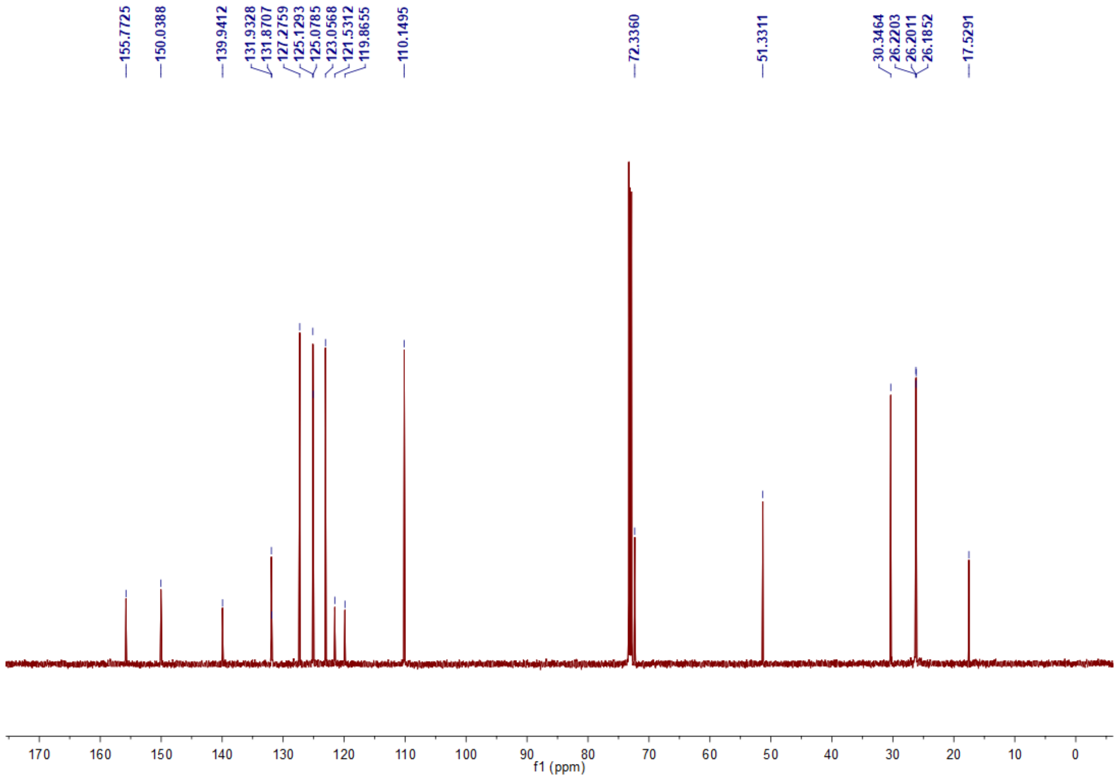
155.7725
150.0388
139.9412
131.9328
131.8707
127.2759
125.1293
125.0785
123.0568
121.5312
119.8655
110.1495
72.3360
51.3311
30.3464
26.2203
26.2011
26.1852
17.5291
f1 (ppm)

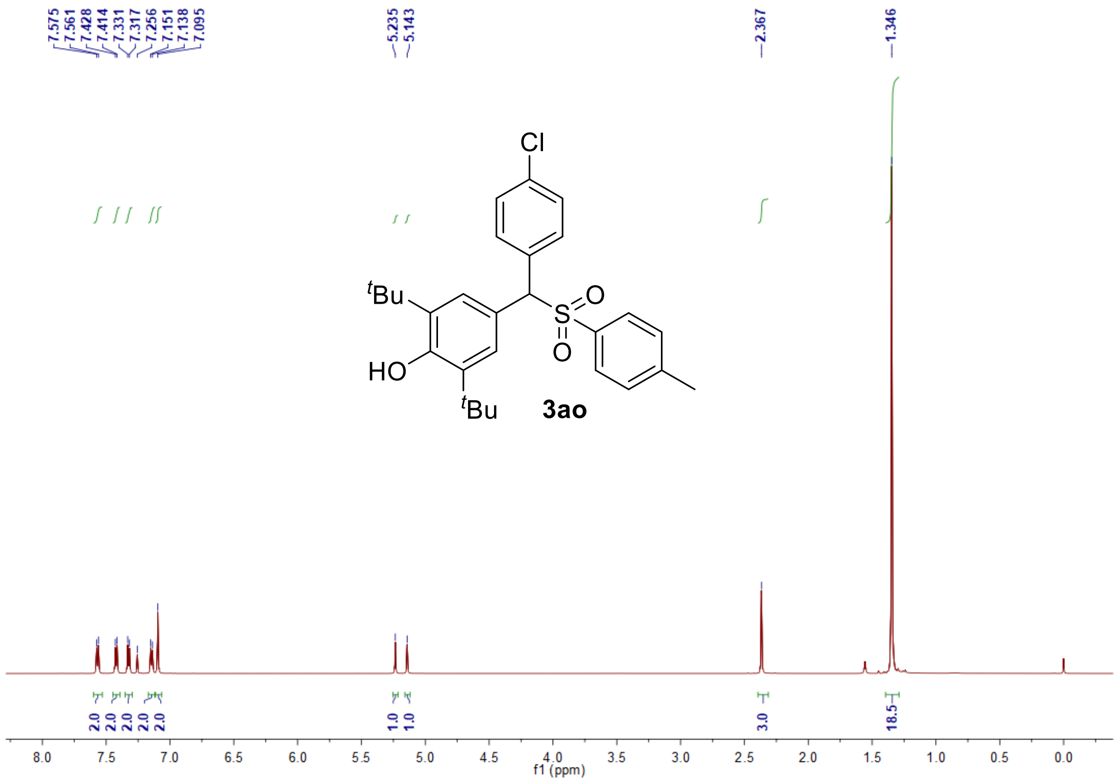
7.575
7.561
7.428
7.414
7.331
7.317
7.256
7.151
7.138
7.095
5.235
5.143
2.367
1.346
Cl
tBu
HO
tBu
S
O
O
3ao
2.0
2.0
2.0
2.0
2.0
1.0
1.0
3.0
18.5
f1 (ppm)

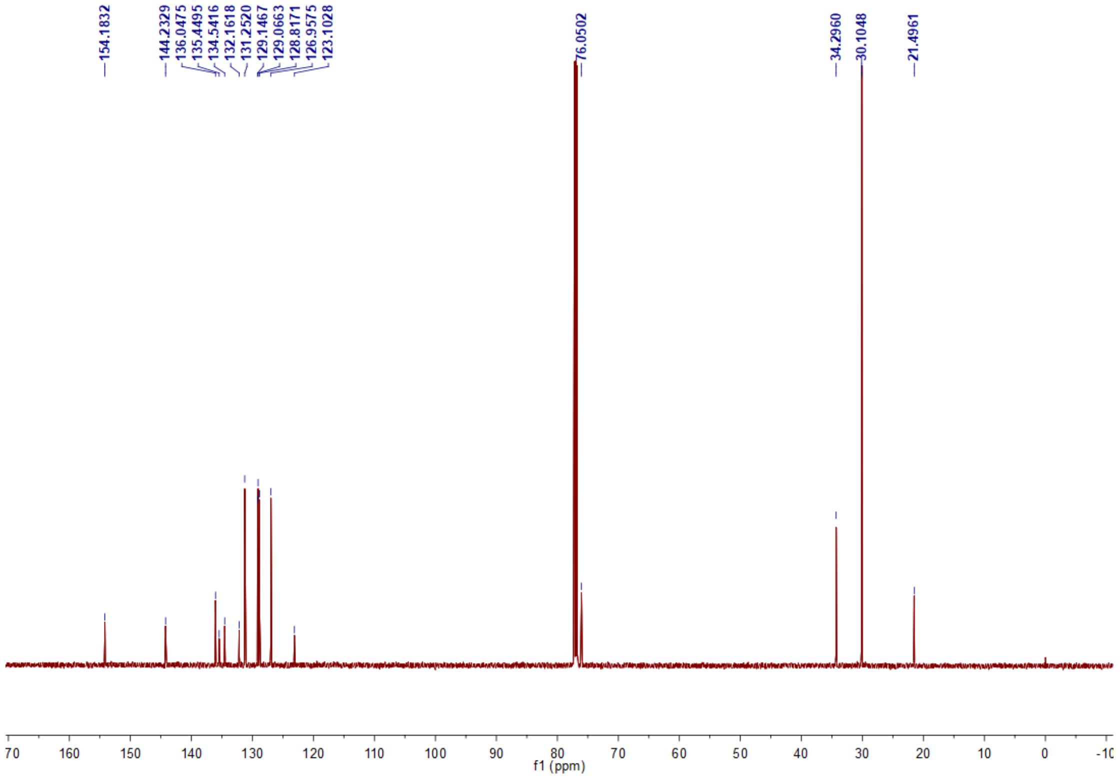
154.1832
144.2329
136.0475
135.4495
134.5416
132.1618
131.2520
129.1467
129.0663
128.8171
126.9575
123.1028
76.0502
34.2960
30.1048
21.4961
f1 (ppm)

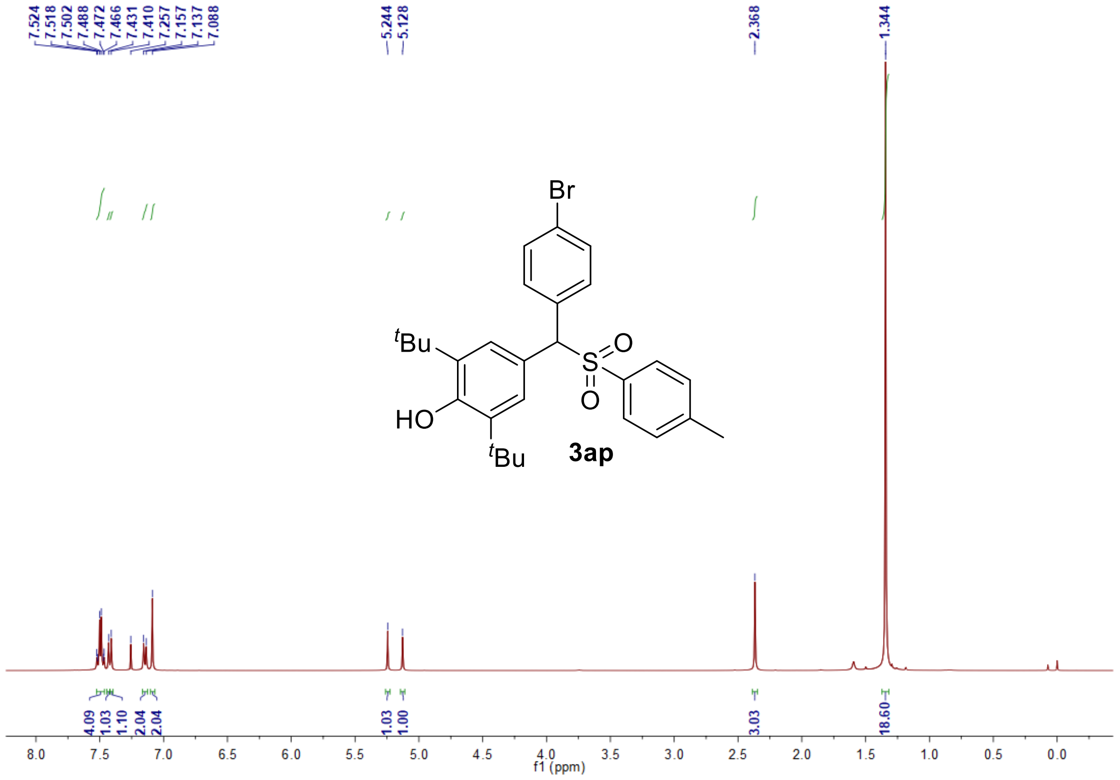
7.524
7.518
7.502
7.488
7.472
7.466
7.431
7.410
7.257
7.157
7.137
7.088
5.244
5.128
2.368
1.344
Br
tBu
HO
tBu
S
O
O
3ap
4.09
1.03
1.10
2.04
2.04
1.03
1.00
3.03
18.60
f1 (ppm)

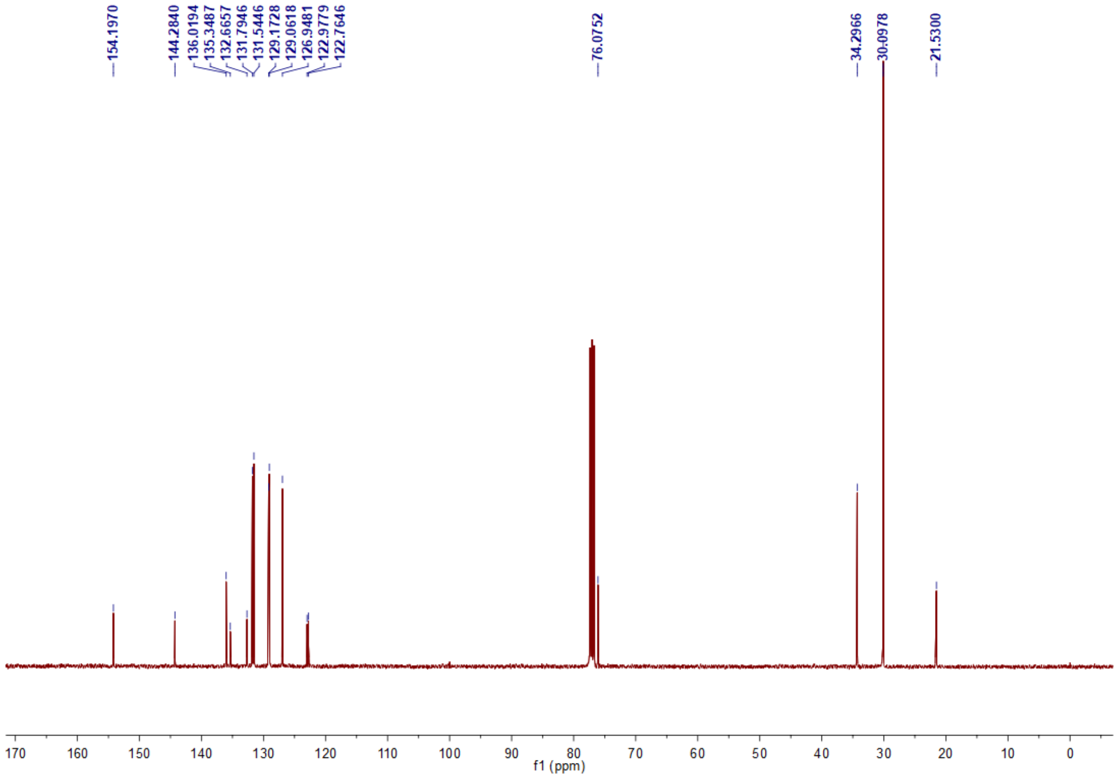
154.1970
144.2840
136.0194
135.3487
132.6657
131.7946
131.5446
129.1728
129.0618
126.9481
122.9779
122.7646
76.0752
34.2966
30.0978
21.5300
f1 (ppm)

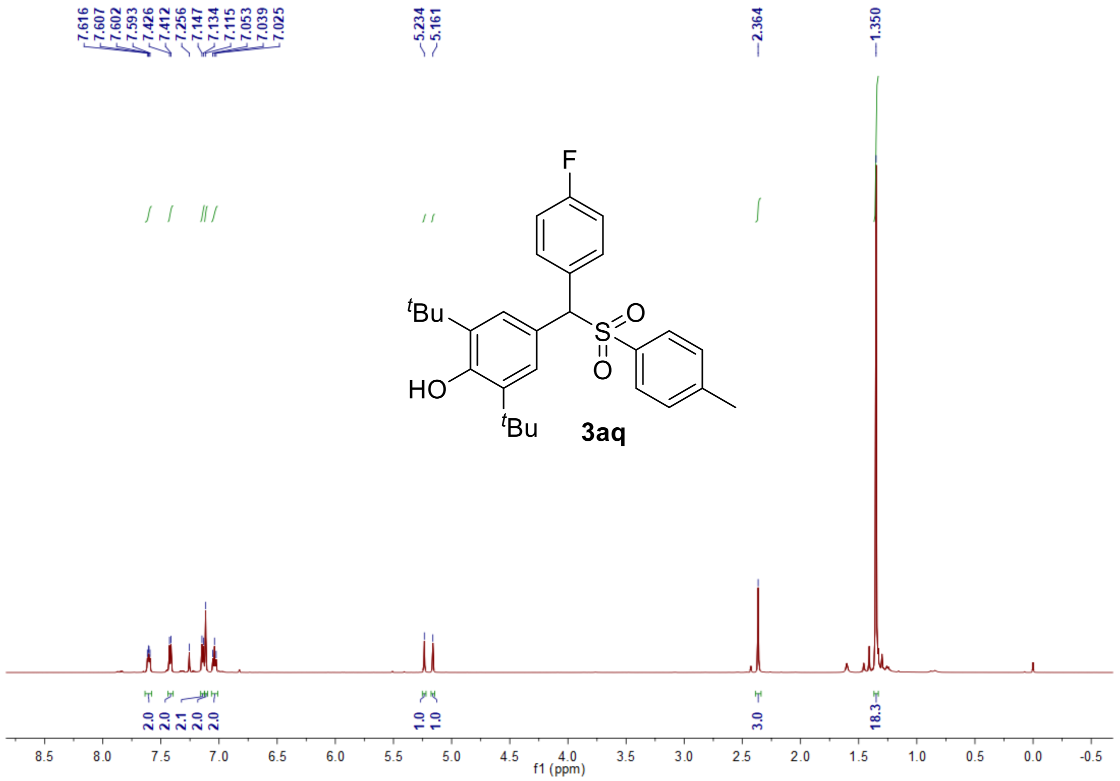
7.616
7.607
7.602
7.593
7.426
7.412
7.256
7.147
7.134
7.115
7.053
7.039
7.025
5.234
5.161
2.364
1.350
F
tBu
HO
tBu
S
O
O
3aq
2.0
2.0
2.1
2.0
2.0
1.0
1.0
3.0
18.3
f1 (ppm)

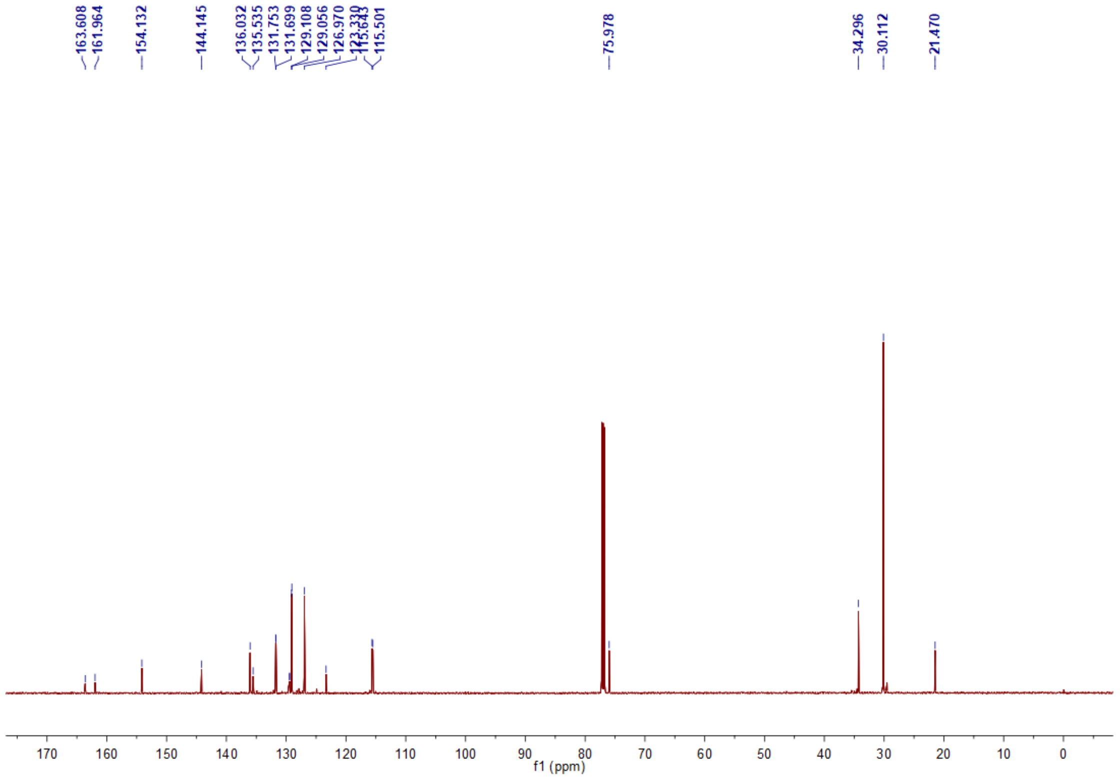
163.608
161.964
154.132
144.145
136.032
135.535
131.753
131.699
129.108
129.056
126.970
115.501
75.978
34.296
30.112
21.470
f1 (ppm)

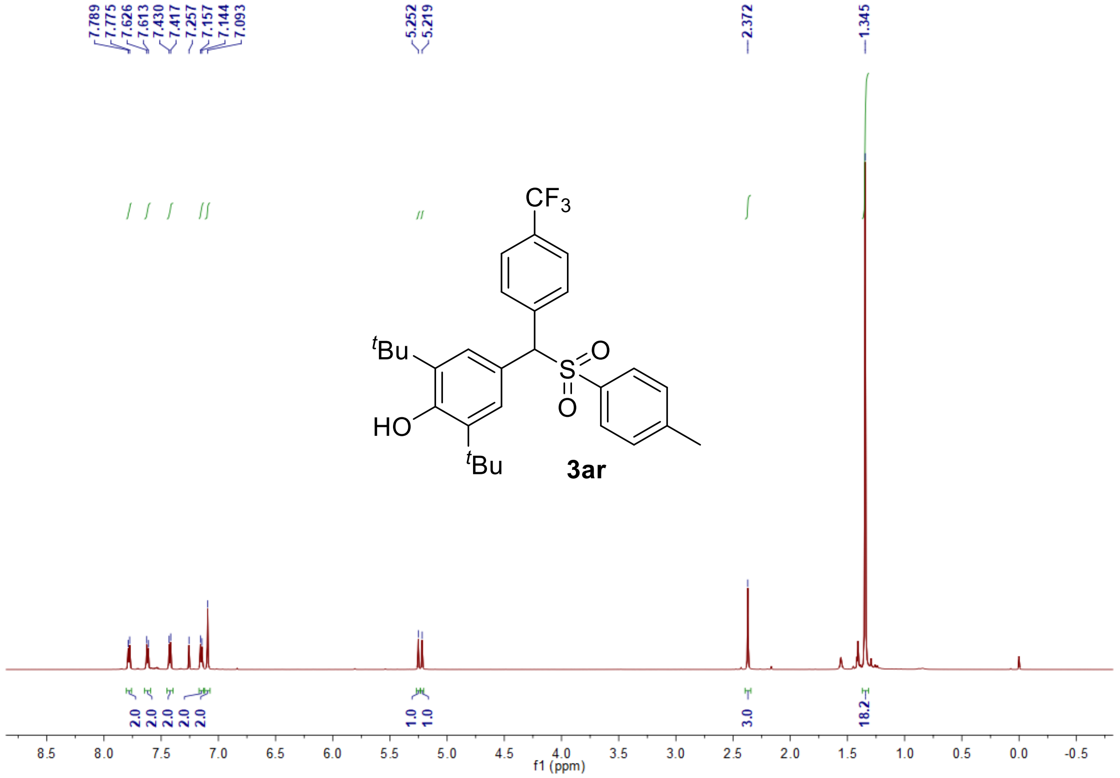
7.789
7.775
7.626
7.613
7.430
7.417
7.257
7.157
7.144
7.093
5.252
5.219
2.372
1.345
CF3
tBu
HO
tBu
S
O
O
3ar
2.0
2.0
2.0
2.0
2.0
1.0
1.0
3.0
18.2
8.5
8.0
7.5
7.0
6.5
6.0
5.5
5.0
4.5
4.0
3.5
3.0
2.5
2.0
1.5
1.0
0.5
0.0
-0.5
f1 (ppm)

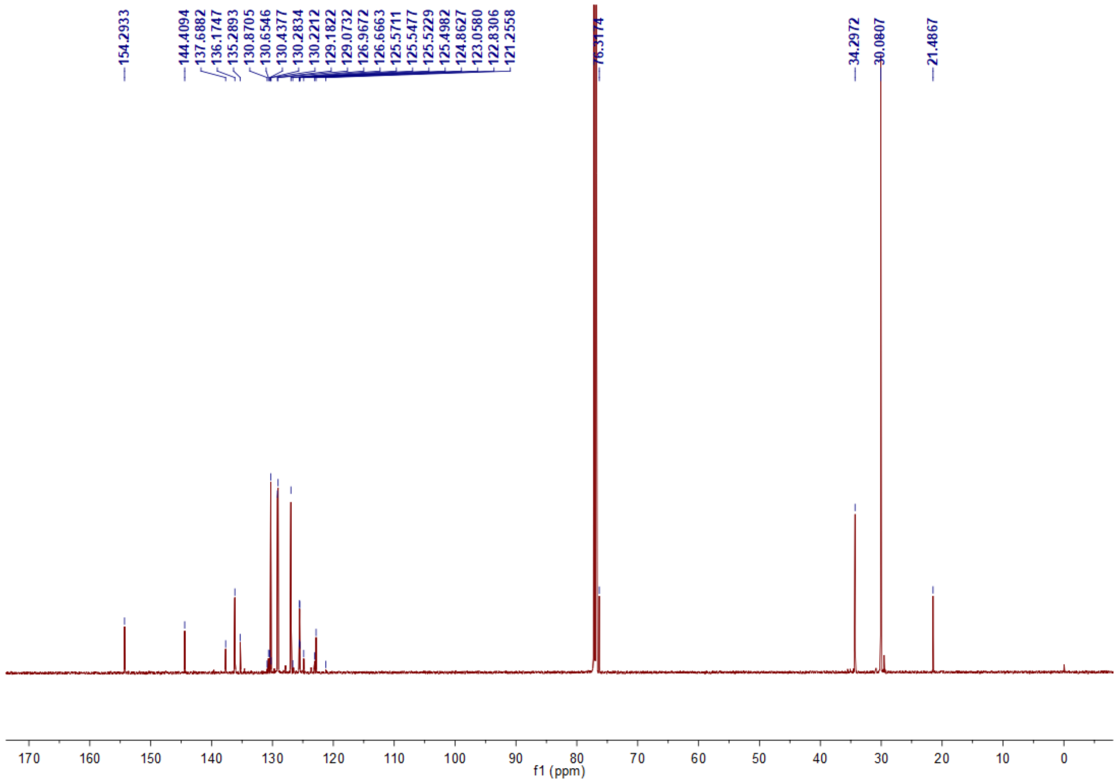
154.2933
144.4094
137.6882
136.1747
135.2893
130.8705
130.6546
130.4377
130.2834
130.2212
129.1822
129.0732
126.9672
126.6663
125.5711
125.5477
125.5229
125.4982
124.8627
123.0580
122.8306
121.2558
76.5174
34.2972
30.0807
21.4867
170
160
150
140
130
120
110
100
90
80
70
60
50
40
30
20
10
0
f1 (ppm)

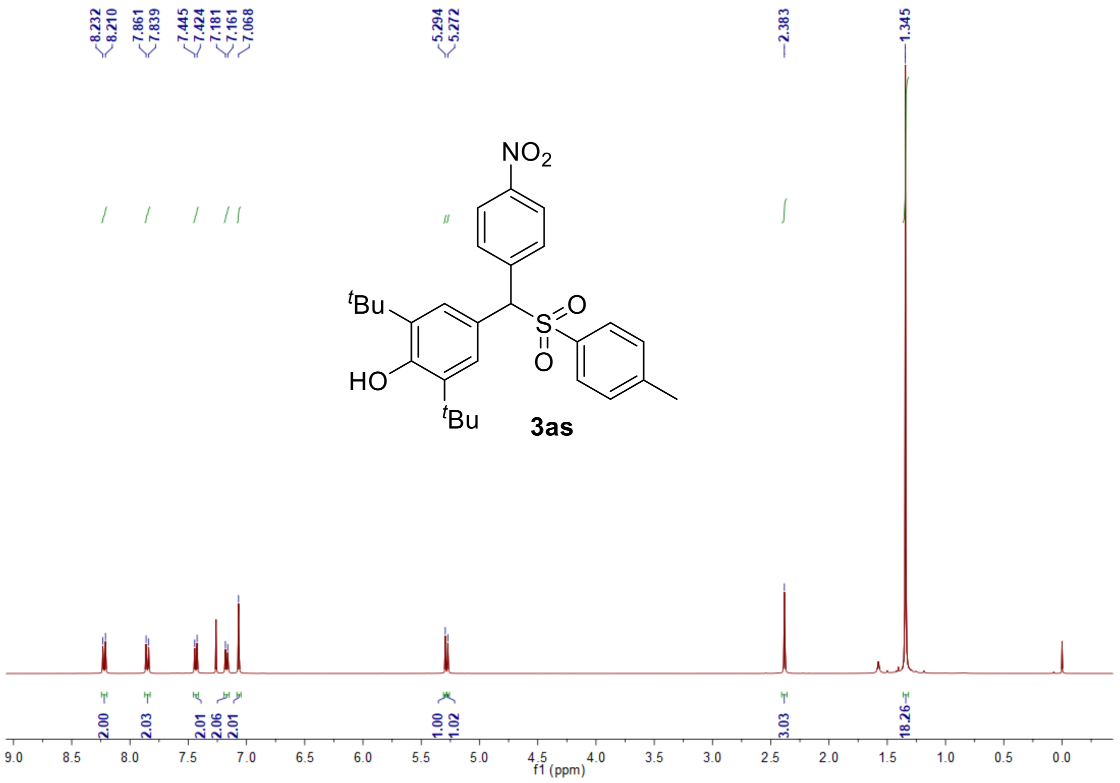
8.232
8.210
7.861
7.839
7.445
7.424
7.181
7.161
7.068
5.294
5.272
2.383
1.345
NO2
tBu
HO
tBu
S
O
O
3as
2.00
2.03
2.01
2.06
2.01
1.00
1.02
3.03
18.26
9.0 8.5 8.0 7.5 7.0 6.5 6.0 5.5 5.0 4.5 4.0 3.5 3.0 2.5 2.0 1.5 1.0 0.5 0.0
f1 (ppm)

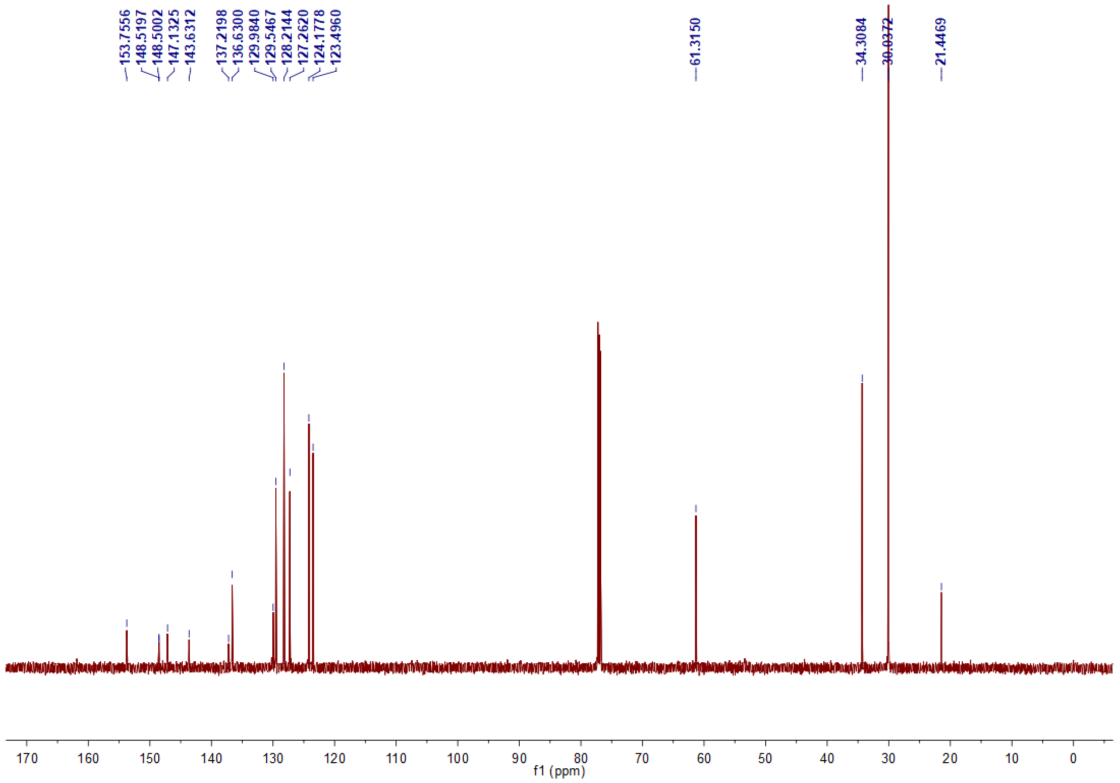
153.7556
148.5197
148.5002
147.1325
143.6312
137.2198
136.6300
129.9840
129.5467
128.2144
127.2620
124.1778
123.4960
61.3150
34.3084
30.0372
21.4469
170 160 150 140 130 120 110 100 90 80 70 60 50 40 30 20 10 0
f1 (ppm)

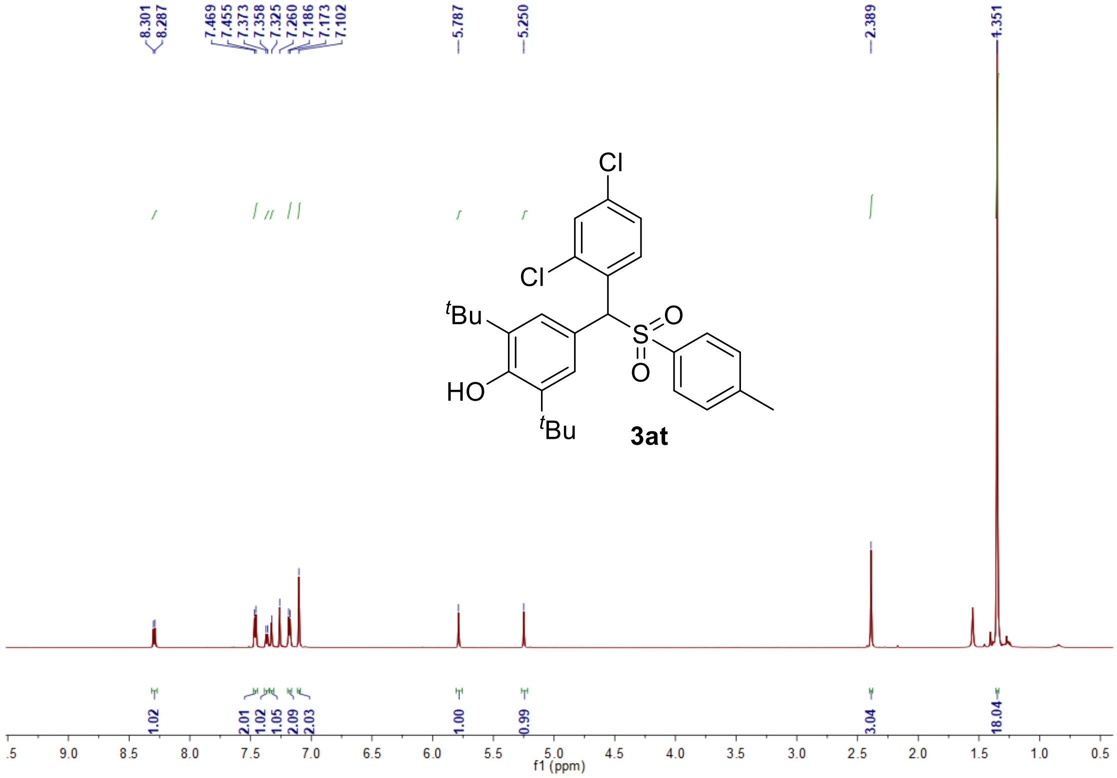

8.301
8.287
7.469
7.455
7.373
7.358
7.325
7.260
7.186
7.173
7.102
5.787
5.250
2.389
1.351
Cl
Cl
tBu
HO
tBu
S
O
O
3at
1.02
2.01
1.02
1.05
2.09
2.03
1.00
0.99
3.04
18.04
f1 (ppm)

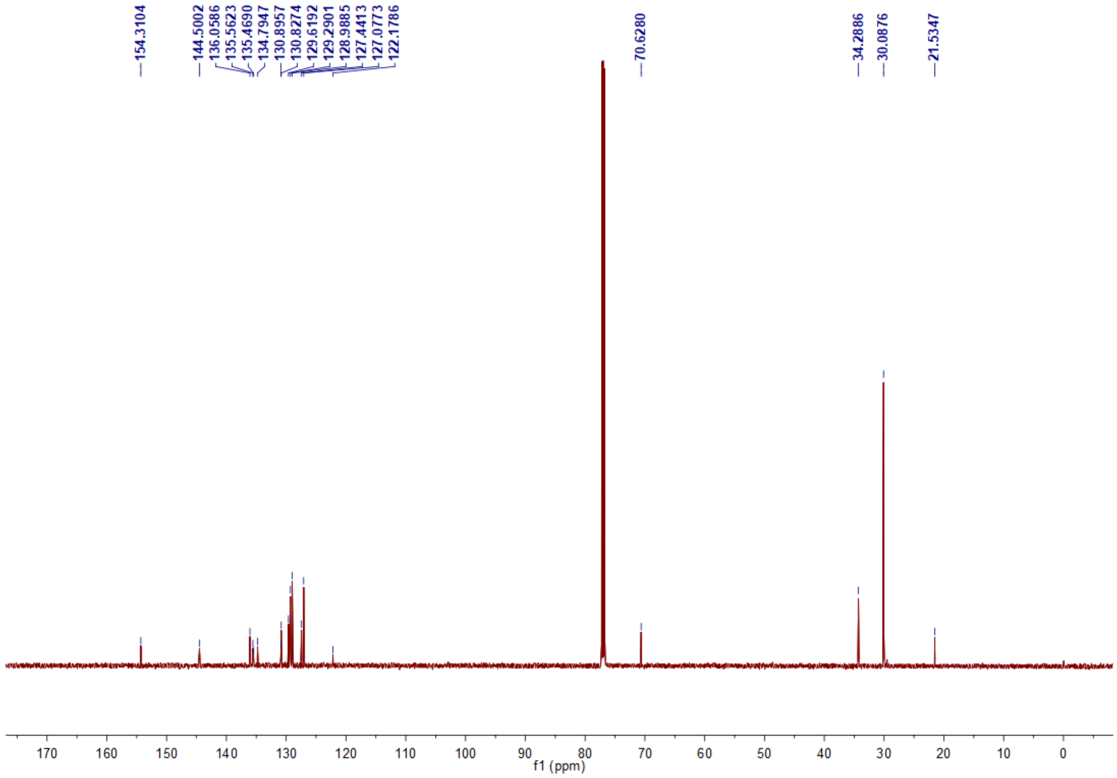

154.3104
144.5002
136.0586
135.5623
135.4690
134.7947
130.8957
130.8274
129.6192
129.2901
128.9885
127.4413
127.0773
122.1786
70.6280
34.2886
30.0876
21.5347
f1 (ppm)

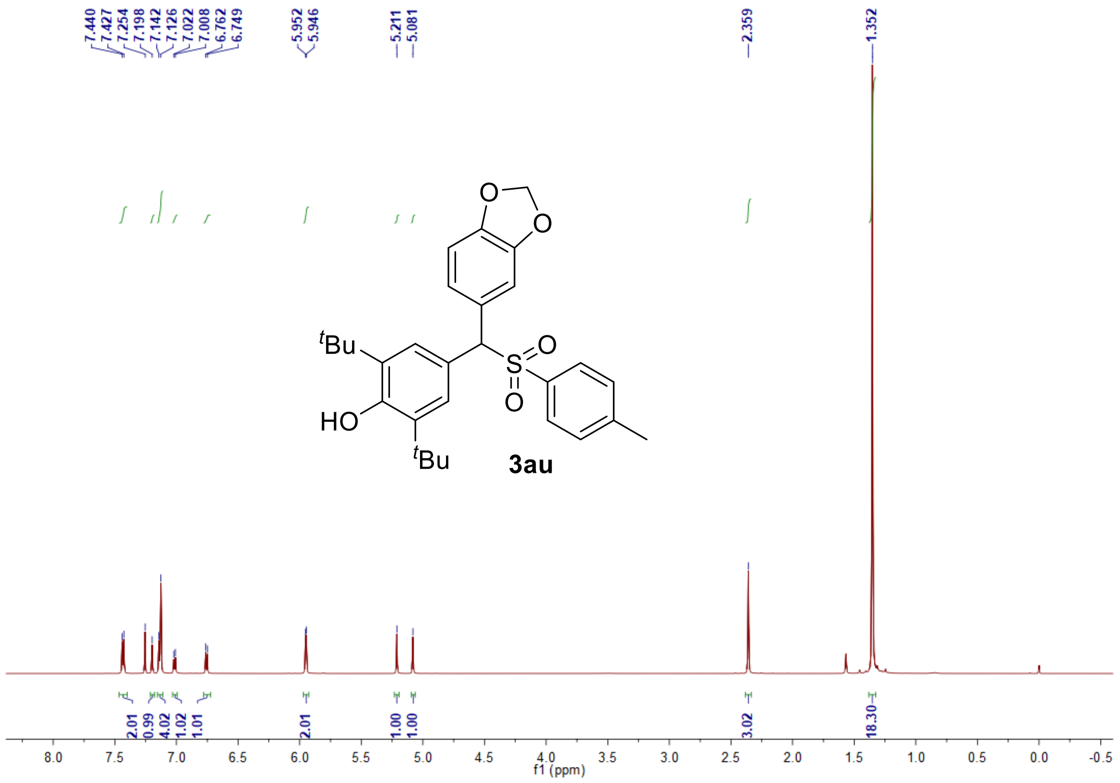
7.440
7.427
7.254
7.198
7.142
7.126
7.022
7.008
6.762
6.749
5.952
5.946
5.211
5.081
2.359
1.352
tBu
HO
tBu
3au
2.01
0.99
4.02
1.02
1.01
2.01
1.00
1.00
3.02
18.30
8.0 7.5 7.0 6.5 6.0 5.5 5.0 4.5 4.0 3.5 3.0 2.5 2.0 1.5 1.0 0.5 0.0 -0.5
f1 (ppm)

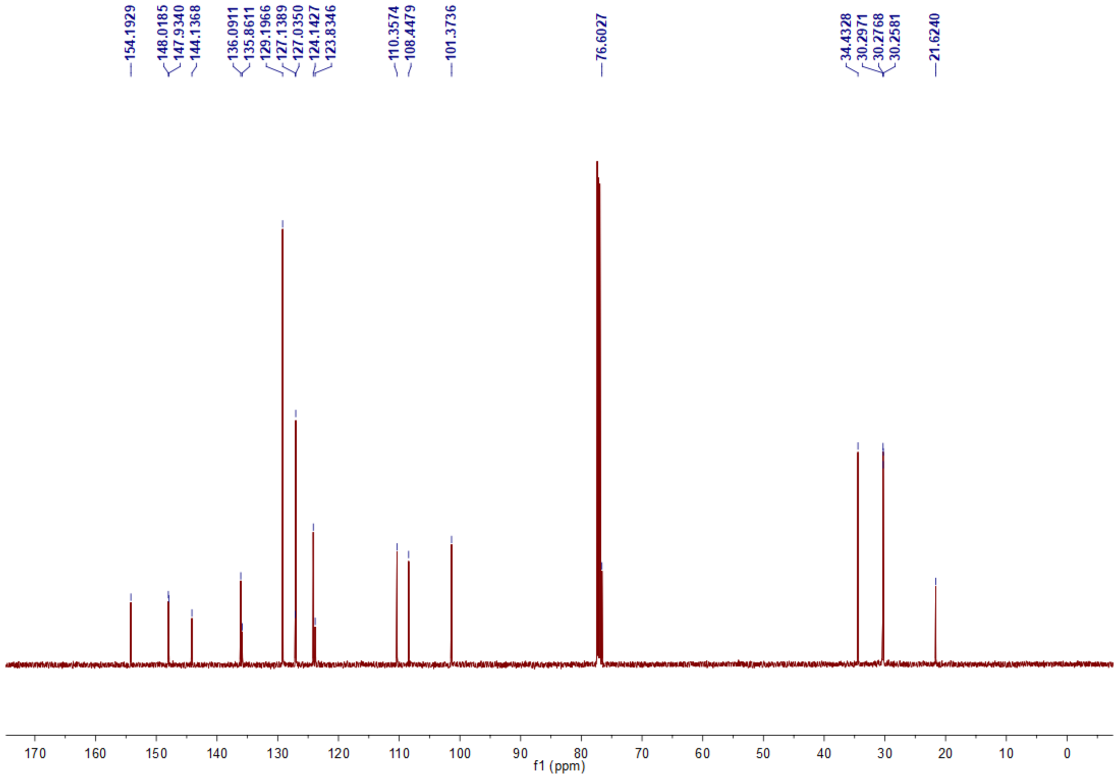
154.1929
148.0185
147.9340
144.1368
136.0911
135.8611
129.1966
127.1389
127.0350
124.1427
123.8346
110.3574
108.4479
101.3736
76.6027
34.4328
30.2971
30.2768
30.2581
21.6240
170 160 150 140 130 120 110 100 90 80 70 60 50 40 30 20 10 0
f1 (ppm)

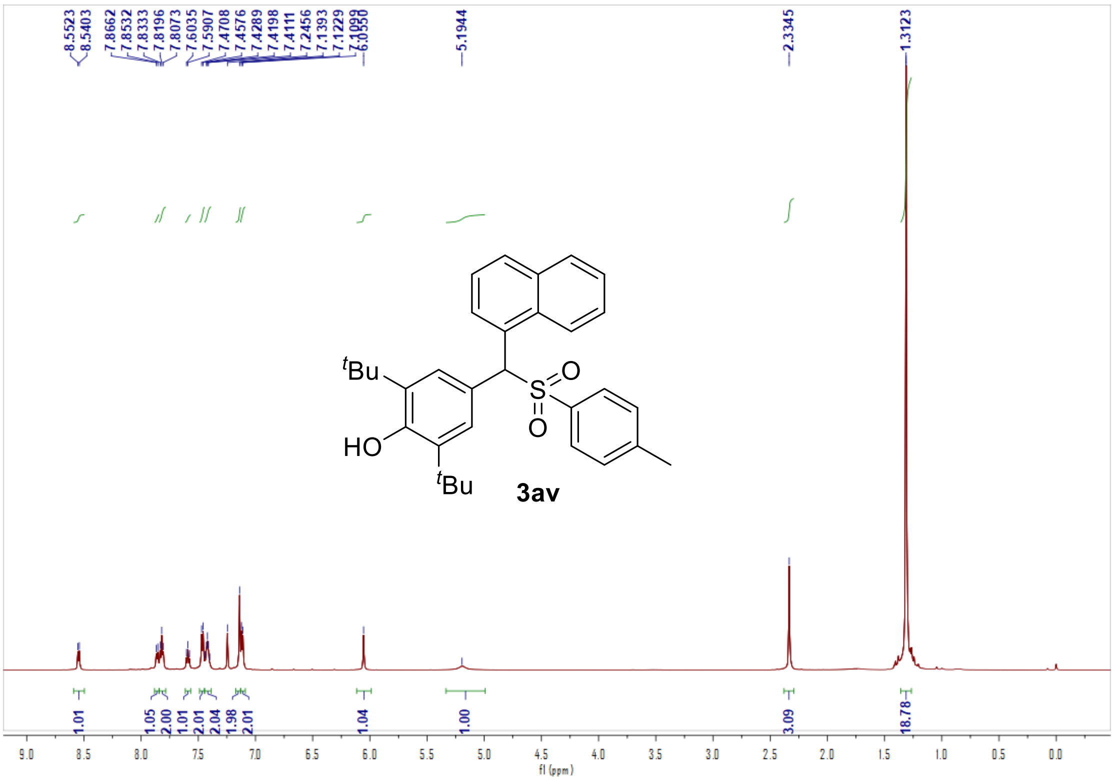
8.5523
8.5403
7.8662
7.8532
7.8333
7.8196
7.8073
7.6035
7.5907
7.4708
7.4576
7.4289
7.4198
7.4111
7.2456
7.1393
7.1229
5.1944
2.3345
1.3123
tBu
HO
tBu
3av
1.01
1.05
2.00
1.01
2.01
2.04
1.98
2.01
1.04
1.00
3.09
18.78
f1 (ppm)

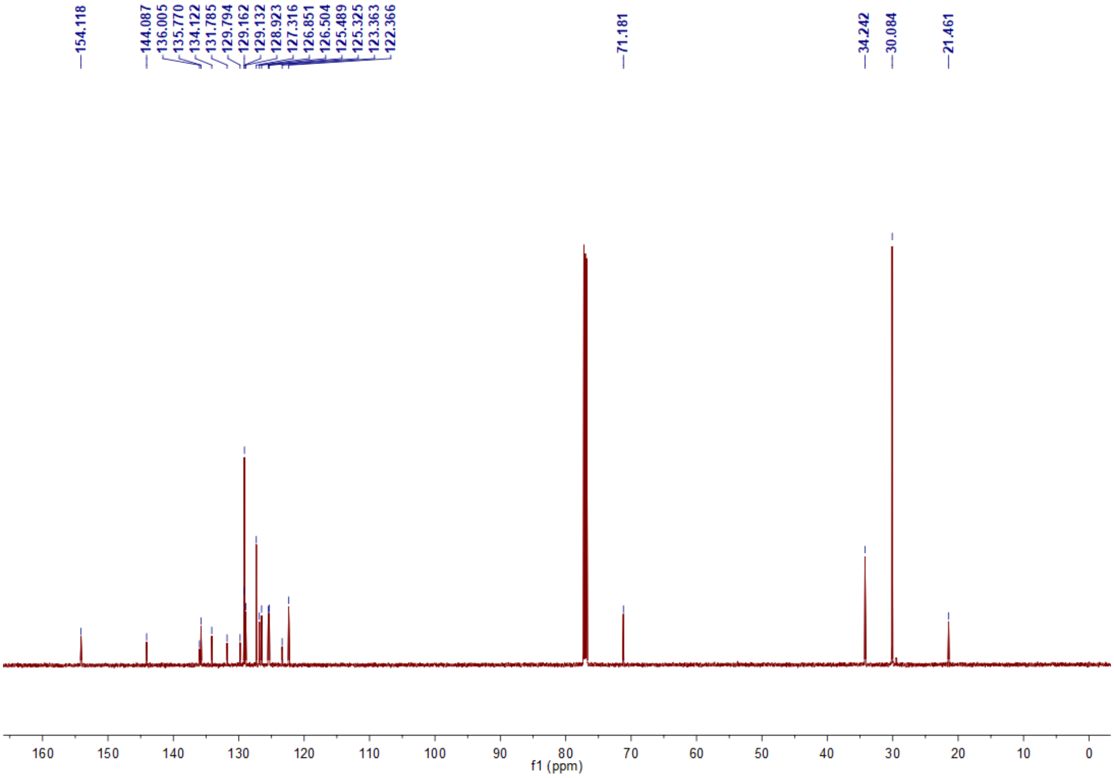
154.118
144.087
136.005
135.770
134.122
131.785
129.794
129.162
129.132
128.923
127.316
126.851
126.504
125.489
125.325
123.363
122.366
71.181
34.242
30.084
21.461
f1 (ppm)

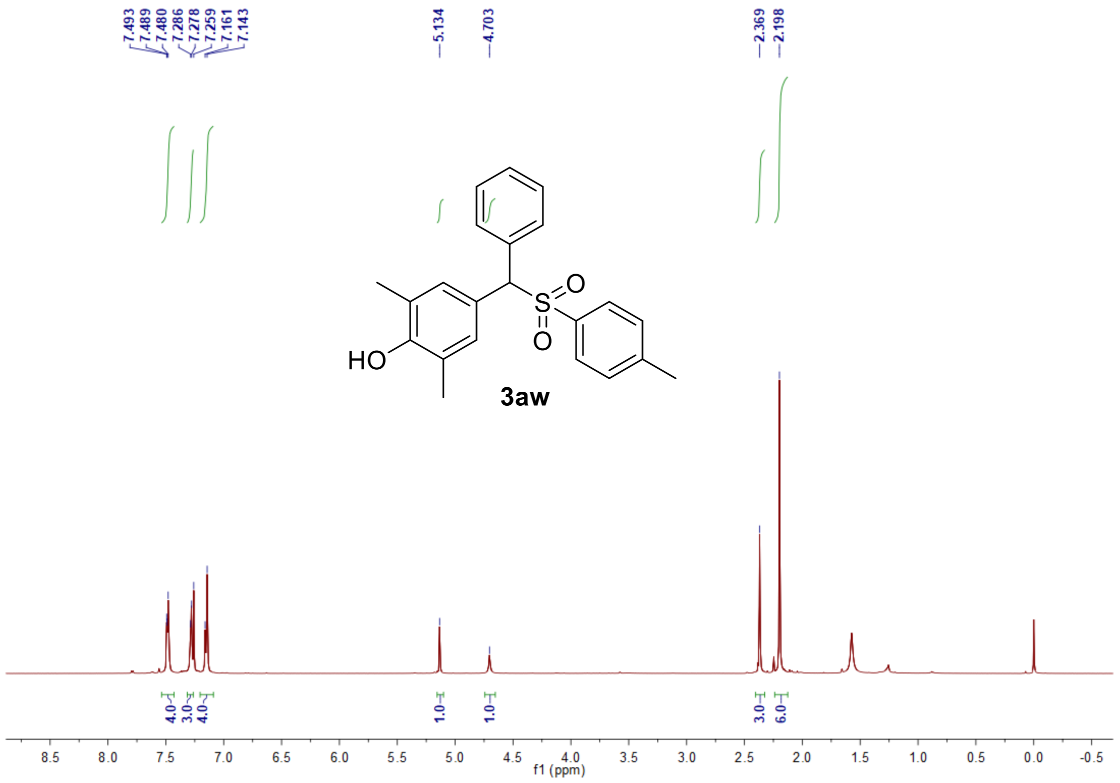
7.493
7.489
7.480
7.286
7.278
7.259
7.161
7.143
5.134
4.703
2.369
2.198
HO
O
S
O
3aw
4.0
3.0
4.0
1.0
1.0
3.0
6.0
8.5 8.0 7.5 7.0 6.5 6.0 5.5 5.0 4.5 4.0 3.5 3.0 2.5 2.0 1.5 1.0 0.5 0.0 -0.5
f1 (ppm)

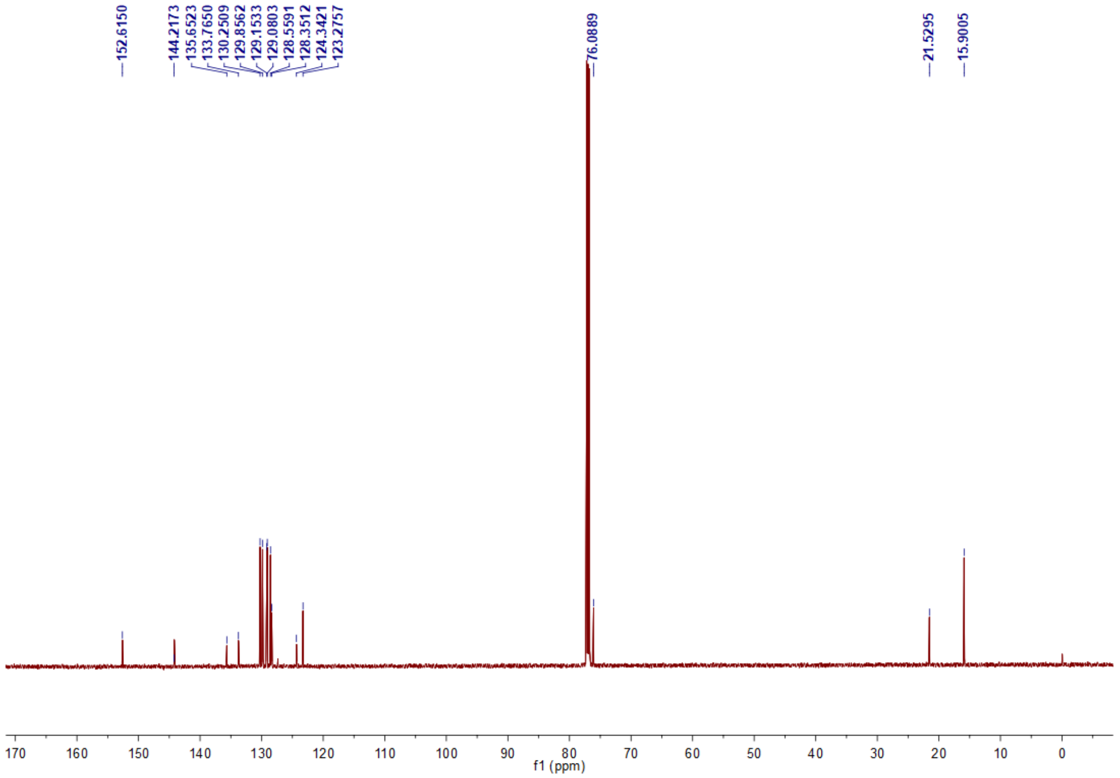
152.6150
144.2173
135.6523
133.7650
130.2509
129.8562
129.1533
129.0803
128.5591
128.3512
124.3421
123.2757
76.0889
21.5295
15.9005
170 160 150 140 130 120 110 100 90 80 70 60 50 40 30 20 10 0
f1 (ppm)

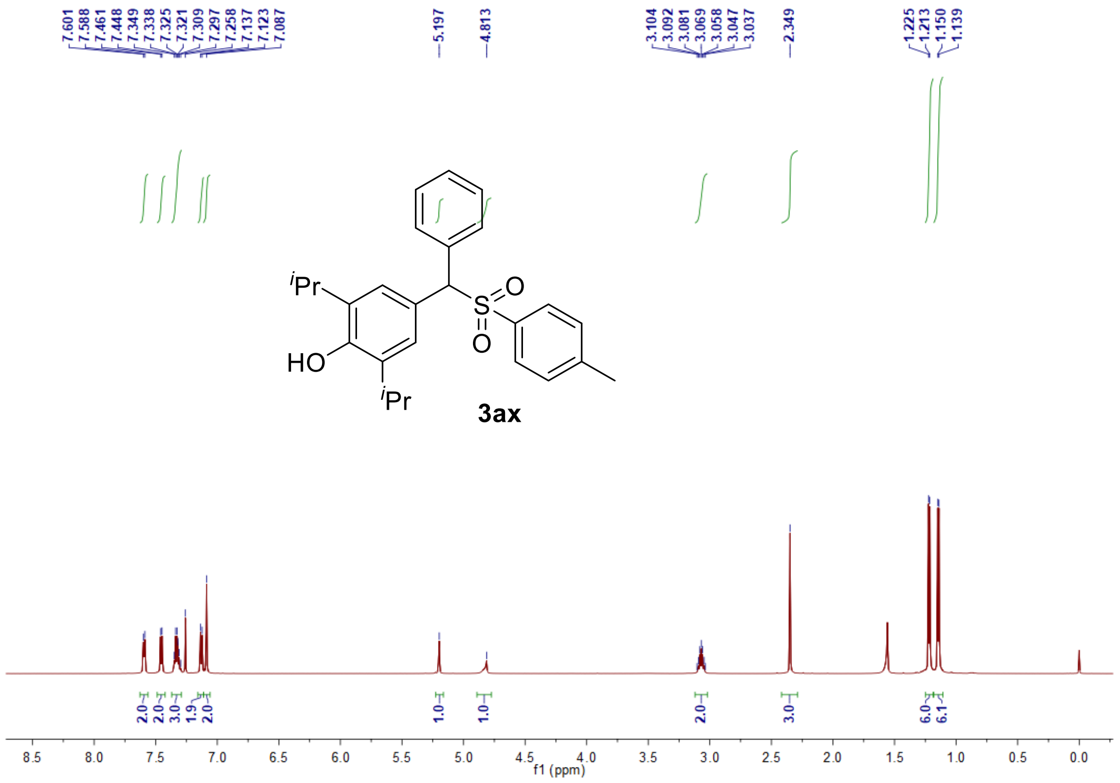

7.601
7.588
7.461
7.448
7.349
7.338
7.325
7.321
7.309
7.297
7.258
7.137
7.123
7.087
5.197
4.813
3.104
3.092
3.081
3.069
3.058
3.047
3.037
2.349
1.225
1.213
1.150
1.139
^{i}Pr
HO
^{i}Pr
S
O
O
3ax
2.0
2.0
3.0
1.9
2.0
1.0
1.0
2.0
3.0
6.0
6.1
f1 (ppm)

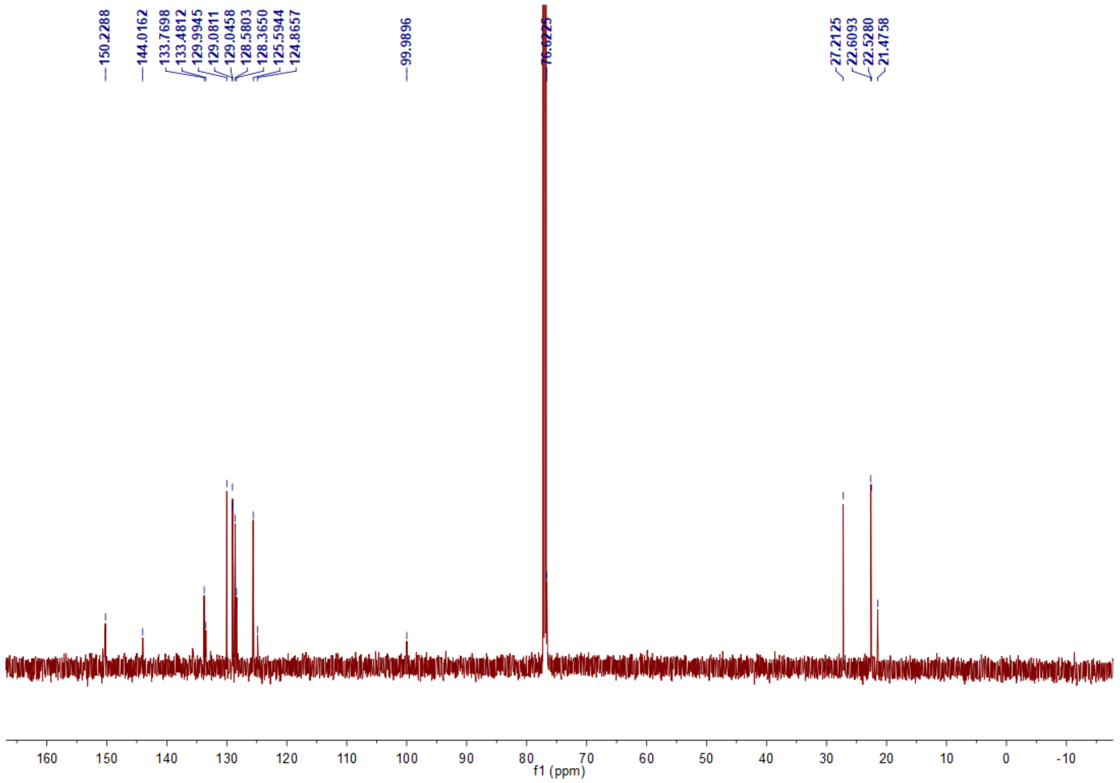

150.2288
144.0162
133.7698
133.4812
129.9945
129.0811
129.0458
128.5803
128.3650
125.5944
124.8657
99.9896
27.2125
22.6093
22.5280
21.4758
f1 (ppm)

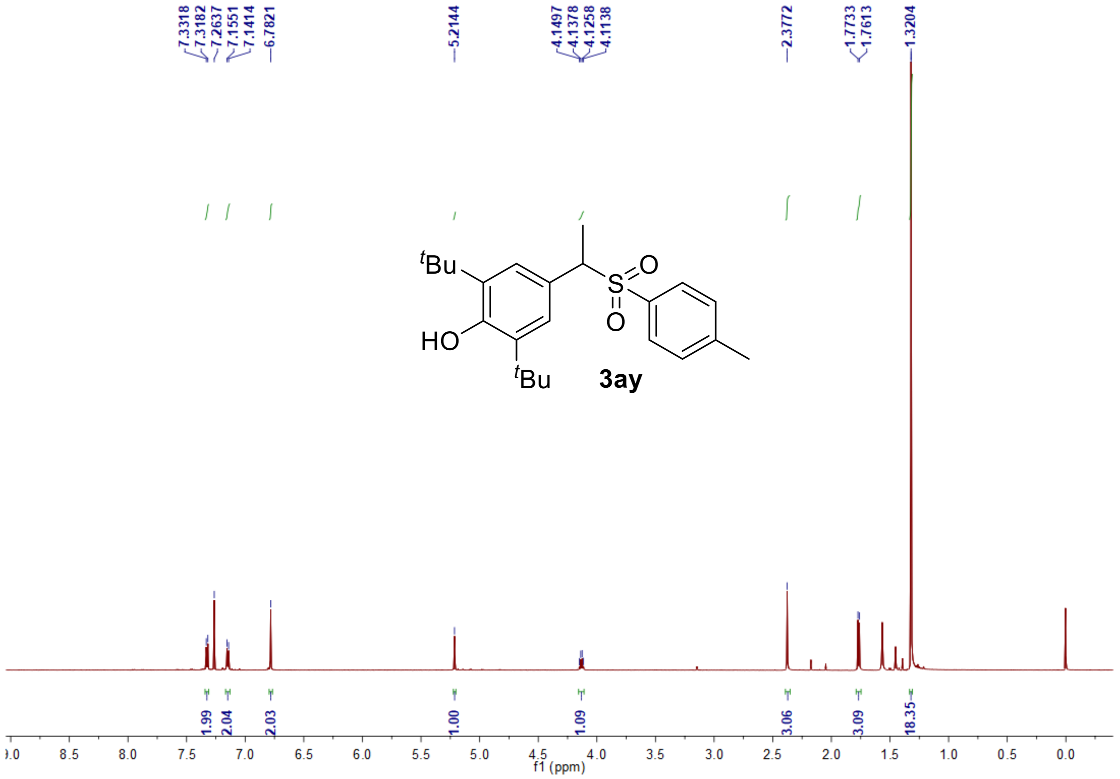
7.3318
7.3182
7.2637
7.1551
7.1414
6.7821
5.2144
4.1497
4.1378
4.1258
4.1138
2.3772
1.7733
1.7613
1.3204
^{t}Bu
HO
^{t}Bu
3ay
1.99
2.04
2.03
1.00
1.09
3.06
3.09
18.35
f1 (ppm)

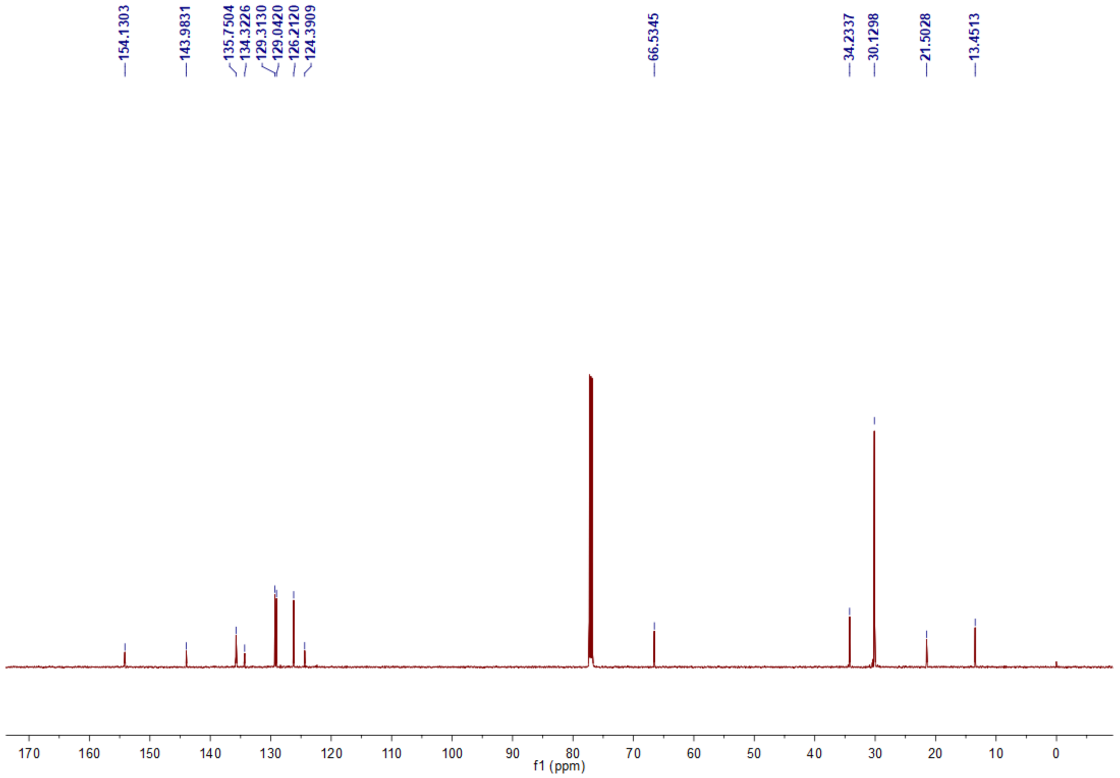
154.1303
143.9831
135.7504
134.3226
129.3130
129.0420
126.2120
124.3909
66.5345
34.2337
30.1298
21.5028
13.4513
f1 (ppm)

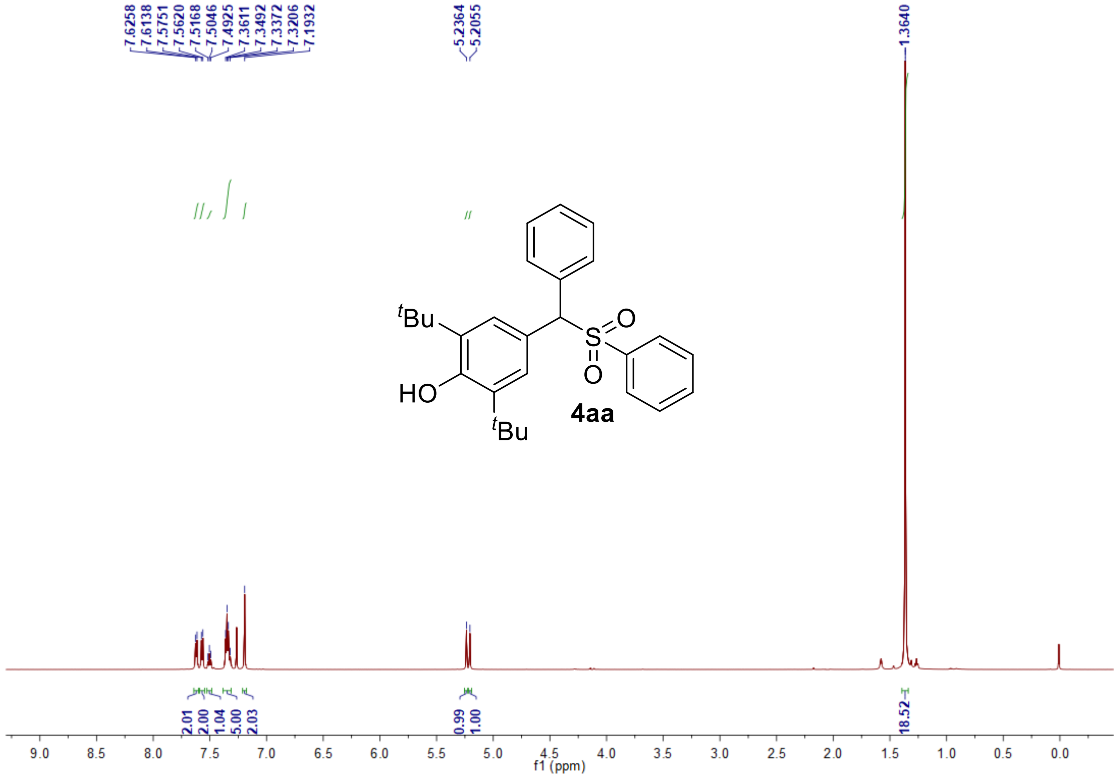
7.6258
7.6138
7.5751
7.5620
7.5168
7.5046
7.4925
7.3611
7.3492
7.3372
7.3206
7.1932
5.2364
5.2055
1.3640
^{t}Bu
HO
^{t}Bu
S
O
O
4aa
2.01
2.00
1.04
5.00
2.03
0.99
1.00
18.52
9.0 8.5 8.0 7.5 7.0 6.5 6.0 5.5 5.0 4.5 4.0 3.5 3.0 2.5 2.0 1.5 1.0 0.5 0.0
f1 (ppm)

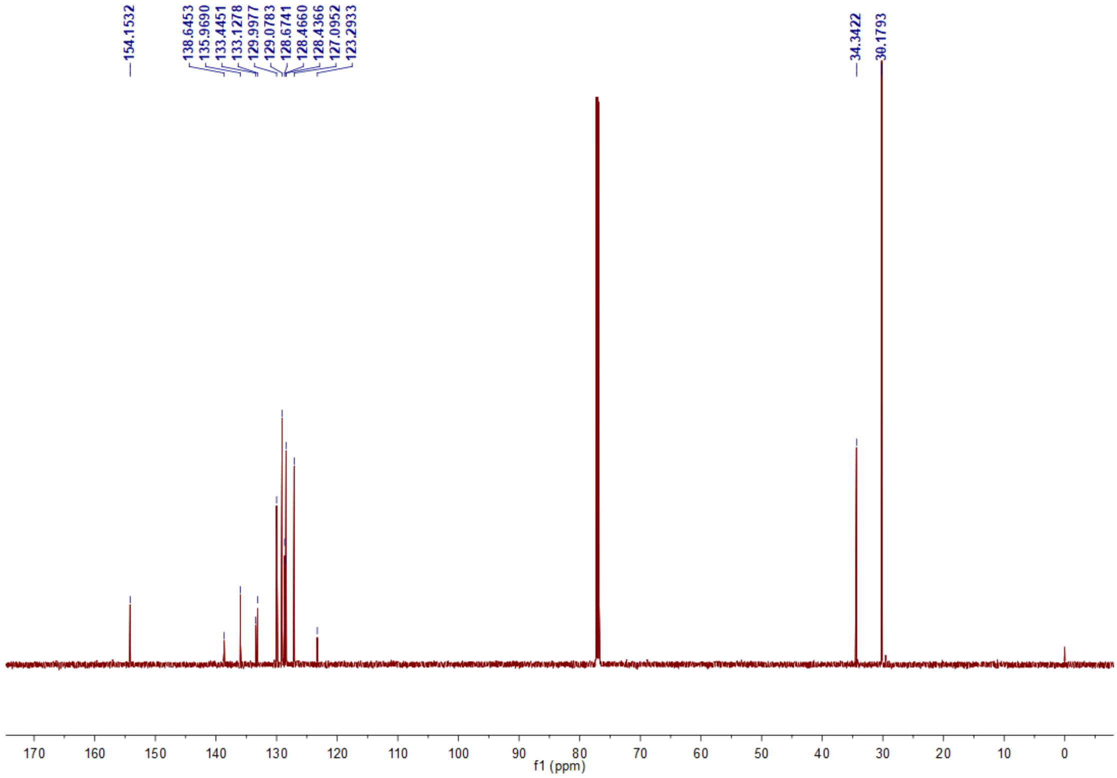
154.1532
138.6453
135.9690
133.4451
133.1278
129.9977
129.0783
128.6741
128.4660
128.4366
127.0952
123.2933
34.3422
30.1793
170 160 150 140 130 120 110 100 90 80 70 60 50 40 30 20 10 0
f1 (ppm)

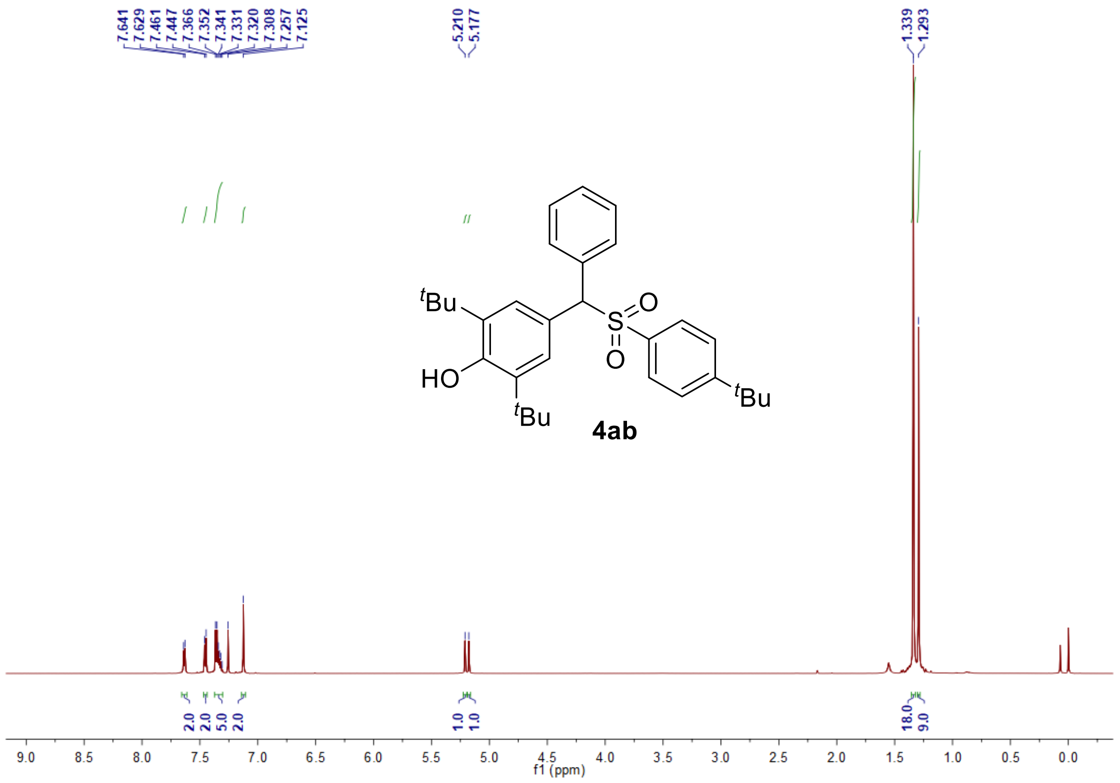
7.641
7.629
7.461
7.447
7.366
7.352
7.341
7.331
7.320
7.308
7.257
7.125
5.210
5.177
1.339
1.293
tBu
HO
tBu
S
O
O
tBu
4ab
2.0
2.0
5.0
2.0
1.0
1.0
18.0
9.0
9.0 8.5 8.0 7.5 7.0 6.5 6.0 5.5 5.0 4.5 4.0 3.5 3.0 2.5 2.0 1.5 1.0 0.5 0.0
f1 (ppm)

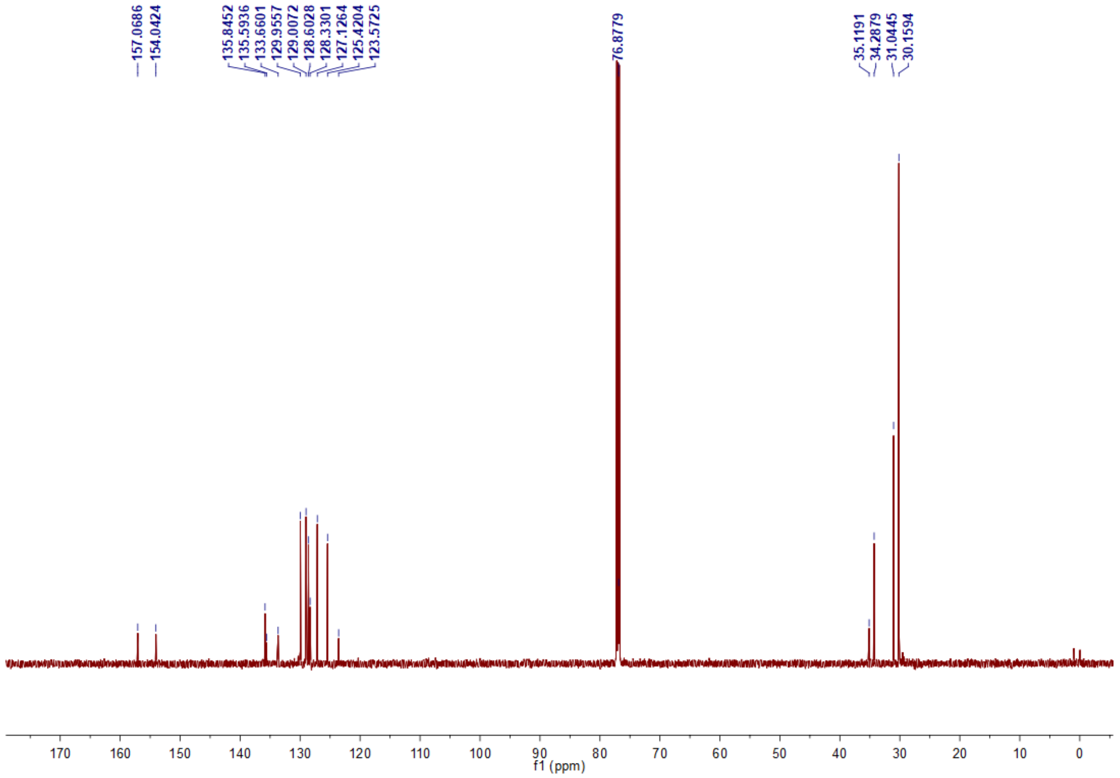
157.0686
154.0424
135.8452
135.5936
133.6601
129.9557
129.0072
128.6028
128.3301
127.1264
125.4204
123.5725
76.8779
35.1191
34.2879
31.0445
30.1594
170 160 150 140 130 120 110 100 90 80 70 60 50 40 30 20 10 0
f1 (ppm)

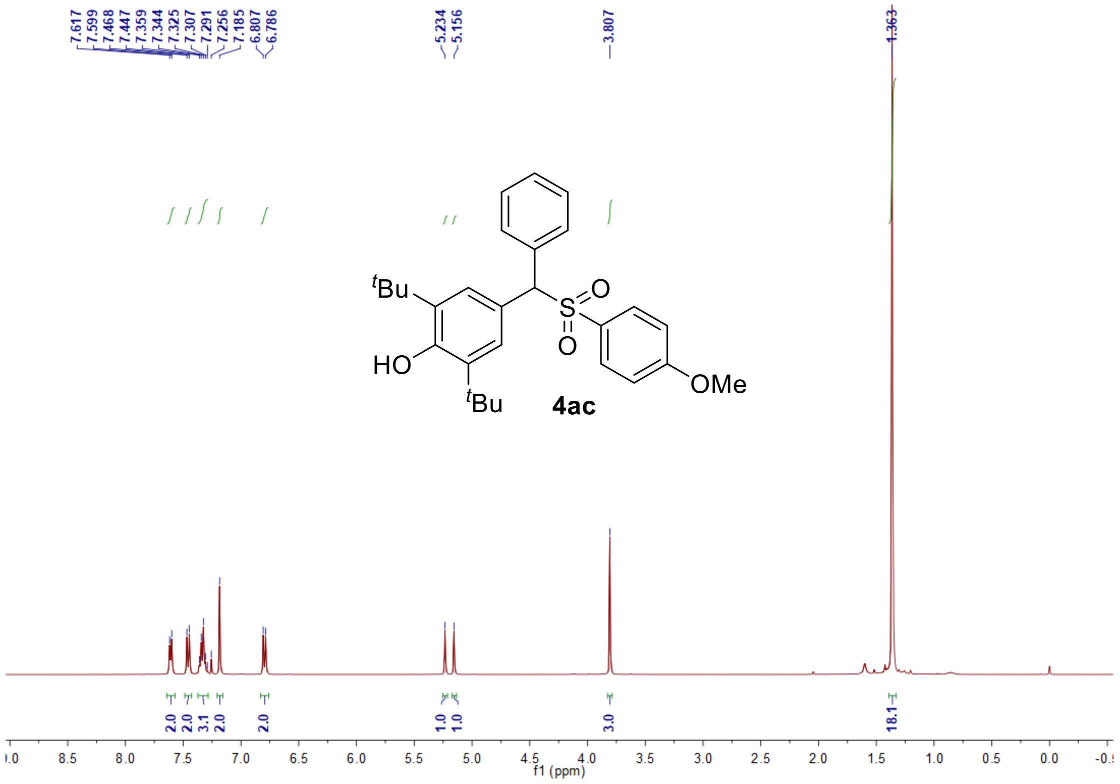
7.617
7.599
7.468
7.447
7.359
7.344
7.325
7.307
7.291
7.256
7.185
6.807
6.786
5.234
5.156
3.807
1.363
tBu
HO
tBu
S
O
O
OMe
4ac
2.0
2.0
3.1
2.0
2.0
1.0
1.0
3.0
18.1
f1 (ppm)

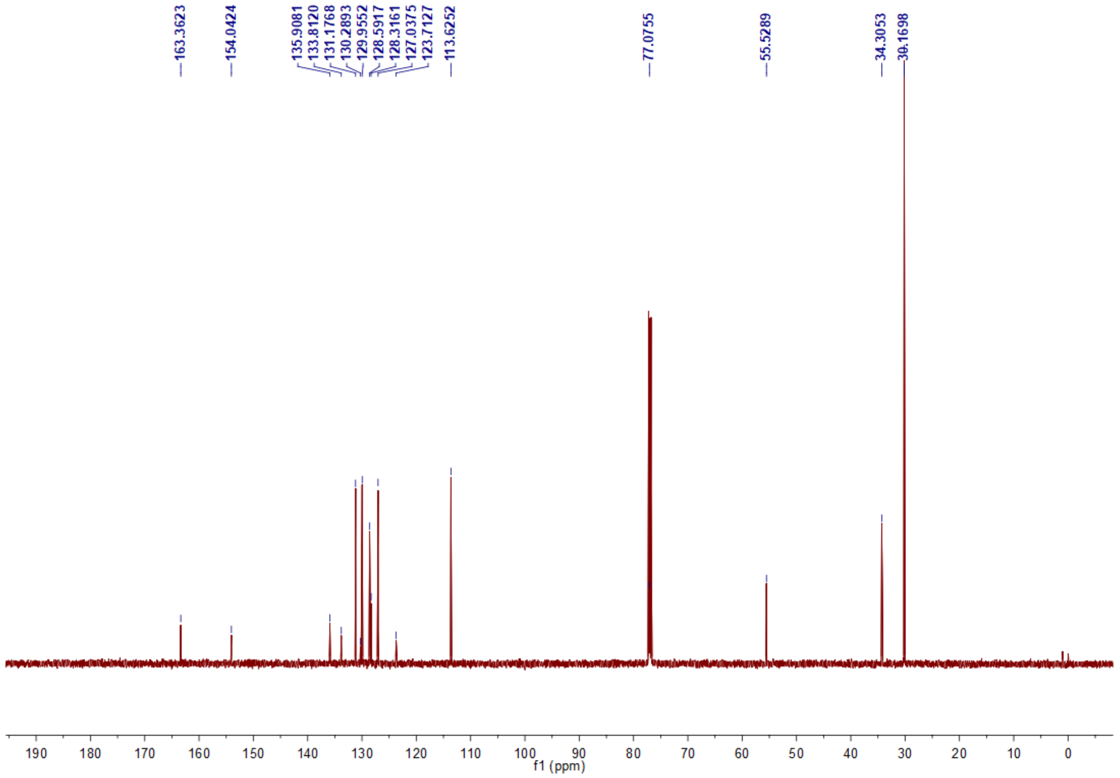
163.3623
154.0424
135.9081
133.8120
131.1768
130.2893
129.9552
128.5917
128.3161
127.0375
123.7127
113.6252
77.0755
55.5289
34.3053
30.1698
f1 (ppm)

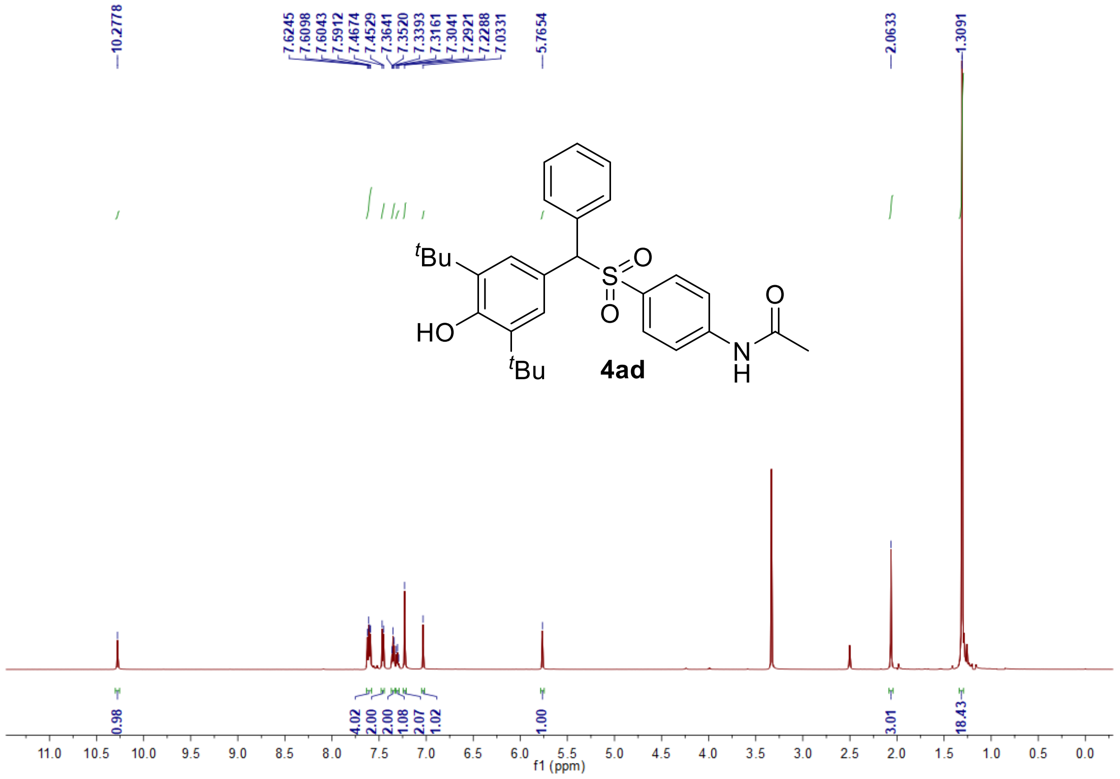
10.2778
7.6245
7.6098
7.6043
7.5912
7.4674
7.4529
7.3641
7.3520
7.3393
7.3161
7.3041
7.2921
7.2288
7.0331
5.7654
2.0633
1.3091
tBu
HO
tBu
4ad
O
S
O
O
N
H
0.98
4.02
2.00
2.00
1.08
2.07
1.02
1.00
3.01
18.43
f1 (ppm)

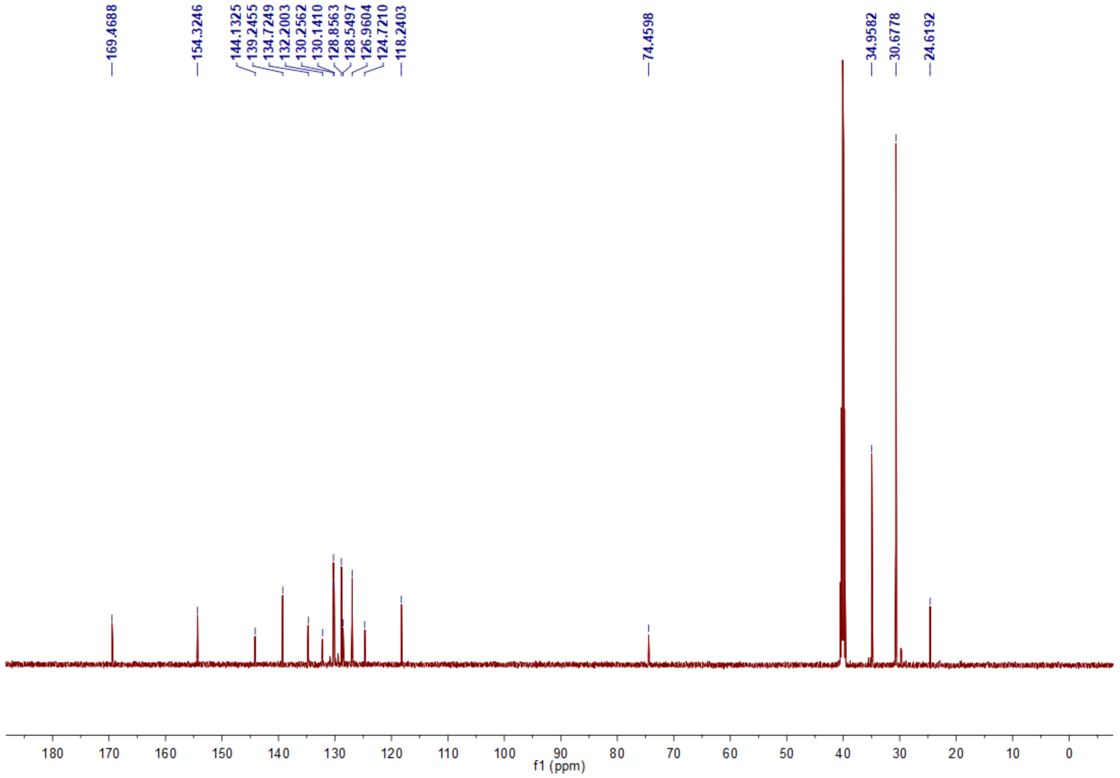
169.4688
154.3246
144.1325
139.2455
134.7249
132.2003
130.2562
130.1410
128.8563
128.5497
126.9604
124.7210
118.2403
74.4598
34.9582
30.6778
24.6192
f1 (ppm)

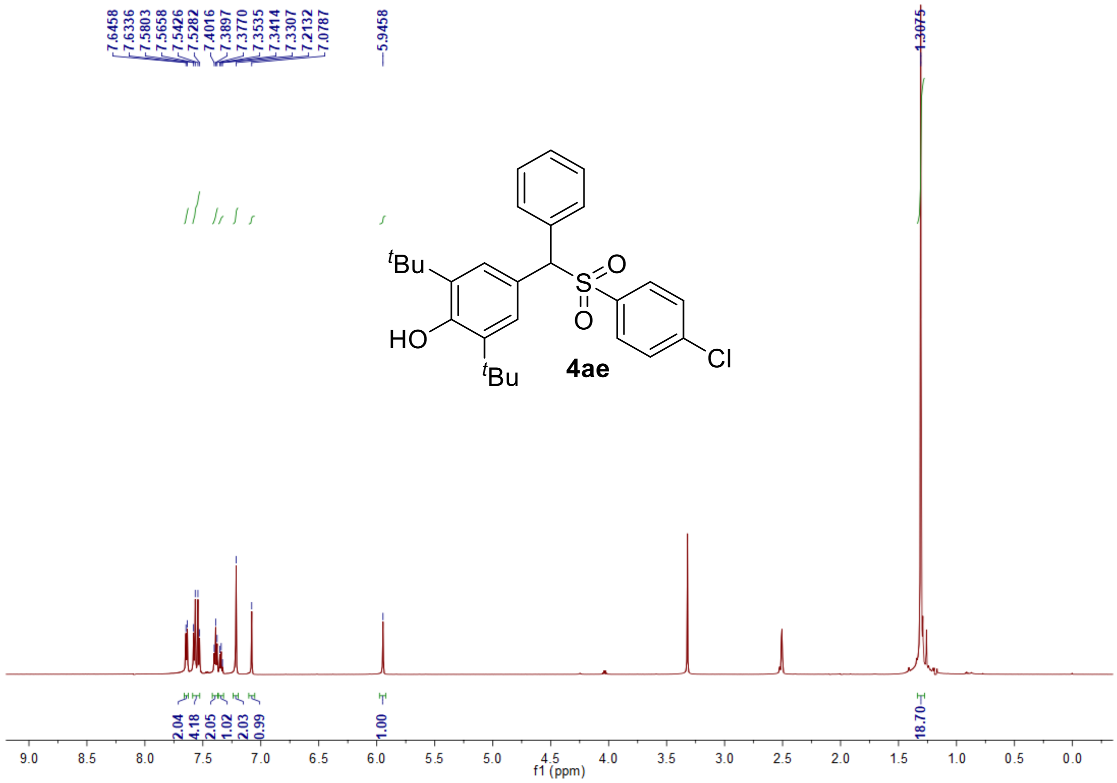
7.6458
7.6336
7.5803
7.5658
7.5426
7.5282
7.4016
7.3897
7.3770
7.3535
7.3414
7.3307
7.2132
7.0787
5.9458
1.3075
tBu
HO
tBu
S
O
O
Cl
4ae
2.04
4.18
2.05
1.02
2.03
0.99
1.00
18.70
f1 (ppm)

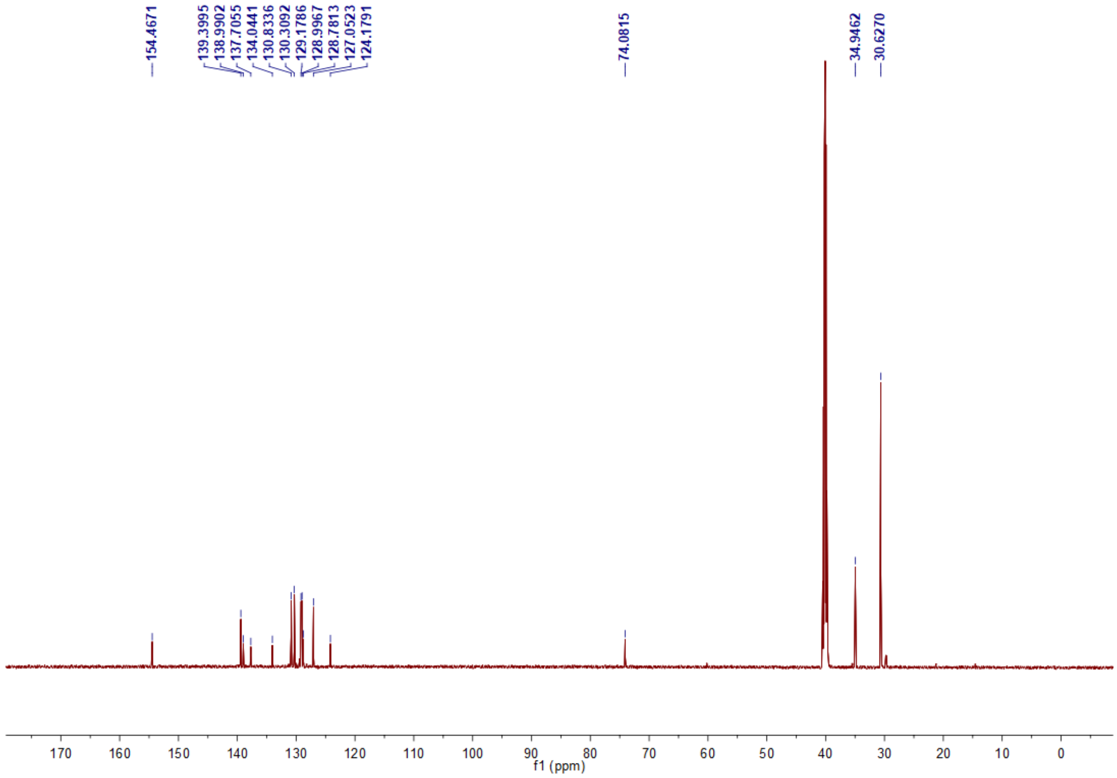
154.4671
139.3995
138.9902
137.7055
134.0441
130.8336
130.3092
129.1786
128.9967
128.7813
127.0523
124.1791
74.0815
34.9462
30.6270
f1 (ppm)

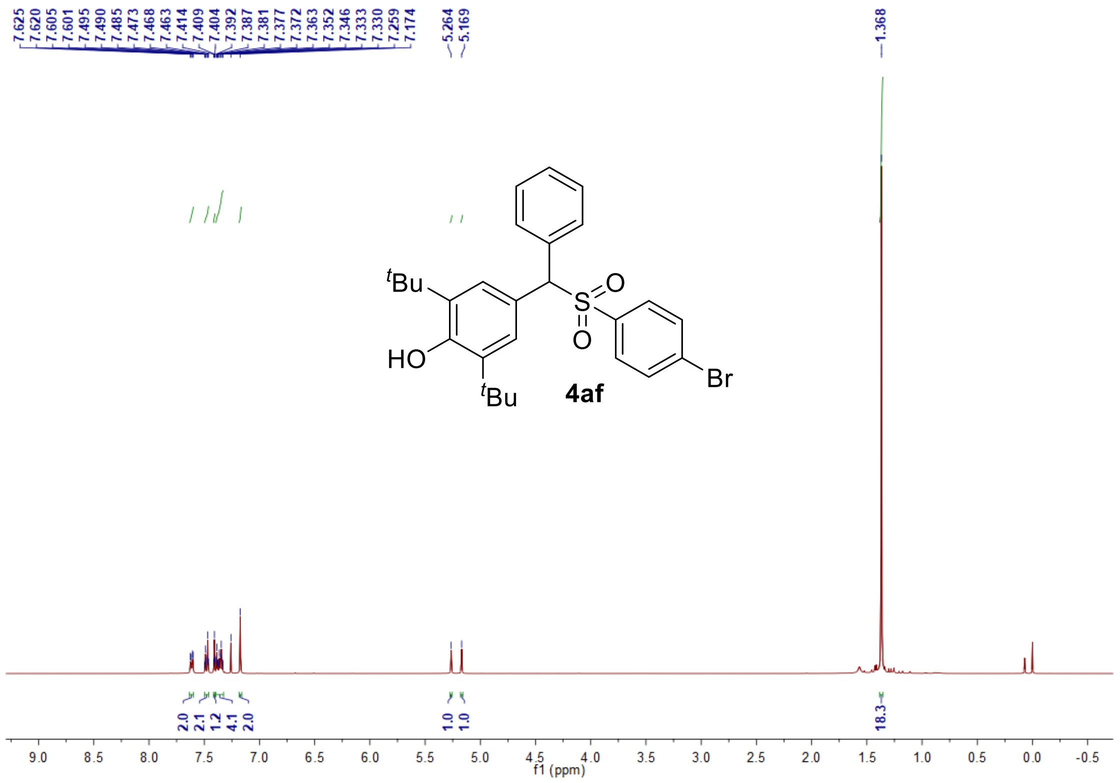

7.625
7.620
7.605
7.601
7.495
7.490
7.485
7.473
7.468
7.463
7.414
7.409
7.404
7.392
7.387
7.381
7.377
7.372
7.363
7.352
7.346
7.333
7.330
7.259
7.174
5.264
5.169
1.368
tBu
HO
tBu
O
S
O
Br
4af
2.0
2.1
1.2
4.1
2.0
1.0
1.0
18.3
f1 (ppm)

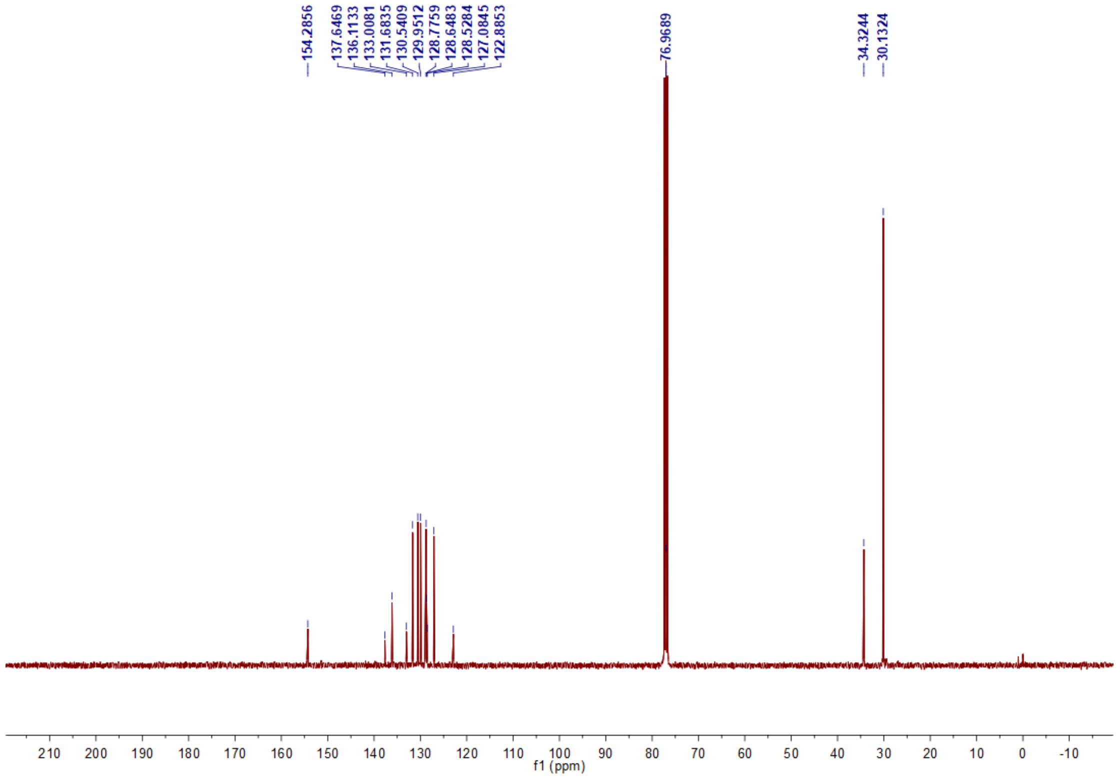

154.2856
137.6469
136.1133
133.0081
131.6835
130.5409
129.9512
128.7759
128.6483
128.5284
127.0845
122.8853
76.9689
34.3244
30.1324
f1 (ppm)

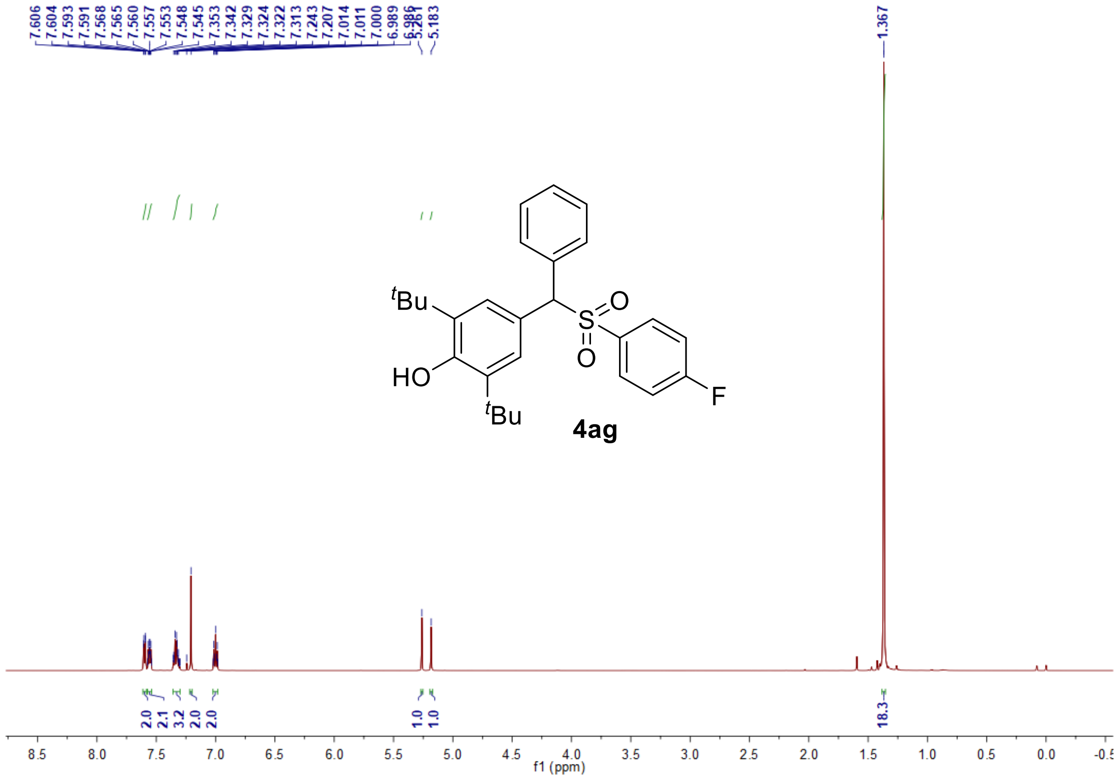
7.606
7.604
7.593
7.591
7.568
7.565
7.560
7.557
7.553
7.548
7.545
7.353
7.342
7.329
7.324
7.322
7.313
7.243
7.207
7.014
7.011
7.000
6.989
6.986
5.261
5.183
1.367
tBu
HO
tBu
S
O
O
F
4ag
2.0
2.1
3.2
2.0
2.0
1.0
1.0
18.3
f1 (ppm)

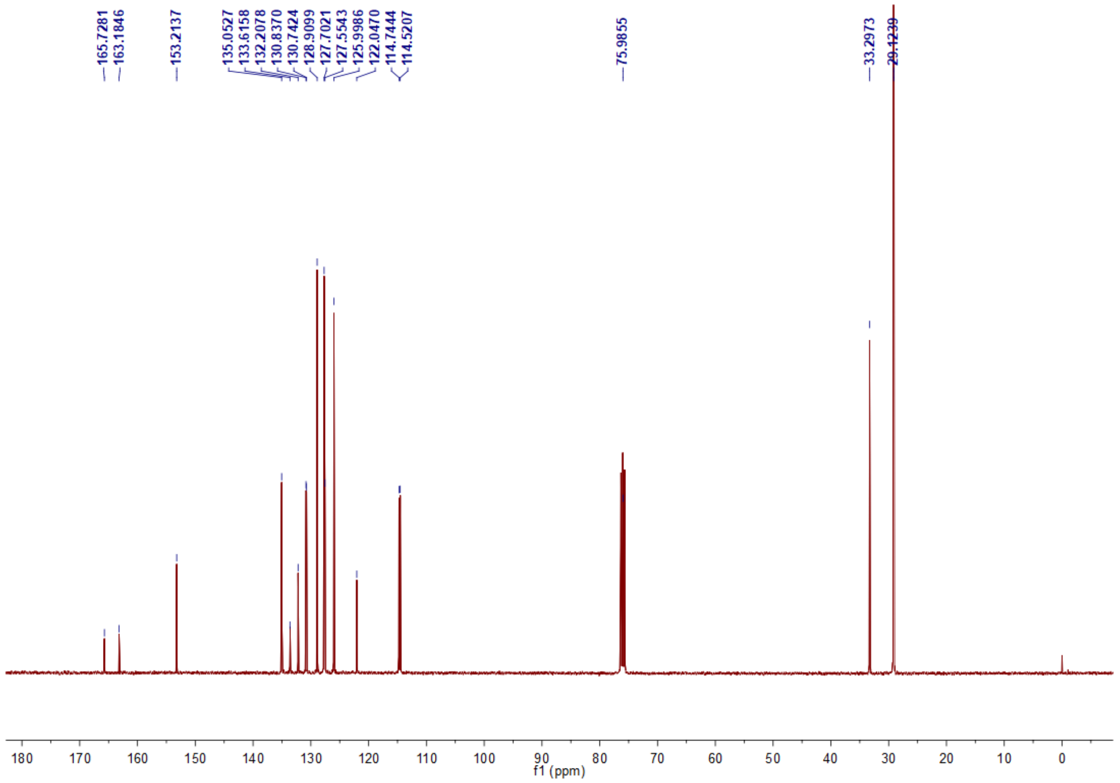
165.7281
163.1846
153.2137
135.0527
133.6158
132.2078
130.8370
130.7424
128.9099
127.7021
127.5543
125.9986
122.0470
114.7444
114.5207
75.9855
33.2973
29.1239
f1 (ppm)

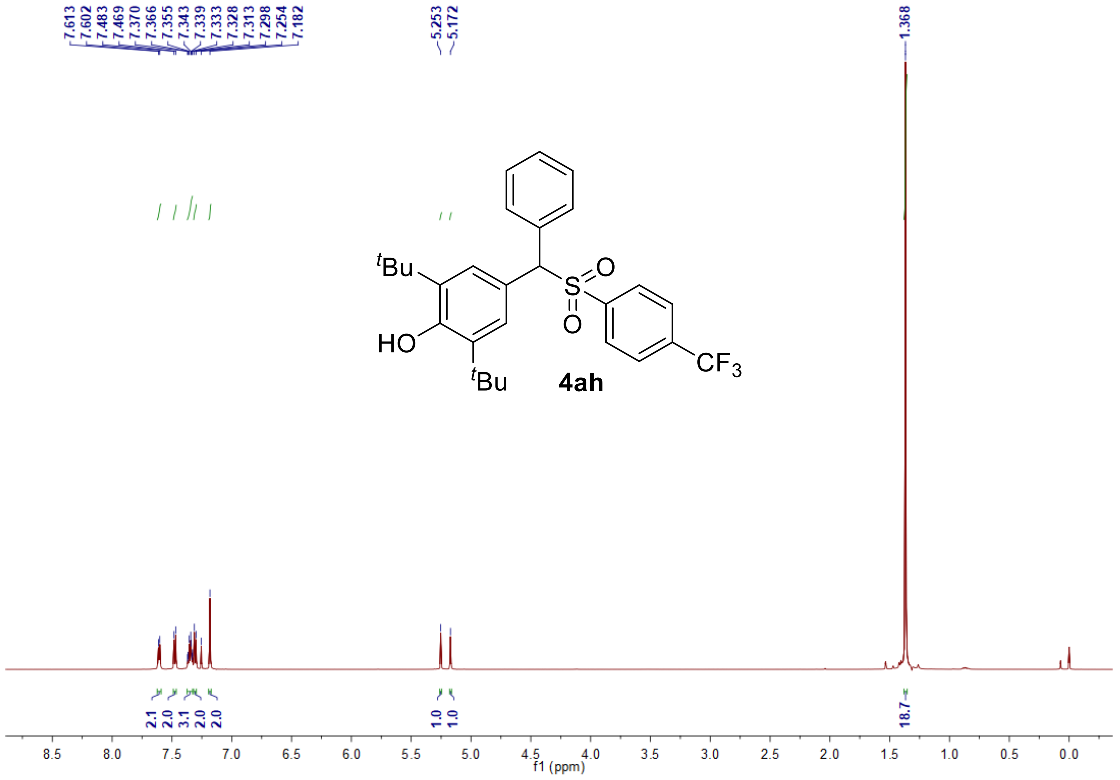
7.613
7.602
7.483
7.469
7.370
7.366
7.355
7.343
7.339
7.333
7.328
7.313
7.298
7.254
7.182
5.253
5.172
1.368
tBu
HO
tBu
O
S
O
CF3
4ah
2.1
2.0
3.1
2.0
2.0
1.0
1.0
18.7
8.5 8.0 7.5 7.0 6.5 6.0 5.5 5.0 4.5 4.0 3.5 3.0 2.5 2.0 1.5 1.0 0.5 0.0
f1 (ppm)

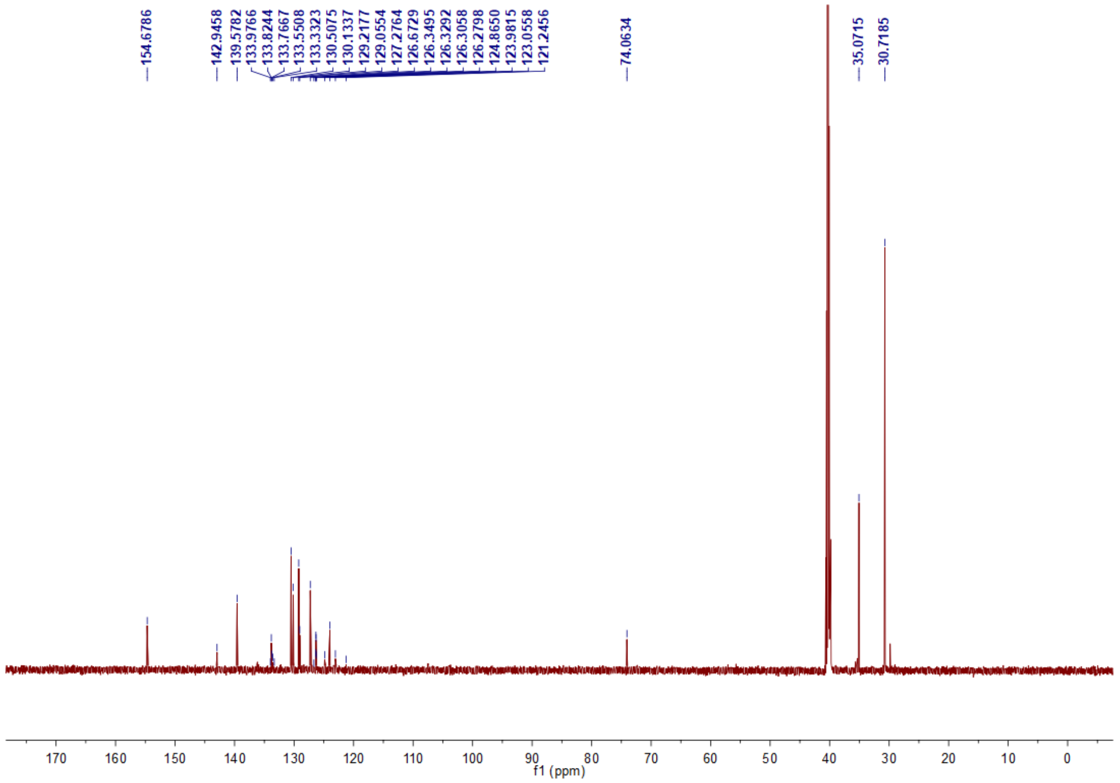
154.6786
142.9458
139.5782
133.9766
133.8244
133.7667
133.5508
133.3323
130.5075
130.1337
129.2177
129.0554
127.2764
126.6729
126.3495
126.3292
126.3058
126.2798
124.8650
123.9815
123.0558
121.2456
74.0634
35.0715
30.7185
170 160 150 140 130 120 110 100 90 80 70 60 50 40 30 20 10 0
f1 (ppm)

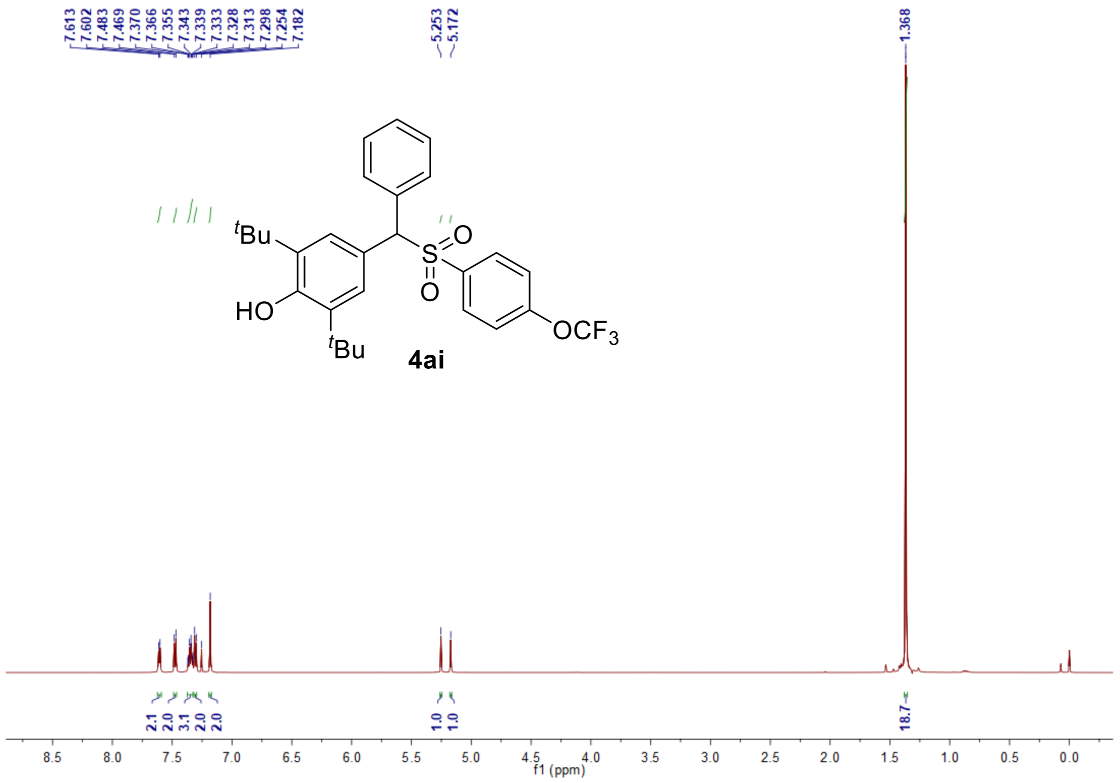
7.613
7.602
7.483
7.469
7.370
7.366
7.355
7.343
7.339
7.333
7.328
7.313
7.298
7.254
7.182
5.253
5.172
1.368
tBu
HO
tBu
O
S
O
OCF3
4ai
2.1
2.0
3.1
2.0
2.0
1.0
1.0
18.7
8.5 8.0 7.5 7.0 6.5 6.0 5.5 5.0 4.5 4.0 3.5 3.0 2.5 2.0 1.5 1.0 0.5 0.0
f1 (ppm)

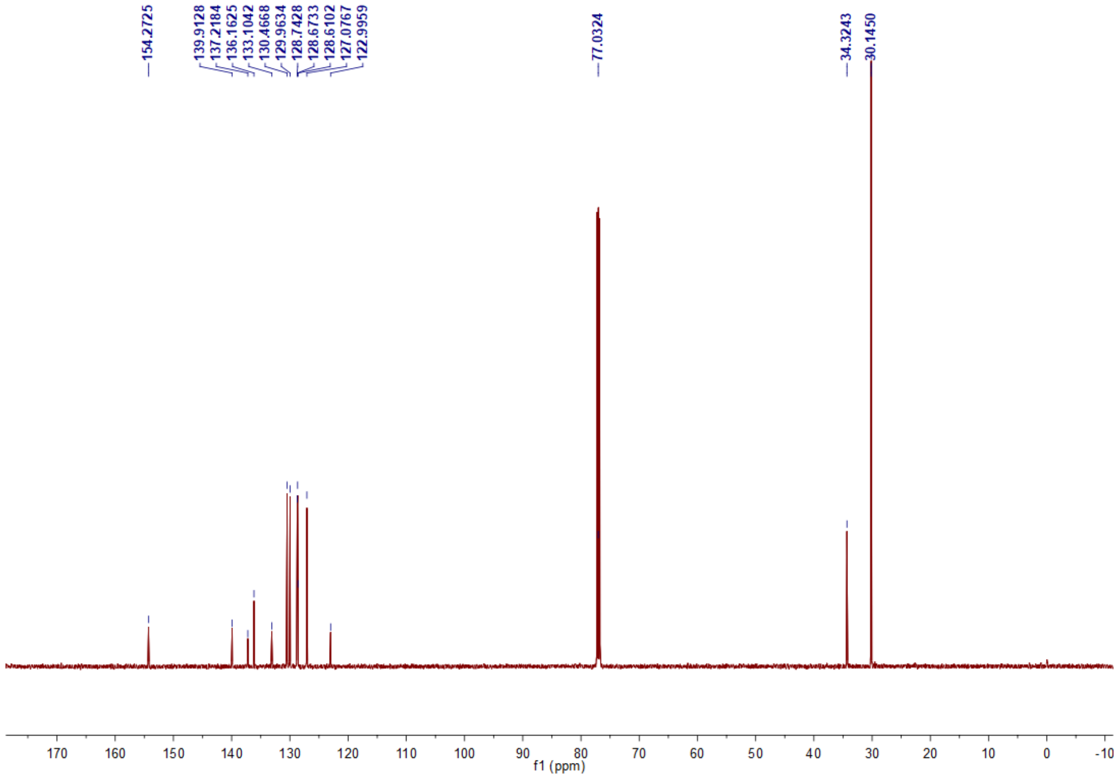
154.2725
139.9128
137.2184
136.1625
133.1042
130.4668
129.9634
128.7428
128.6733
128.6102
127.0767
122.9959
77.0324
34.3243
30.1450
170 160 150 140 130 120 110 100 90 80 70 60 50 40 30 20 10 0 -10
f1 (ppm)

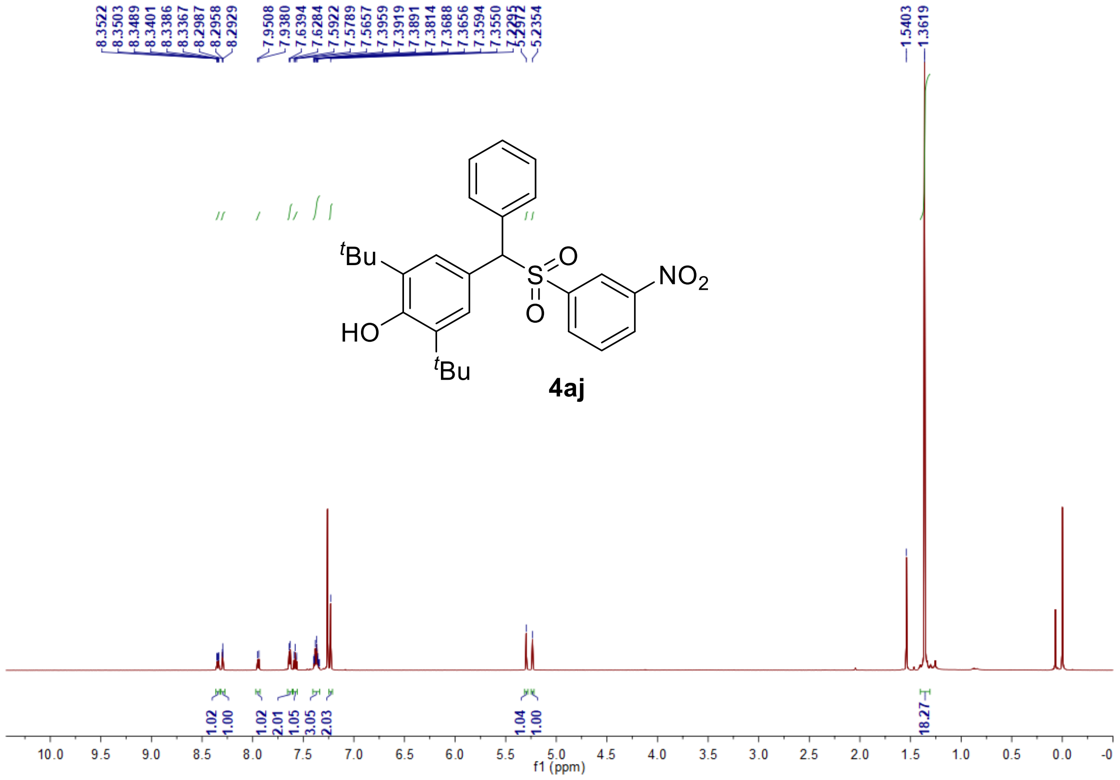

8.3522
8.3503
8.3489
8.3401
8.3386
8.3367
8.2987
8.2958
8.2929
7.9508
7.9380
7.6394
7.6284
7.5922
7.5789
7.5657
7.3959
7.3919
7.3891
7.3814
7.3688
7.3656
7.3594
7.3550
5.2354
1.5403
1.3619
^{t}Bu
HO
^{t}Bu
S
O
O
NO_2
4aj
1.02
1.00
1.02
2.01
1.05
3.05
2.03
1.04
1.00
18.27
f1 (ppm)

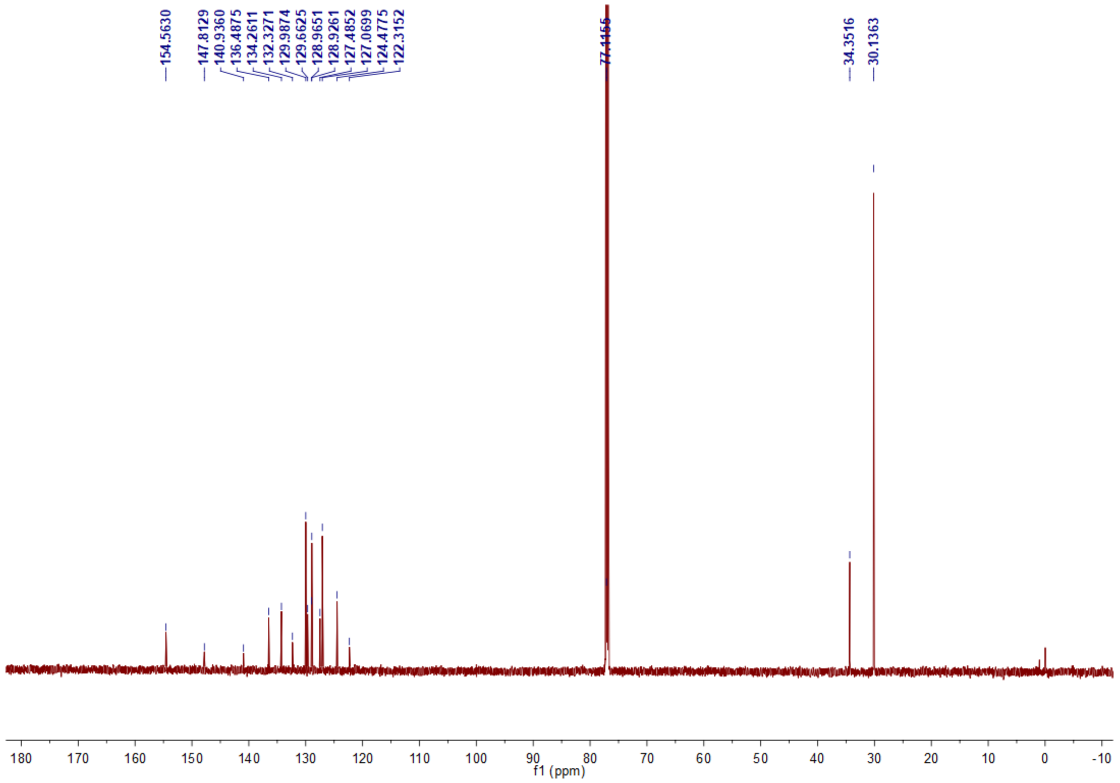

154.5630
147.8129
140.9360
136.4875
134.2611
132.3271
129.9874
129.6625
128.9651
128.9261
127.4852
127.0699
124.4775
122.3152
77.4166
34.3516
30.1363
f1 (ppm)

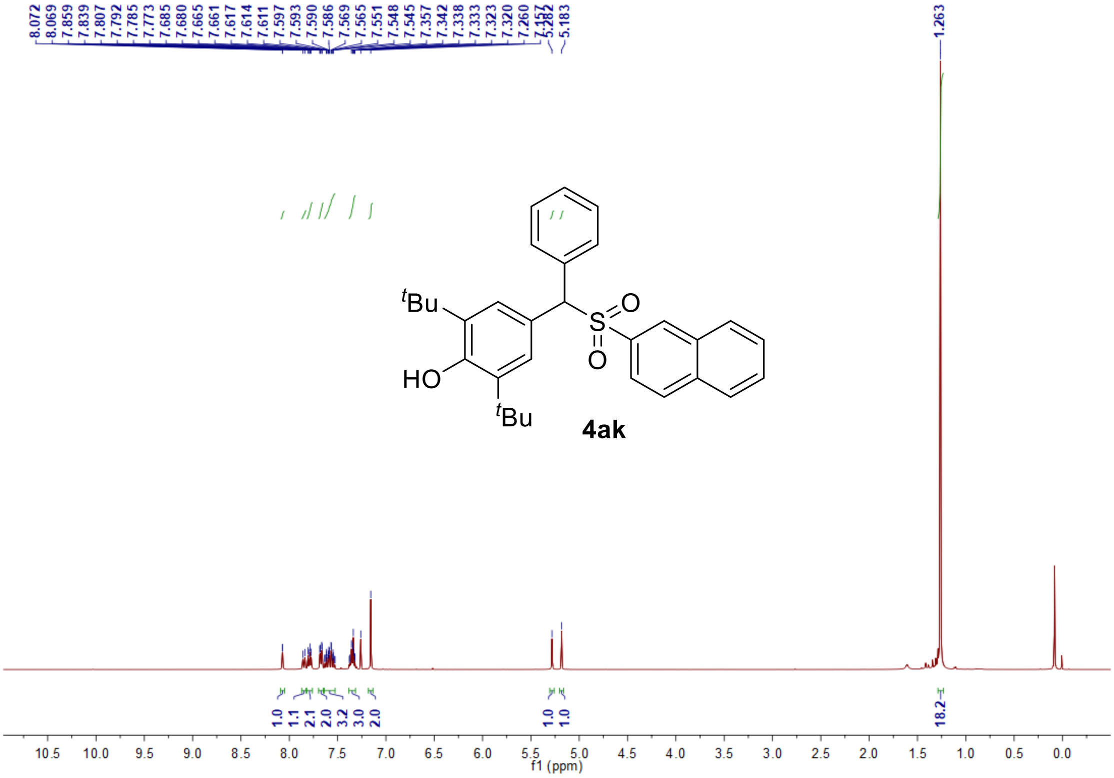
8.072
8.069
7.859
7.839
7.807
7.792
7.785
7.773
7.685
7.680
7.665
7.661
7.617
7.614
7.611
7.597
7.593
7.590
7.586
7.569
7.565
7.551
7.548
7.545
7.357
7.342
7.338
7.333
7.323
7.320
7.260
7.157
5.282
5.183
1.263
tBu
HO
tBu
S
O
O
4ak
1.0
1.1
2.1
2.0
3.2
3.0
2.0
1.0
1.0
18.2
f1 (ppm)

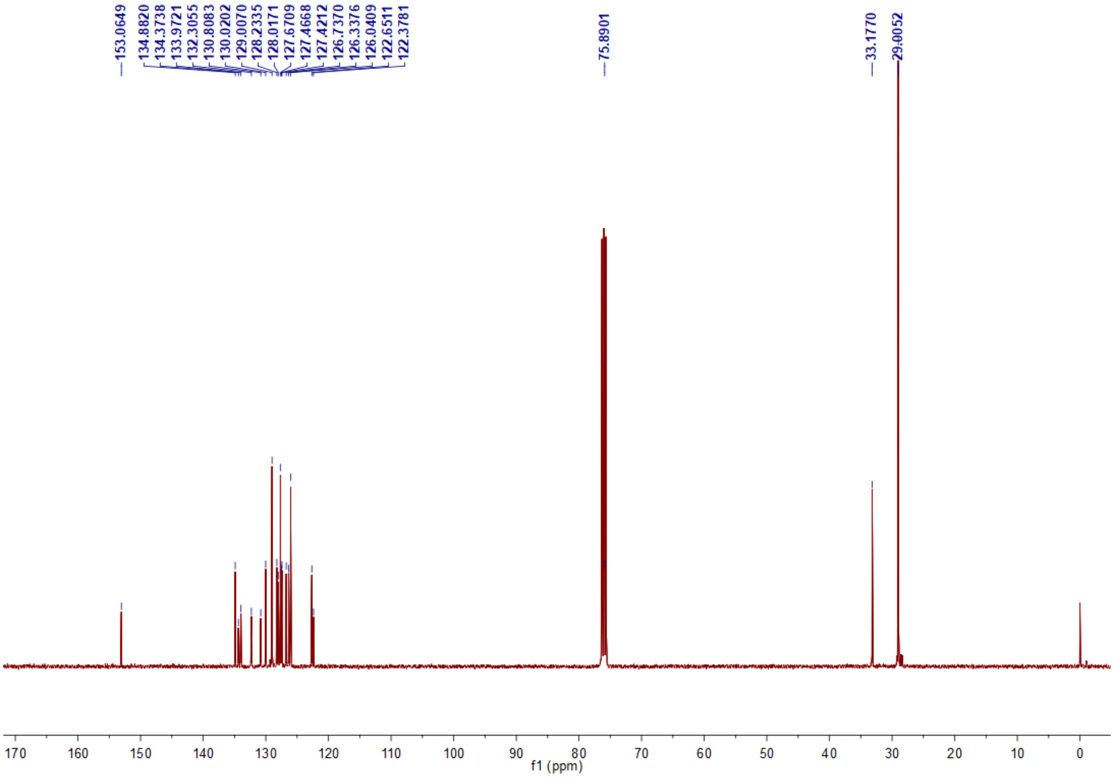
153.0649
134.8820
134.3738
133.9721
132.3055
130.8083
130.0202
129.0070
128.2335
128.0171
127.6709
127.4668
127.4212
126.7370
126.3376
126.0409
122.6511
122.3781
75.8901
33.1770
29.0052
f1 (ppm)

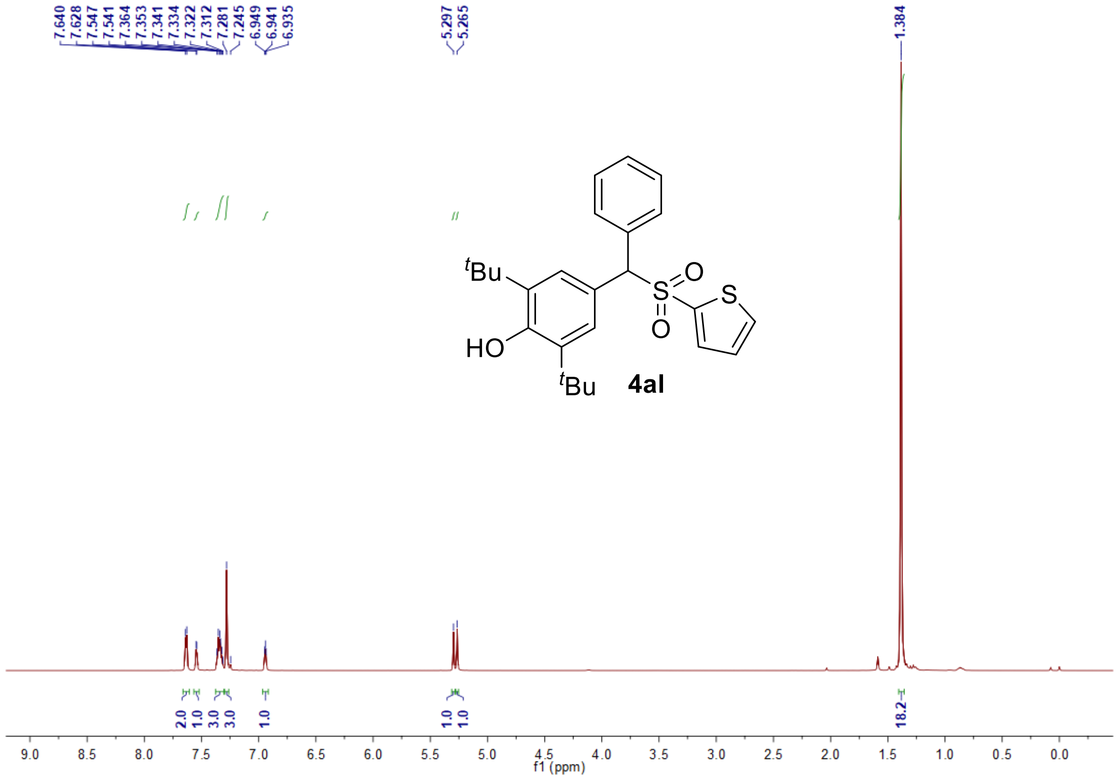
7.640
7.628
7.547
7.541
7.364
7.353
7.341
7.334
7.322
7.312
7.281
7.245
6.949
6.941
6.935
5.297
5.265
1.384
^{t}Bu
HO
^{t}Bu
S
O
O
S
4al
2.0
1.0
3.0
3.0
1.0
1.0
1.0
18.2
9.0 8.5 8.0 7.5 7.0 6.5 6.0 5.5 5.0 4.5 4.0 3.5 3.0 2.5 2.0 1.5 1.0 0.5 0.0
f1 (ppm)

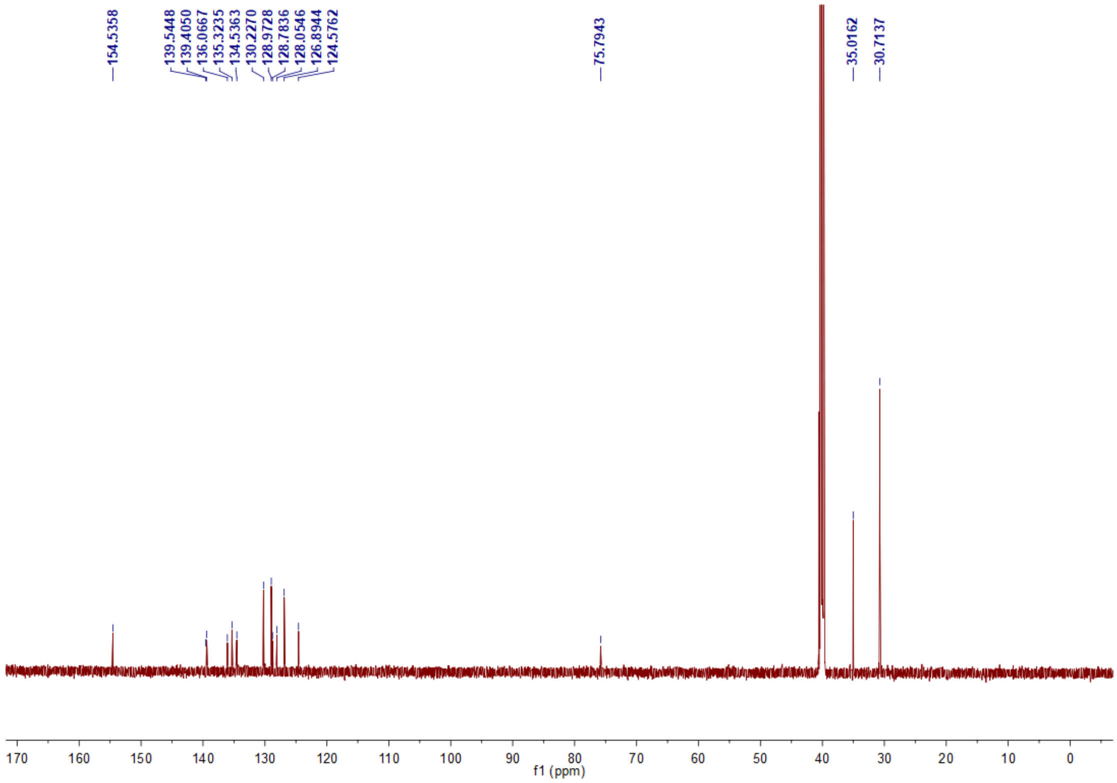
154.5358
139.5448
139.4050
136.0667
135.3235
134.5363
130.2270
128.9728
128.7836
128.0546
126.8944
124.5762
75.7943
35.0162
30.7137
170 160 150 140 130 120 110 100 90 80 70 60 50 40 30 20 10 0
f1 (ppm)

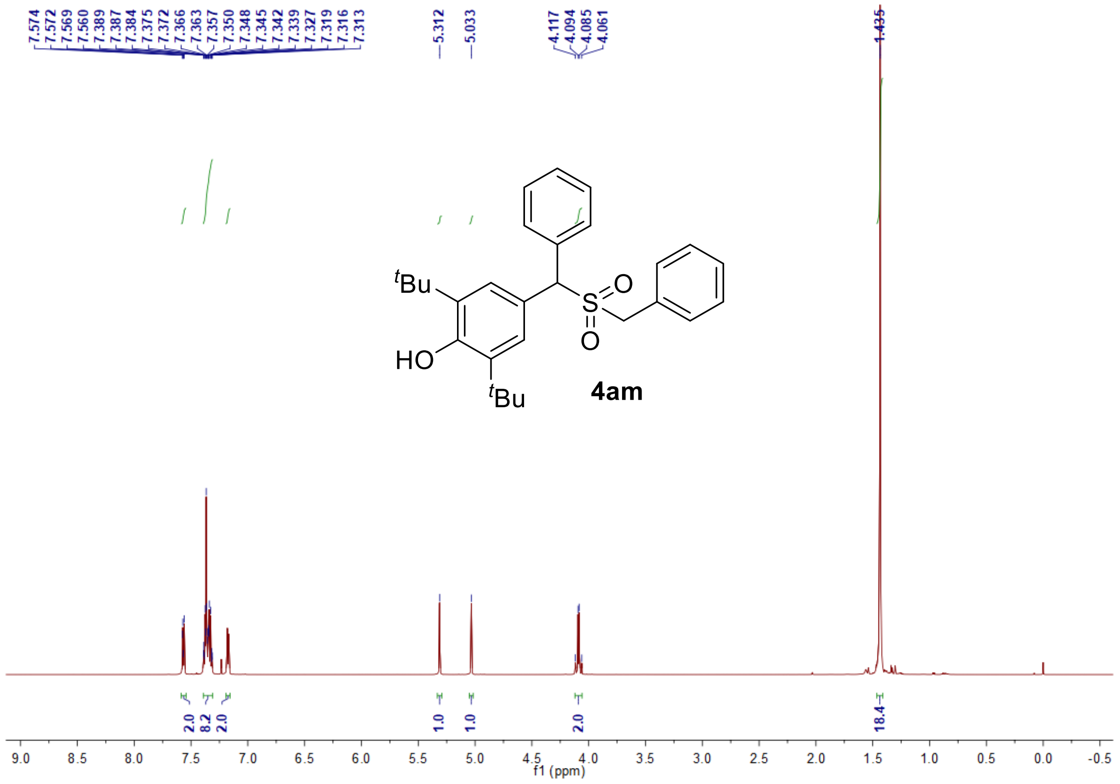
7.574
7.572
7.569
7.560
7.389
7.387
7.384
7.375
7.372
7.366
7.363
7.357
7.350
7.348
7.345
7.342
7.339
7.327
7.319
7.316
7.313
5.312
5.033
4.117
4.094
4.085
4.061
1.435
^{t}Bu
HO
^{t}Bu
O
S
O
4am
2.0
8.2
2.0
1.0
1.0
2.0
18.4
9.0 8.5 8.0 7.5 7.0 6.5 6.0 5.5 5.0 4.5 4.0 3.5 3.0 2.5 2.0 1.5 1.0 0.5 0.0 -0.5
f1 (ppm)

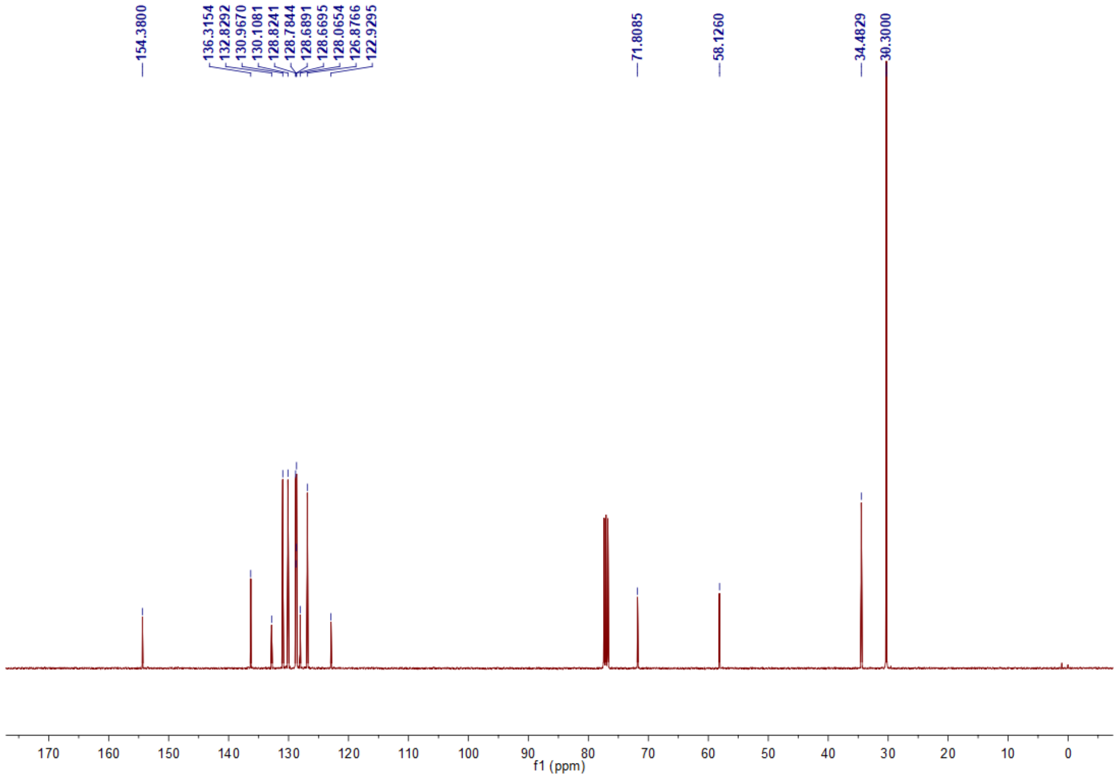
154.3800
136.3154
132.8292
130.9670
130.1081
128.8241
128.7844
128.6891
128.6695
128.0654
126.8766
122.9295
71.8085
58.1260
34.4829
30.3000
170 160 150 140 130 120 110 100 90 80 70 60 50 40 30 20 10 0
f1 (ppm)

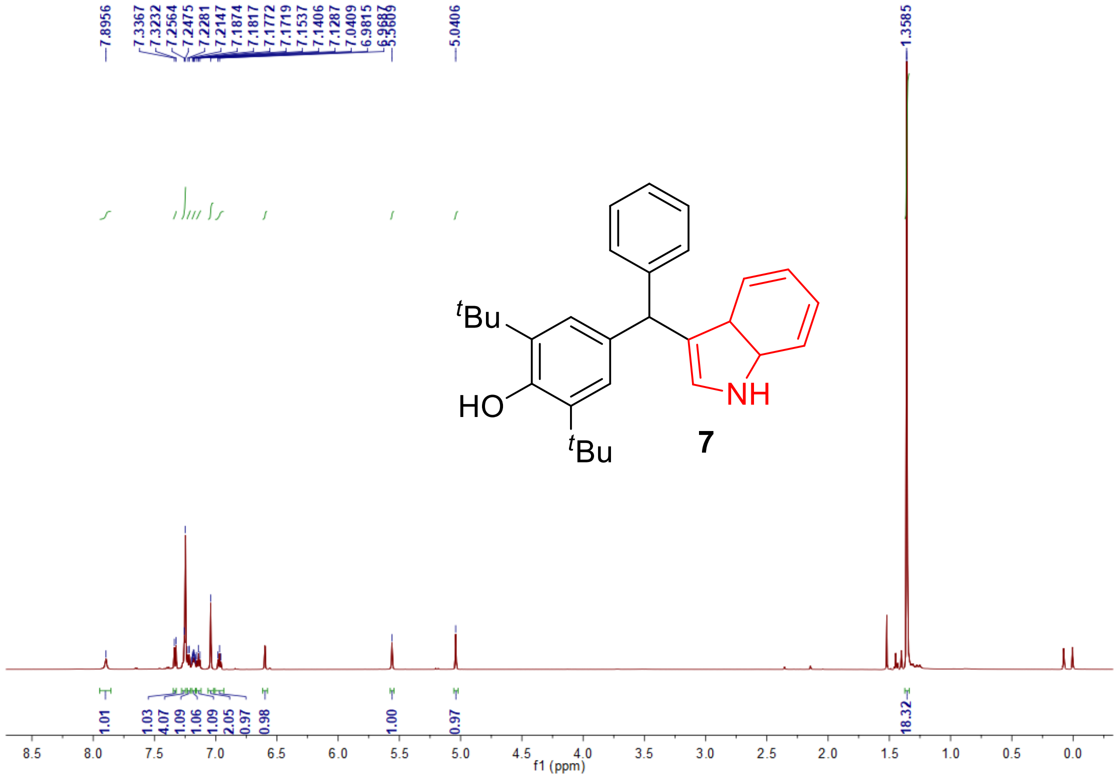
7.8956
7.3367
7.3232
7.2564
7.2475
7.2281
7.2147
7.1874
7.1817
7.1772
7.1719
7.1537
7.1406
7.1287
7.0409
6.9815
5.0406
1.3585
tBu
HO
tBu
NH
7
1.01
1.03
4.07
1.09
1.06
1.09
2.05
0.97
0.98
1.00
0.97
18.32
f1 (ppm)

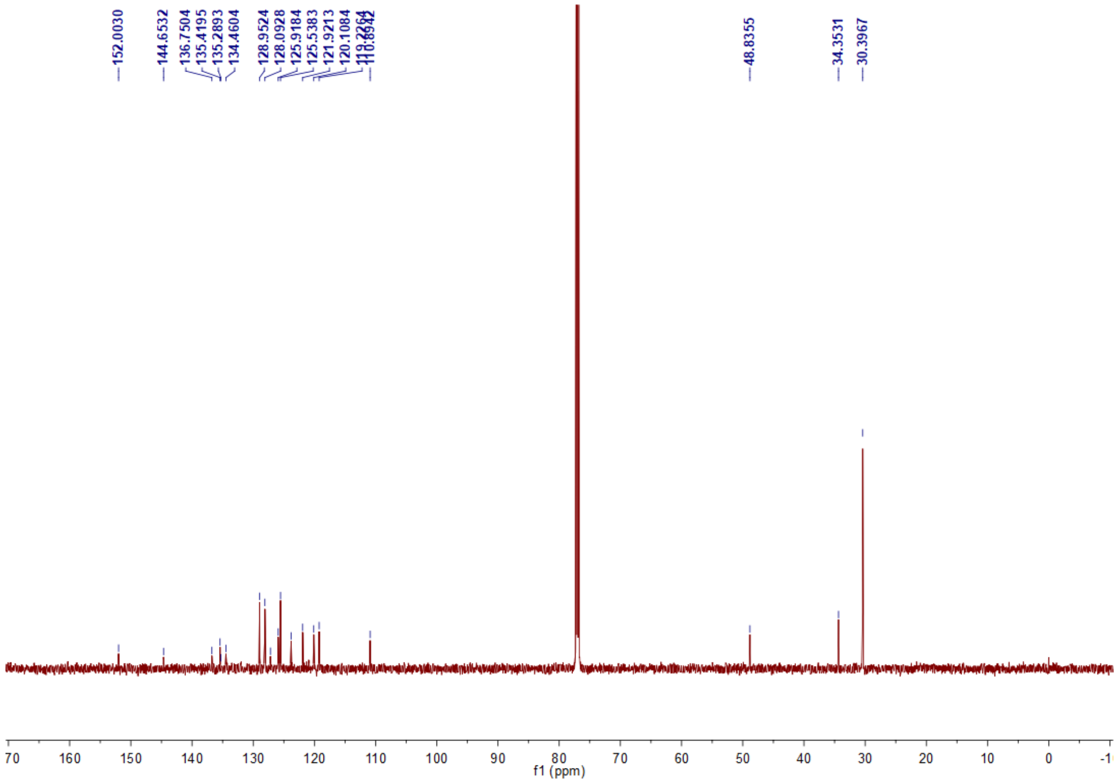
152.0030
144.6532
136.7504
135.4195
135.2893
134.4604
128.9524
128.0928
125.9184
125.5383
121.9213
120.1084
110.8942
48.8355
34.3531
30.3967
f1 (ppm)

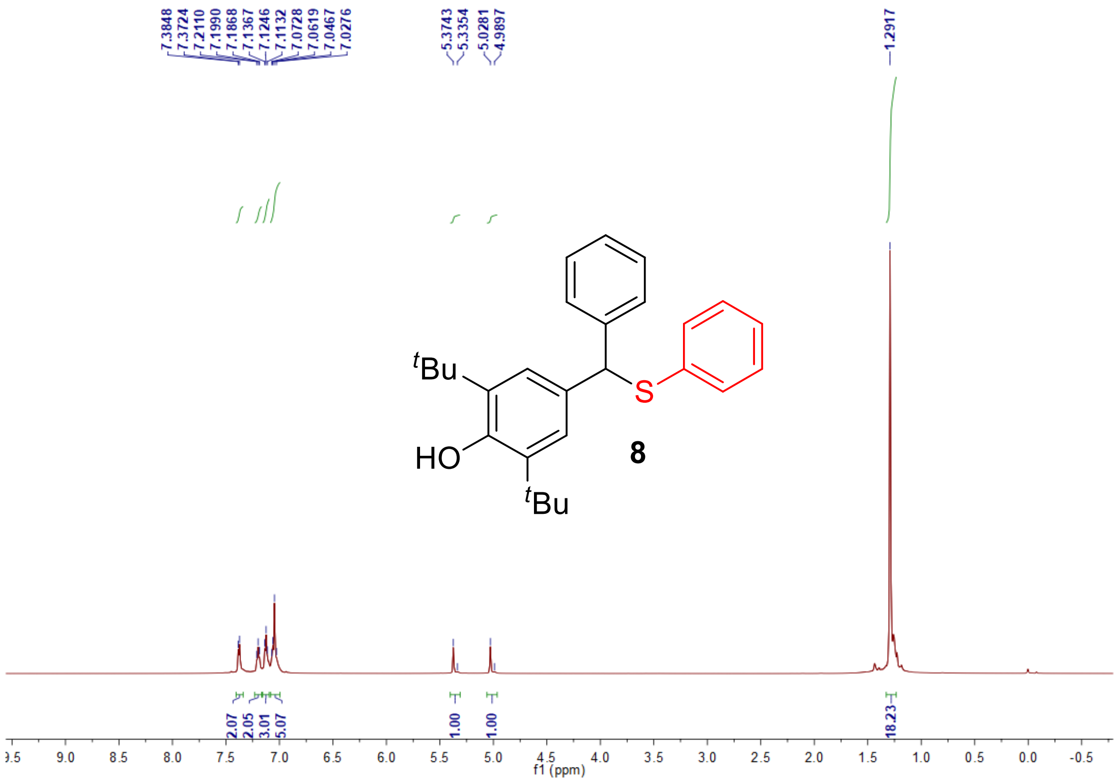

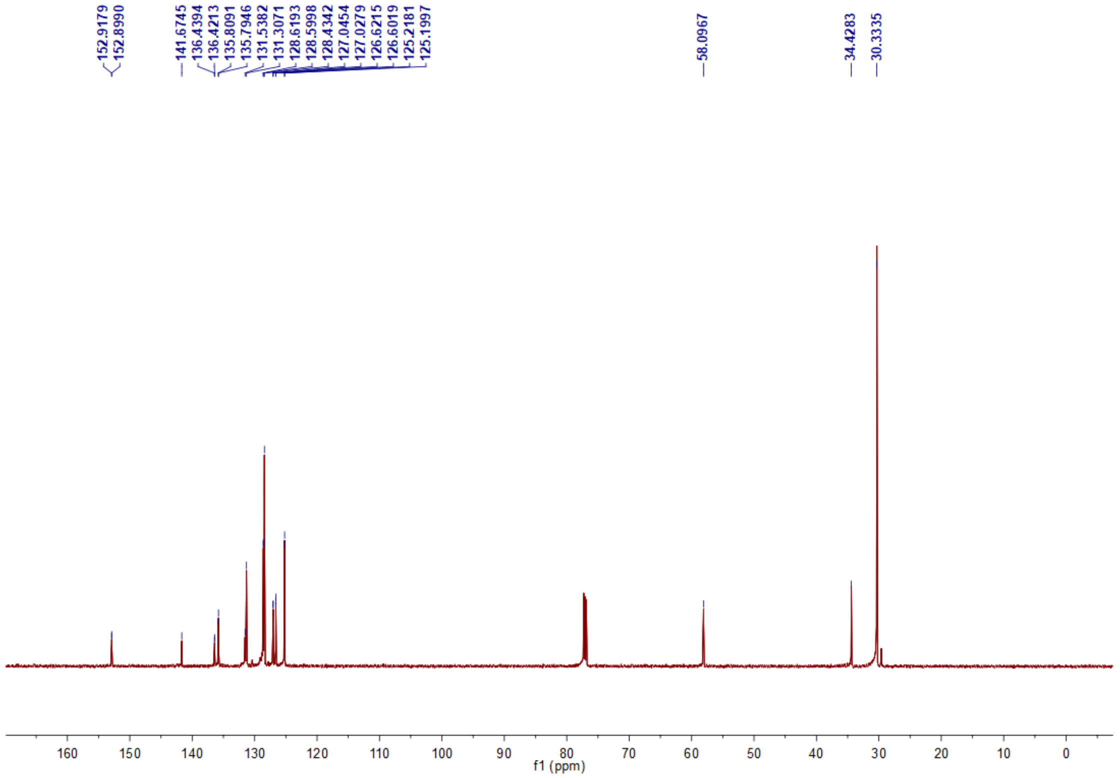

5. HRMS of compounds

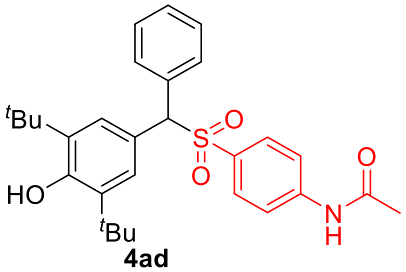

4ad

Acquisition Parameter

Source Type	ESI	Ion Polarity	Positive	Set Nebulizer	0.4 Bar
Focus	Not active	Set Capillary	4500 V	Set Dry Heater	180 °C
Scan Begin	50 m/z	Set End Plate Offset	-500 V	Set Dry Gas	4.0 l/min
Scan End	1300 m/z	Set Charging Voltage	2000 V	Set Divert Valve	Source
		Set Corona	0 nA	Set APCI Heater	0 °C

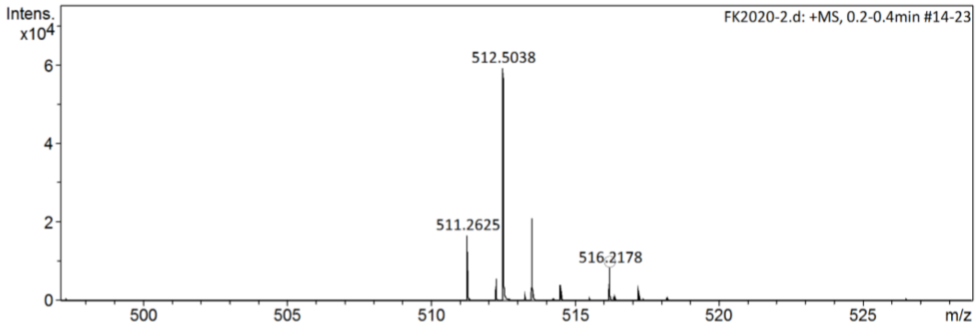

Meas. m/z	#	Ion Formula	Sum Formula	m/z	Adduct	err [ppm]	z	mSigma	Score	rdb	N-Rule	e⁻ Conf
516.2178	1	C29H35NNaO4S	C29H35NO4S	516.2179	M+Na	0.3	1+	54.5	100.00	13.0	ok	even

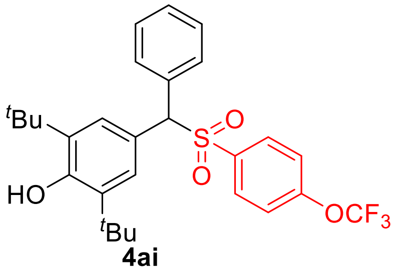

4ai

Acquisition Parameter

Source Type	ESI	Ion Polarity	Positive	Set Nebulizer	0.4 Bar
Focus	Not active	Set Capillary	4500 V	Set Dry Heater	180 °C
Scan Begin	50 m/z	Set End Plate Offset	-500 V	Set Dry Gas	4.0 l/min
Scan End	1300 m/z	Set Charging Voltage	2000 V	Set Divert Valve	Source
		Set Corona	0 nA	Set APCI Heater	0 °C

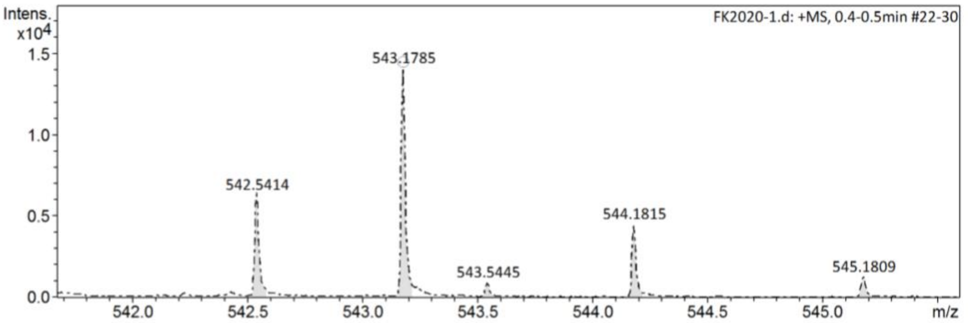

Meas. m/z	#	Ion Formula	Sum Formula	m/z	Adduct	err [ppm]	z	mSigma	Score	rdb	N-Rule	e⁻ Conf
543.1785	1	C28H31F3NaO4S	C28H31F3O4S	543.1787	M+Na	0.5	1+	12.6	100.00	12.0	ok	even

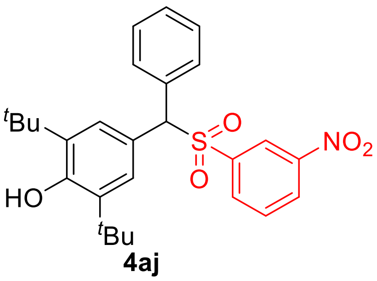

4aj

Acquisition Parameter

Source Type	ESI	Ion Polarity	Positive	Set Nebulizer	0.4 Bar
Focus	Not active	Set Capillary	4500 V	Set Dry Heater	180 °C
Scan Begin	50 m/z	Set End Plate Offset	-500 V	Set Dry Gas	4.0 l/min
Scan End	1300 m/z	Set Charging Voltage	2000 V	Set Divert Valve	Source
		Set Corona	0 nA	Set APCI Heater	0 °C

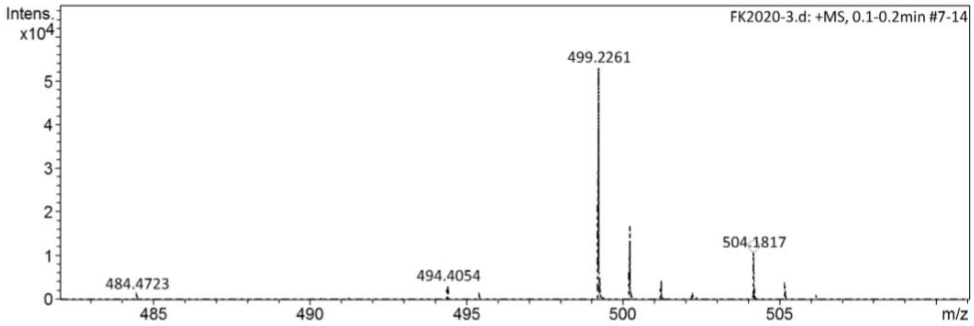

Meas. m/z	#	Ion Formula	Sum Formula	m/z	Adduct	err [ppm]	z	mSigma	Score	rdb	N-Rule	e⁻ Conf
504.1817	1	C27H31NNaO5S	C27H31NO5S	504.1815	M+Na	-0.3	1+	14.8	100.00	13.0	ok	even

6. Crystal data and structure refinement for 4af

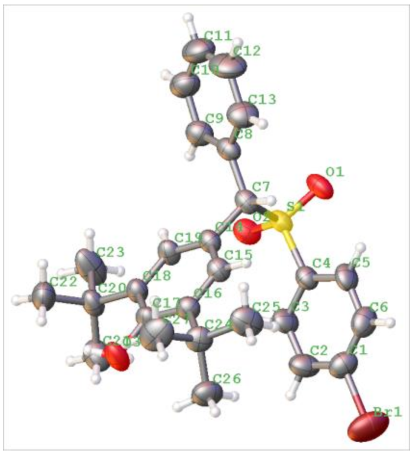

≡

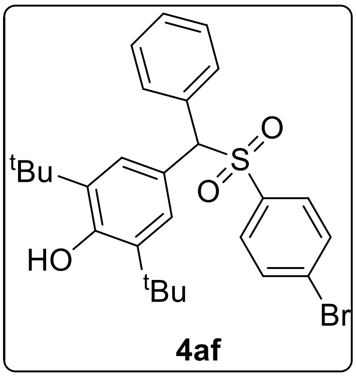

Empirical formula	$C_{27}H_{31}BrO_3S$
Formula weight	515.49
Temperature/K	290.36(10)
Crystal system	orthorhombic
Space group	$P2_12_12_1$
a/Å	10.8798(2)
b/Å	10.9903(3)
c/Å	21.4683(6)
α/°	90.0

β/°	90.0
γ/°	90.0
Volume/Å3	2567.01(11)
Z	4
ρcalcg/cm^3	1.334
μ/mm^{-1}	3.137
F (000)	1072.0
Crystal size/mm^3	0.54 × 0.52 × 0.21
Radiation	CuK_α (λ = 1.54184)
2Θ range for data collection/°	8.236 to 134.136
Index ranges	$-12 \leq h \leq 12$, $-13 \leq k \leq 5$, $-18 \leq l \leq 25$
Reflections collected	9789
Independent reflections	4538 [R_{int} = 0.0332, R_{sigma} = 0.0409]
Data/restraints/parameters	4538/1/296
Goodness-of-fit on F2	1.042
Final R indexes [I>=2σ (I)]	R_1 = 0.0417, wR_2 = 0.1083
Final R indexes [all data]	R_1 = 0.0441, wR_2 = 0.1114
Largest diff. peak/hole / e Å^{-3}	0.16/-0.38
Flack parameter	-0.006(16)
